# Resistance Gene-Guided Discovery of a Fungal Spirotetramate as an Acetolactate Synthase Inhibitor

**DOI:** 10.1021/jacs.5c16272

**Published:** 2025-10-28

**Authors:** Tsz Ki Chan, Xingxing Wei, Li Ma, Hang Wang, Pan Liao, Yudai Matsuda

**Affiliations:** † Department of Chemistry, 53025 City University of Hong Kong, Tat Chee Avenue, Kowloon, Hong Kong SAR, China; ‡ Department of Biology, 26679 Hong Kong Baptist University, Kowloon Tong, Hong Kong SAR, China; § School of Life Science and Technology, 56651 China Pharmaceutical University, Nanjing 210009, China; ∥ State Key Laboratory of Agrobiotechnology (CUHK), Shatin, New Territories, Hong Kong SAR, China; ⊥ AoE Centre for Plant Vacuole Biology and Biotechnology, The Chinese University of Hong Kong, Shatin, New Territories, Hong Kong SAR, China; # Institute of Systems Medicine and Health Sciences, Hong Kong Baptist University, Kowloon Tong, Hong Kong SAR, China

## Abstract

Biosynthetic gene clusters (BGCs) of bioactive natural products occasionally encode resistant versions of the proteins they inhibit, offering opportunities for resistance gene-guided genome mining to uncover natural products with predictable modes of action. In this study, we developed a genome mining tool designed to identify fungal BGCs harboring putative resistance genes. Applying this tool to approximately 2500 fungal genomes, we identified a BGC designated as the *pts* cluster, which encodes an acetolactate synthase (ALS) homologue. Functional characterization of the *pts* cluster resulted in the identification of pterrespiramide A (**1**), featuring unique spirotetramate and *cis*-decalin moieties. Consistent with the predicted activity, **1** was confirmed as an ALS inhibitor and exhibited both antifungal and herbicidal activities. This study illuminates the potential of resistance gene-guided genome mining as a powerful strategy for accelerating the discovery of previously undescribed bioactive natural products.

## Graphical abstract

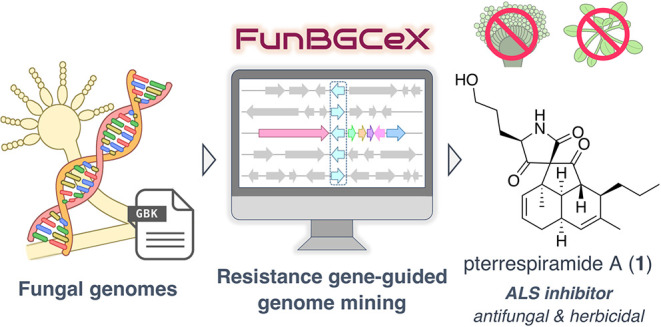

## Introduction

Natural products have long served as a privileged source for the discovery of pharmaceutical drugs and other functional substances, owing to their diverse range of biological activities and unique molecular architectures.^1^ These biological activities often result from the inhibition of proteins essential for fundamental cellular functions or primary metabolism.^2^ However, since such housekeeping proteins are widely conserved among organisms, bioactive metabolites may also be toxic to their producers. Consequently, self-resistance mechanisms must exist to protect the producing organisms from these toxic compounds.^3^ One well-known mechanism involves the expression of transporter proteins that export the toxic metabolites out of the cell; accordingly, many natural product biosynthetic gene clusters (BGCs) contain one or more transporter genes.^4^

Intriguingly, some BGCs of bioactive natural products encode a close homologue of the housekeeping protein inhibited by the natural product, which is resistant to the toxic compound and can maintain cellular processes even in its presence.^3b,5^ This phenomenon is also observed in the BGCs for several clinically relevant molecules (Figure 1). For example, the cholesterol-lowering agent lovastatin inhibits 3-hydroxy-3-methylglutaryl-CoA (HMG-CoA) reductase, which is the rate-limiting enzyme in cholesterol biosynthesis, and its BGC encodes an HMG-CoA reductase homologue.^6^ Likewise, the BGCs of the immunosuppressant medications mycophenolic acid and cyclosporin A encode an inosine 5′-monophosphate dehydrogenase (IMPDH) and cyclophilin—their respective target proteins.^7^ Given that the presence of a housekeeping protein within a BGC is relatively rare, natural products derived from such BGCs might hold greater promise for clinical drug development.

**Figure 1:**
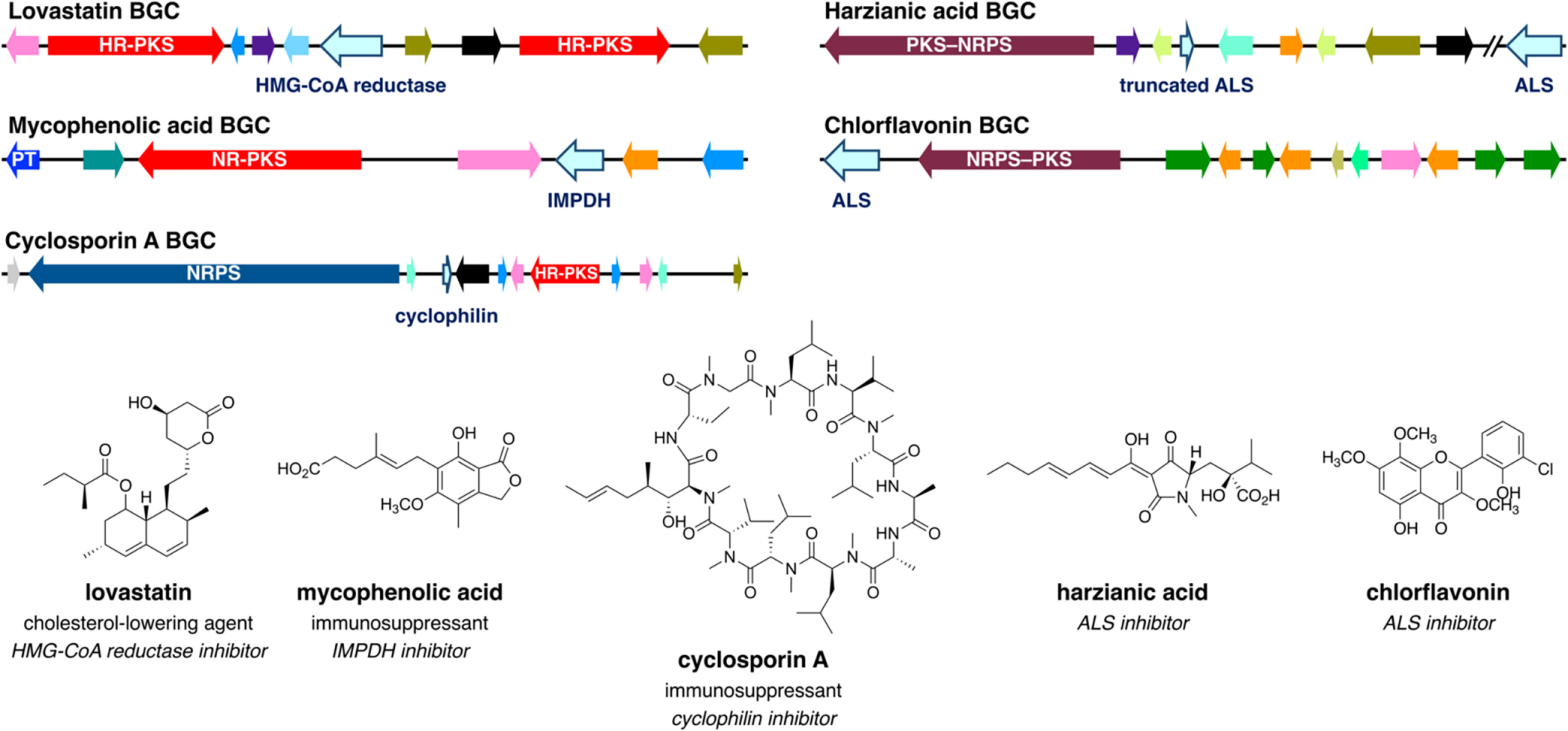
Selected fungal biosynthetic gene clusters (BGCs) with resistance genes and their products, including clinically relevant molecules (lovastatin, mycophenolic acid, and cyclosporin A) and acetolactate synthase (ALS) inhibitors (harzianic acid and chlorflavonin). Note that the BGC of harzianic acid encodes a truncated ALS and that the resistant version of ALS is encoded outside the cluster. Only core biosynthetic enzyme genes and resistance genes are labeled for clarity. HR-PKS: highly reducing polyketide synthase; NR-PKS: nonreducing polyketide synthase; PT: prenyltransferase; NRPS: nonribosomal peptide synthetase.

Searching for BGCs encoding a close homologue of housekeeping proteins offers an opportunity to discover natural products with predictable biological activities.^8^ Alongside the accumulation of microbial genome sequence data, resistance gene-guided genome mining has now been widely adopted, leading to the discovery of new bioactive molecules^9^ and the identification of molecular targets of known natural products.^10^ Accordingly, bioinformatic tools that facilitate resistance gene-directed genome mining, such as FRIGG^11^ and ARTS/FunARTS,^12^ have been developed. Nevertheless, given the large number of unexploited BGCs in microorganisms, many BGCs likely encode resistance proteins against previously undescribed bioactive compounds.

In this study, to discover bioactive natural products, we first updated our fungal genome mining tool, FunBGCeX,^13^ to perform resistance gene-guided genome mining. By utilizing this tool, we focused on a BGC encoding an acetolactate synthase (ALS) homologue. Characterization of this BGC led to the discovery of a compound named pterrespiramide A (**1**), consisting of spirotetramate and decalin moieties. The compound exhibits antifungal and herbicidal activities and was, as expected, confirmed as an ALS inhibitor. Our study demonstrates the utility of our genome mining tool and the effectiveness of resistance gene-guided genome mining in discovering bioactive natural products.

## Results

### Development of a Tool for Resistance Gene-Guided Genome Mining in Fungi

We initially sought to add a function to our recently developed genome mining tool, FunBGCeX,^13^ to facilitate resistance gene-directed genome mining in fungi (Figures 2A and S1). To this end, we focused on the proteins encoded by the genome of *Aspergillus fumigatus* Af293 and, based on comparisons with those in other organisms, selected 1925 highly conserved proteins considered to be possible housekeeping proteins (Supporting Data 1). These proteins are used as hooks for genome mining, and only BGCs encoding a close homologue of these *A. fumigatus* proteins, with the homologue duplicated in the genome, are extracted. Furthermore, although not directly relevant to the present study, we also created a database of human proteins classified as “FDA approved drug targets” or “Disease related genes” in the Human Protein Atlas,^14^ for future genome mining specifically targeting human protein homologues.

**Figure 2:**
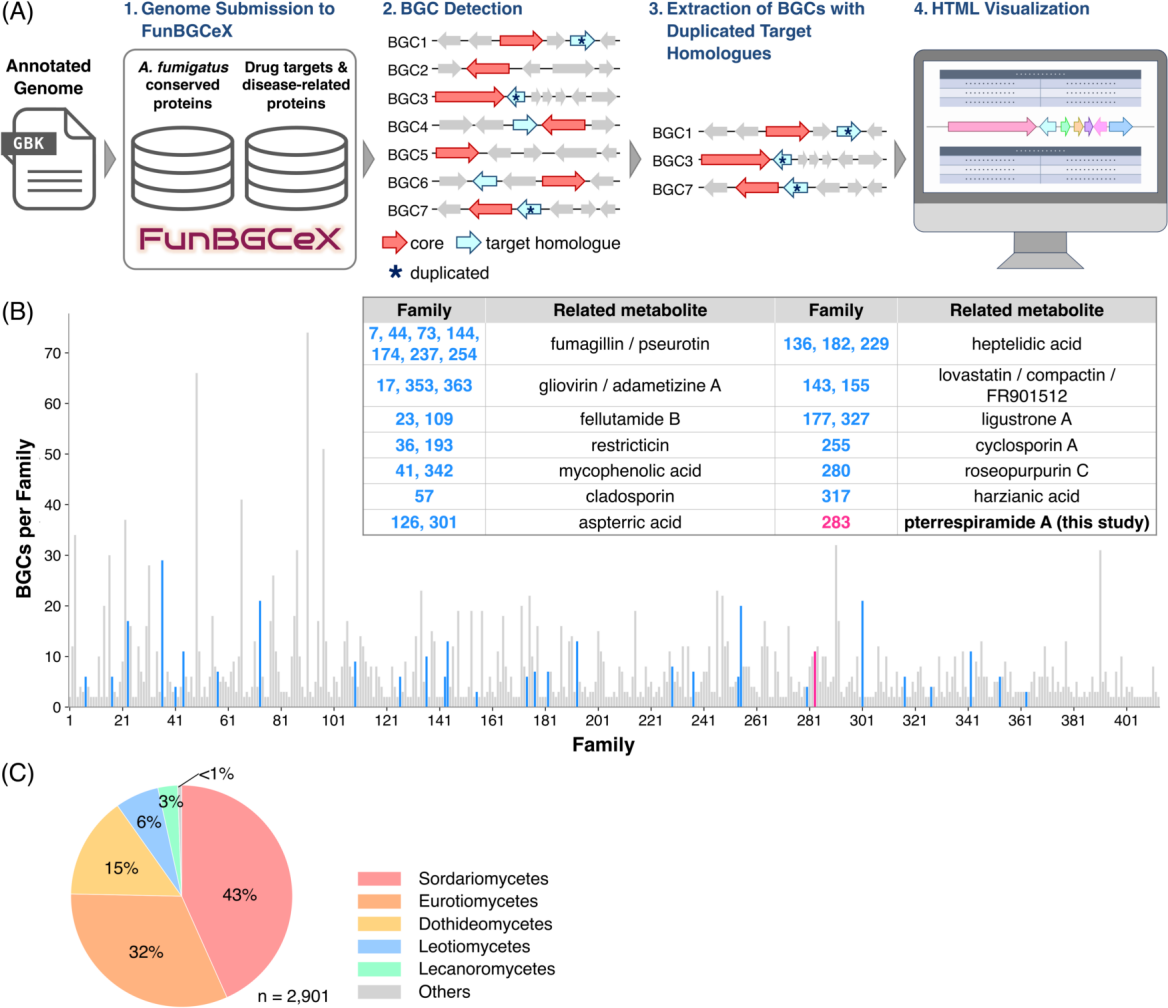
(A) General workflow of resistance gene-guided genome mining using FunBGCeX. Example HTML visualization output is shown in Figure S1. The FunBGCeX logo was created by the author and is used with permission. (B) Summary of biosynthetic gene clusters (BGCs) detected in this study, grouped into families. Families corresponding to BGCs with similarity to known clusters with resistance genes are indicated in blue, whereas the family of interest is indicated in pink. These highlighted families are also shown in the accompanying table. (C) Distribution of all BGCs classified into families according to the fungal taxonomic class from which they originate.

To examine whether this improved version of FunBGCeX works as expected, we analyzed the genome of *Aspergillus terreus* NIH 2624, since *A. terreus* produces several bioactive compounds whose BGCs encode self-resistance enzymes.^6,9a,15^ As a result, eight BGCs were extracted (Figure S2), including those for three known bioactive compounds: lovastatin (BGC1),^6^ citreoviridin (BGC2),^15^ and aspterric acid (BGC8).^9a^ We noted that one of the other BGCs (BGC3) is highly similar to the *apm* cluster responsible for asperphenamate biosynthesis.^16^ The *apm* cluster encodes a close homologue of phospho-2-dehydro-3-deoxyheptonate aldolase ApmC, which has been shown not to be directly involved in asperphenamate biosynthesis.^16^ Thus, ApmC might be a self-resistance protein, although its detailed function remains to be clarified. Moreover, another BGC (BGC6) containing a homologue of sphingolipid C4-hydroxylase was found. Since the BGC is not closely related to known clusters, it might produce a new bioactive compound. Overall, although some false-positive cases may be included, we judged that our genome mining tool possesses sufficient ability to detect BGCs with possible resistance proteins.

### Global Genome Mining in Pezizomycotina Fungi and Selection of a Biosynthetic Gene Cluster for Experimental Characterization

To discover new bioactive natural products via resistance gene-guided genome mining, we downloaded 2544 reference genomes of Pezizomycotina fungi, including well-known genera such as *Aspergillus*, *Penicillium*, and *Fusarium*, from the NCBI database (Supporting Data 2). These genomes were analyzed using FunBGCeX, and the extracted BGCs were grouped into gene cluster families using BiG-SCAPE.^17^ Some families contained BGCs with different core enzymes or housekeeping protein homologues; therefore, further classification was performed to ensure that each family comprised only BGCs sharing the same core enzymes and housekeeping proteins. Families composed solely of BGCs from a single genus were subsequently excluded to eliminate potential false positives, resulting in 413 gene cluster families encompassing 2901 BGCs (Supporting Data 3). While some families included BGCs homologous to known clusters containing resistance proteins, many could not be readily linked to reported metabolites (Figure 3B). Notably, the majority of identified BGCs originated from fungi in the class Sordariomycetes, although proteins from *A. fumigatus* (Eurotiomycetes) were used to construct the database of putative housekeeping enzymes (Figure 3C), suggesting the broad applicability of our genome mining tool.

**Figure 3:**
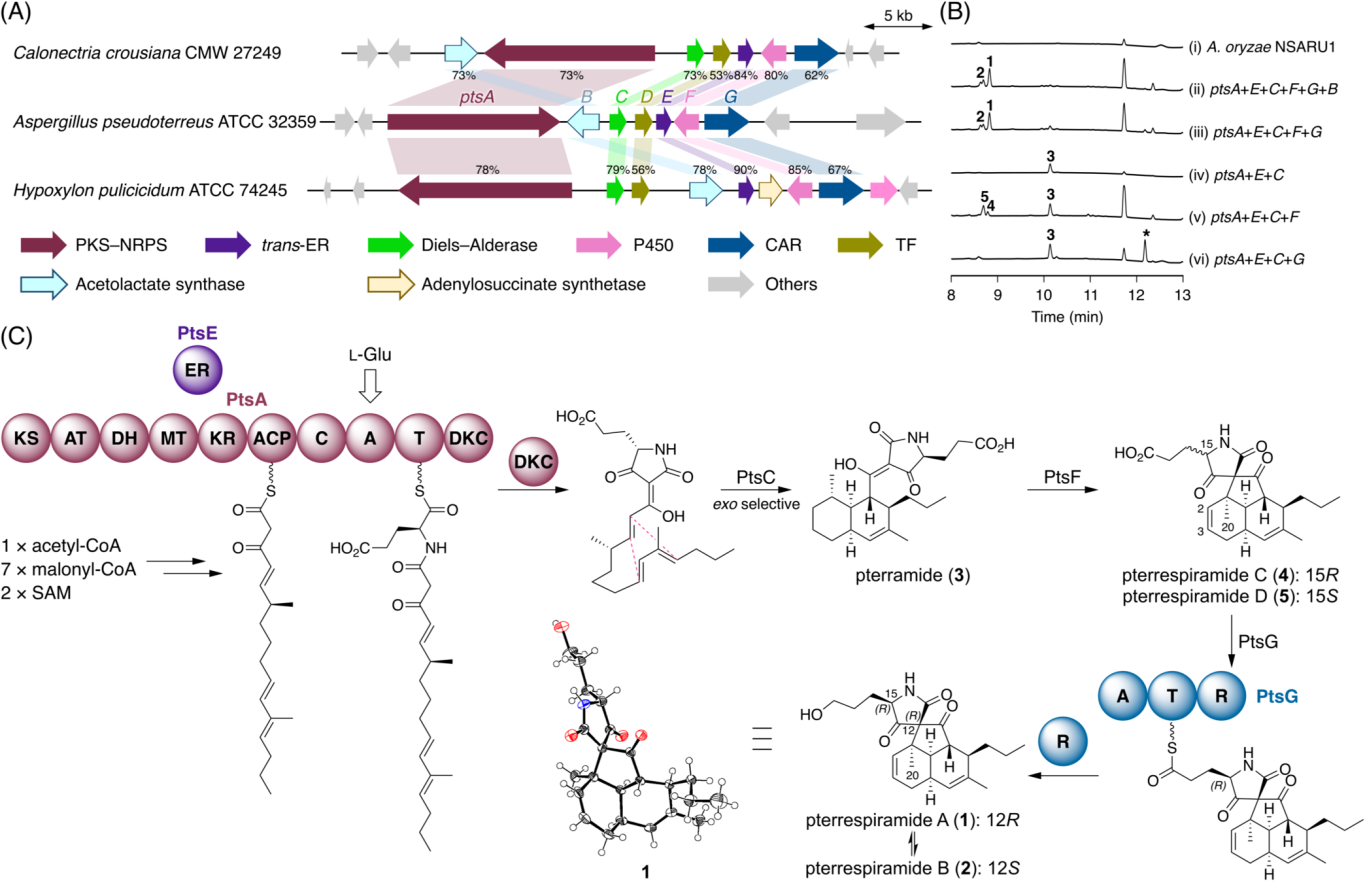
(A) Schematic representation of the *pts* cluster and its comparison with homologous clusters from fungi in different genera. The sequence identity values of each gene product are given. (B) HPLC profiles of the metabolites produced by *A. oryzae* NSARU1 transformants. The asterisk indicates an unstable metabolite that cannot be isolated in this study. The chromatograms were monitored at 190 nm. (C) Biosynthetic pathway and crystal structure of pterrespiramide A (**1**).

Among the families lacking significant similarity to known clusters, we focused on Family 283, which comprises 11 BGCs derived from three genera: *Aspergillus*, *Hypoxylon*, and *Calonectria*. Each of these BGCs encodes a close homologue of acetolactate synthase (ALS; also known as acetohydroxyacid synthase, AHAS). ALS catalyzes the first step in branched-chain amino acid biosynthesis and is a well-known target of antimicrobial and herbicidal agents.^18^ While ALS homologues have been identified in the BGCs of two known natural products, harzianic acid^19^ and chlorflavonin^20^ (Figure 1), Family 283 BGCs are not closely related to these clusters. Consequently, we hypothesized that Family 283 BGCs might produce a previously unreported ALS inhibitor.

Subsequently, comparison of three representative BGCs, one from each genus, revealed seven genes that are highly conserved among the clusters. Notably, these conserved genes show relatively limited occurrence across fungal genomes, whereas many of the adjacent genes are more widely distributed, thereby allowing us to delineate the BGC boundaries (Figure 3A). In the following experimental characterization, we focused on the BGC from *Aspergillus pseudoterreus* ATCC 32359, designated as the *pts* cluster (Tables S1 and S2). In addition to the ALS PtsB, the *pts* cluster encodes the polyketide synthase–nonribosomal peptide synthetase (PKS–NRPS) PtsA, the *trans*-acting enoylreductase (*trans*-ER) PtsE, the Diels–Alderase PtsC, the cytochrome P450 monooxygenase PtsF, the NRPS-like carboxylic acid reductase (CAR) PtsG, and the transcription factor (TF) PtsD. Interestingly, the BGCs from *Hypoxylon pulicicidum* ATCC 74245 and other *Hypoxylon* fungi also encode a homologue of adenylosuccinate synthetase, which is a key enzyme in purine nucleotide biosynthesis.^21^ Given that the BGC from *H. pulicicidum* encodes an additional P450 enzyme (Figure 3A), this cluster could potentially produce two distinct bioactive compounds.

### Heterologous Expression of the *pts* Cluster

To obtain metabolites derived from the *pts* cluster, we performed heterologous expression of the BGC using the *Aspergillus oryzae* NSARU1 strain.^22^ In our experiments, *A. pseudoterreus* CBS 116.46 (=ATCC 10020), the parental strain of *A. pseudoterreus* ATCC 32359, served as the source of the *pts* cluster due to its easier accessibility to us. Initially, all five biosynthetic enzyme-encoding genes (*ptsA*, -*C*, -*E*, -*F*, and -*G*), along with the ALS gene *ptsB*, were coexpressed in *A. oryzae*. HPLC analysis of the metabolites from the transformant revealed the presence of a major product **1**, which was absent in the host strain (Figure 3B, traces i and ii). Compound **1** was successfully isolated through chromatographic steps, and its molecular formula was determined as C_23_H_31_NO_4_ by HR–MS analysis. NMR analysis revealed that **1** contains a decalin system and a tetramate moiety with a 3-hydroxypropyl side chain. Furthermore, the decalin and tetramate units were found to be spiro-fused via a cyclopentanone ring, establishing the planar structure of **1** (Figure 3C). Regarding stereochemistry, the relative configuration of the *cis*-fused decalin moiety was determined based on the ^1^H–^1^H coupling constants and NOESY analysis. However, NMR analysis alone was insufficient to establish the complete relative configuration of **1**. Thus, we applied the advanced Marfey’s method^23^ using 5-hydroxynorvaline standards, confirming the 15*R* configuration (Figure S3). Nevertheless, the configuration of the spiro center and the absolute configuration of **1** remained unsolved. Gratifyingly, **1** was successfully crystallized and subjected to single-crystal X-ray diffraction analysis (Figure 3C; CCDC 2487927), which, together with the Marfey’s method results, revealed the complete structure of **1**. Compound **1** was named pterrespiramide A. We also confirmed the presence of **1** in *A. pseudoterreus* CBS 116.46 (Figure S4), further indicating that **1** is the genuine product of the *pts* cluster.

Additionally, we isolated an isomer of **1**, **2**, as a minor product (Figure 3B, trace ii). The NMR spectra of **2** were highly similar to those of **1**, revealing that **2** has the same planar structure as **1**. One notable difference was the presence of the NOSEY correlation between H-15 and H-20 in **2**. This observation suggested that **2** possesses the opposite configuration at C-12 or C-15. While this manuscript was in submission, another work reported **1**, along with its C-12 and C-15 epimers.^24^ Based on the reported NMR data of these compounds, we determined that **2** is the C-12 epimer of **1**. Notably, we observed nonenzymatic interconversion between **1** and **2** in the fungal culture medium (DPY liquid medium) (Figure S5). Compound **2** was designated as pterrespiramide B.

To investigate the importance of the ALS PtsB, we constructed an *A. oryzae* transformant expressing five biosynthetic genes but lacking *ptsB*. The metabolic profile of this transformant was similar to that of the transformant with *ptsB* (Figure 3B, trace iii), making it unclear whether PtsB confers self-resistance. The lack of apparent toxicity of **1** to *A. oryzae* might be attributed to the abundance of transporter genes in the fungus,^25^ which could provide intrinsic resistance to **1**. Although further investigations are required to clarify the self-resistance mechanism in *A. oryzae*, this observation demonstrates that PtsB is not directly involved in the biosynthesis of **1**, consistent with the expectation that PtsB functions as a resistance protein.

### Investigation of the Biosynthetic Pathway of Pterrespiramide A

Next, we turned our attention to the biosynthetic route leading to pterrespiramide A (**1**). Since the PKS–NRPS PtsA, the *trans*-ER PtsE, and the Diels–Alderase PtsC are predicted to collaboratively produce the first stable pathway intermediate,^26^ an *A. oryzae* transformant harboring *ptsA*, *ptsE*, and *ptsC* was constructed, which yielded a new major metabolite **3** (Figure 3B, trace iv). Structural analysis revealed that **3** contains a *cis*-fused decalin moiety, as observed in **1**, and a tetramate moiety with a 2-carboxyethyl side chain, but lacks the spiro ring system as well as the double bond between C-2 and C-3 (Figure 3C). Interestingly, advanced Marfey’s analysis^23^ showed that **3** possesses the 15*S* configuration (Figure S3), opposite to that of **1**. This suggests that l-glutamate is utilized for the biosynthesis of **3**, and that stereoinversion occurs during the conversion leading to **1**. Compound **3** was named pterramide.

The following biosynthetic step is predicted to be catalyzed either by the P450 PtsF or the CAR PtsG. Since it was unclear which enzyme acts first, we constructed two *A. oryzae* transformants expressing four genes, each harboring either *ptsF* or *ptsG*. The transformant expressing the P450 gene *ptsF* produced a pair of isomers, **4** and **5**, which were not observed in the transformant expressing only the three genes (Figure 3B, trace v). The NMR spectra of **4** and **5** closely resemble those of **1** and **2**; however, the hydroxymethyl signals for C-19 disappeared in both the ^1^H and ^13^C NMR spectra, and a new carboxy signal appeared in the ^13^C NMR spectrum. Additionally, the NOESY correlation between H-15 and H-20 was only observed in **5**, as was the case for **2**. Further comparison with the recently reported data of all four possible diastereomers^24^ determined that **4** was the oxidized form of **1**, featuring a C-19 carboxy group, and that **5** was the C-15 isomer of **4** (Figure 3C). Compounds **4** and **5** were designated as pterrespiramides C and D, respectively. Meanwhile, the transformant expressing *ptsG* yielded another metabolite (Figure 3B, trace vi), whose molecular formula was deduced to be C_23_H_33_NO_4_ by HR–MS analysis. Although this molecular formula corresponds to the aldehyde form of **3**, we were unable to isolate the metabolite in pure form, likely due to its instability, which also resulted in complicated NMR spectra.

On the basis of the experimental results described above, it is reasoned that the P450 PtsF is responsible for the oxidative spiro ring formation as well as the desaturation at C-2/C-3, whereas the CAR PtsG performs the reduction of the C-19 carboxy group to an alcohol. However, one of the key remaining questions in the biosynthetic process is how the end product **1** with the 15*R* configuration is formed from **3**, its precursor with the 15*S* configuration. First, we noted that the P450 products, **4** and **5**, undergo rapid epimerization in DPY liquid medium (Figure S5). Importantly, this epimerization occurs much faster than that between **1** and **2**. Collectively, the direct product of PtsF would be **5** with the 15*S* configuration, but **5** can be nonenzymatically converted to **4**. Next, we focused on the detailed function of the CAR PtsG. We initially aimed to investigate the substrate specificity of the adenylation (A) domain, which, however, was found to be difficult due to the rapid epimerization between **4** and **5** in all reaction buffer systems used in this study. Thus, we then sought to reconstitute the complete reaction catalyzed by PtsG. Since our attempt to obtain full-length PtsG was unsuccessful, we separately purified the A domain and the thiolation (T)–reductase (R) didomain as N-terminal His-tagged proteins using an *Escherichia coli* expression system. The in vitro enzymatic reaction of either **4** or **5** with the A domain, the T–R didomain, the promiscuous phosphopantetheinyl transferase Sfp,^27^ CoA, ATP, Mg^2+^, and NADPH afforded **1** as a major product regardless of the substrate used for the reaction, with no detectable production of the corresponding aldehyde form (Figure 4, traces iii to vi). Meanwhile, **3** was not converted to any products, including the uncharacterized metabolite detected in the *A. oryzae* transformant expressing *ptsA*, *ptsE*, *ptsC*, and *ptsG*, under the same reaction conditions (Figure 4, traces i and ii). This observation indicates that the CAR PtsG acts after the P450 PtsF. The unstable product observed in the heterologous expression experiment could possibly be synthesized through the combined action of PtsG and an endogenous enzyme in *A. oryzae*.

**Figure 4:**
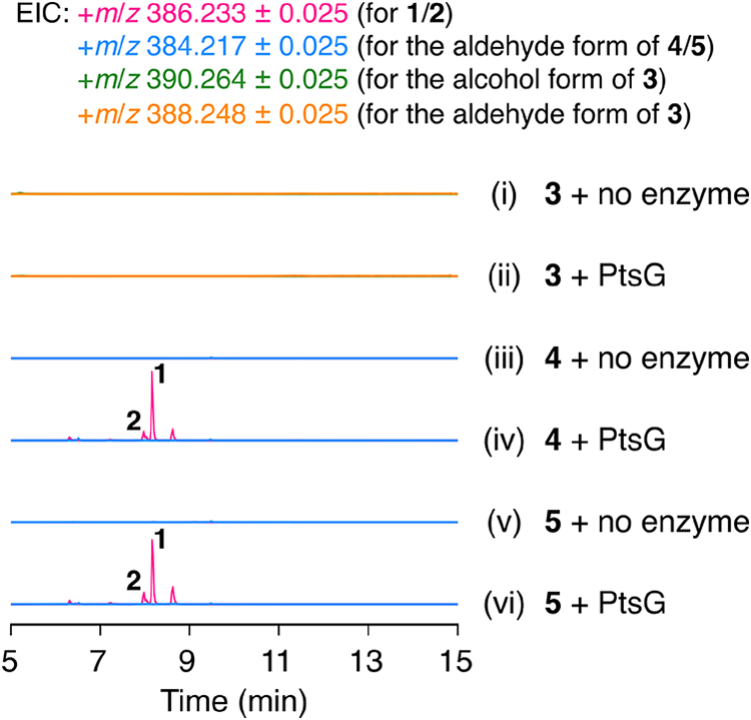
LC–MS profiles of the in vitro enzymatic reactions with PtsG. PtsG was provided as two separate proteins, the A domain and the T–R didomain.

### Biological Activities of Pterrespiramide A as an Acetolactate Synthase Inhibitor

Having established the biosynthetic pathway leading to pterrespiramide A (**1**), we next examined whether **1** functions as an acetolactate synthase (ALS) inhibitor. To this end, we employed a yeast-based assay in which the inhibitory activities of **1** were compared against three *Saccharomyces cerevisiae* INVSc1 transformants (Figure 5A). Compound **1** effectively inhibited the growth of the yeast transformant harboring an empty vector, with an IC_50_ value of 0.4 μM, which is comparable to that of the commercially available herbicide and ALS inhibitor sulfometuron methyl (IC_50_ = 0.6 μM) (Figure S6). By comparison, the yeast strain carrying an additional copy of *ilv2*, encoding ALS, exhibited greater resistance to **1**, resulting in an increased IC_50_ value of 1.5 μM. As expected, the transformant expressing *ptsB*, predicted to encode a resistant version of ALS, showed clear resistance to **1**. Collectively, these results demonstrate that **1** targets ALS and that *ptsB* functions as a self-resistance gene. Meanwhile, pterrespiramide B (**2**), the C-12 epimer of **1**, displayed weaker antifungal activity against *S. cerevisiae* INVSc1, with an IC_50_ value of 5.8 μM (Figure S6). Conversely, compounds **3**–**5** did not exhibit apparent antifungal activity against the yeast strain. To further verify the molecular target of **1**, we conducted in vitro enzymatic assays using *S. cerevisiae* ALS (ScALS), purified from the *E. coli* expression system, in the presence of different concentrations of **1** or **2**. Both compounds inhibited ScALS activity in a dose-dependent manner, with **1** being more potent (Figure S7), further corroborating that **1** is an ALS inhibitor.

**Figure 5:**
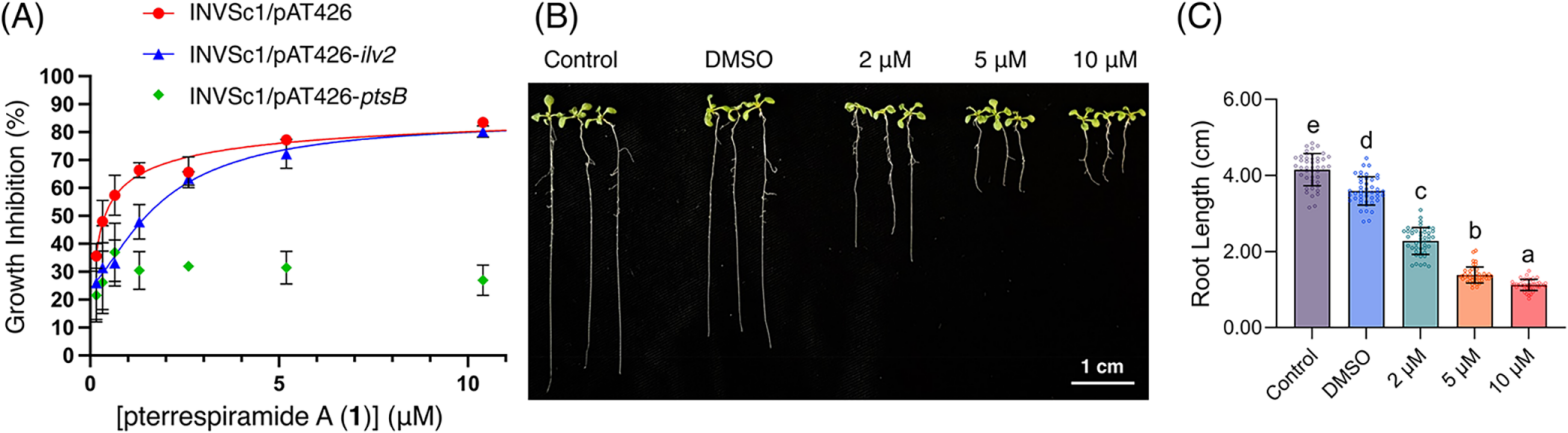
Evaluation of the biological activity of pterrespiramide A (**1**) as an acetolactate synthase inhibitor. (A) Growth inhibition of *S. cerevisiae* transformants by **1**. (B) *Arabidopsis* primary roots after treatment with **1** for 5 days at different concentrations. Scale bar = 1 cm. (C) Quantification of root length after treatment with **1**. Significance was determined by one-way ANOVA with Tukey’s multiple comparisons test. Data are shown as the means ± SD (*n* = 45).

Given that ALS is an essential enzyme in microorganisms and plants, we further evaluated the antimicrobial and herbicidal potential of **1** and the other compounds obtained in this study. While none of the compounds **1**–**5** displayed antibacterial activity against the tested strains, **1** and **2** exhibited moderate antifungal activity against the pathogenic fungus *A. fumigatus*, with MIC values of 7.7 and 30.8 μg/mL (20 and 80 μM), respectively. Furthermore, the primary root length of 5- to 10-day-old *Arabidopsis* seedlings was significantly inhibited by compound **1** (2, 5, and 10 μM) in a dose-dependent manner (Figures 5B,C, S8, and S9). We tested the effect of the compound **1** on root growth under 16 h light and 8 h dark cycles. After 5 days of treatment with the compound **1**, the root length in the untreated control group reached 4.15 ± 0.421 cm from about 1 cm (Figure S8), while the root length in the group treated with only DMSO was 3.59 ± 0.371 cm, which is slightly reduced from the untreated control group (Figure 5B,C). With 2 μM of **1**, the root length was 2.28 ± 0.352 cm; with 5 μM, the root length was 1.38 ± 0.211 cm; and with 10 μM, the root length was 1.12 ± 0.145 cm, showing a significant reduction in primary root growth compared to the untreated control and DMSO groups (Figure 5B,C). Further statistical analysis of the data indicated that the compound at 2 μM significantly inhibited *Arabidopsis* primary root growth, and concentrations of 5 to 10 μM had an even greater inhibitory effect on primary root growth (Figure 5B,C). Taken together, these results demonstrate that the compound **1** inhibits *Arabidopsis* primary root growth in a dose-dependent manner.

## Discussion

The discovery of pterrespiramide A (**1**) demonstrates the potential of resistance gene-guided genome mining to uncover previously undescribed bioactive natural products. Although this strategy has been widely adopted over the past decade, one key limitation of this approach is the frequent rediscovery of known compounds following the characterization of mined BGCs, particularly in fungal genome mining. Fortunately, we obtained a new bioactive natural product with an intriguing molecular skeleton in this study, suggesting that many more unexploited bioactive compounds can be discovered by characterizing additional BGCs identified in our current work. For example, although ALS has been found as a resistance protein in three different types of BGCs,^19,20^ our global genome mining revealed more BGCs encoding close homologues of ALS (Figure S10). BGCs in Family 107 include large gene clusters encoding an ALS homologue as well as two or three NRPSs and one PKS, whereas those in Families 39 and 296 contain an ALS gene together with an NRPS-like enzyme. Given that these BGCs do not closely resemble known clusters, their characterization may lead to the discovery of previously unreported ALS inhibitors. The wide occurrence of ALS-containing BGCs across diverse fungal taxa suggests that these clusters may play important roles in interspecies competition. Furthermore, FunBGCeX outputs also include BGCs with potential resistance genes other than ALS, which might be responsible for the formation of novel bioactive molecules. Notably, FunARTS,^12c^ a recently developed tool for resistance gene-based genome mining, failed to recognize *ptsB*, the resistance gene in the *pts* cluster, as a resistance gene (Figure S11). Thus, the combined use of FunARTS and FunBGCeX may enhance the discovery of fungal bioactive natural products.

The molecular architecture of pterrespiramides represents an unprecedented natural product scaffold. The biosynthetic route leading to pterrespiramide A (**1**) can be proposed as follows (Figure 3C). The PKS–NRPS PtsA and the *trans*-ER PtsE collaboratively produce a tetramate-containing intermediate with a linear carbon chain. The Diels–Alderase PtsC catalyzes an exoselective [4 + 2] cycloaddition to yield pterramide (**3**) featuring a *cis*-decalin moiety. Compound **3** then undergoes two rounds of oxidative reactions catalyzed by PtsF to form pterrespiramides C (**4**) and D (**5**), either or both of which are accepted by PtsG to selectively afford **1**.

The reactions catalyzed by the P450 enzyme PtsF involve desaturation at C-2/C-3 and oxidative spiro ring formation. Since we were unable to isolate the product of the first step, we cannot definitively conclude which reaction occurs first; however, given that installation of the spiro system causes a significant structural change, double bond formation likely precedes spiro ring formation to produce compound **A** (Figure 6). The mechanism for subsequent spirotetramate formation can be proposed based on a similar P450-catalyzed C–C bond formation observed in the synthesis of wigandol,^28^ and on the general observation that many P450-catalyzed C–C bond formations proceed via a diradical intermediate^29^ (Figure 6). Initial hydrogen abstraction by compound I may occur either at the enolic hydrogen or at C-1, generating the substrate radical **B** or **C**, respectively, followed by an additional hydrogen abstraction from the other site, yielding the diradical species **D**. Finally, C–C bond formation between C-1 and C-12 completes the spiro ring formation to afford **5**. Nevertheless, alternative reaction mechanisms, such as those involving electron transfer to generate carbocationic species and C–C bond formation by radical attack (Figure S12), cannot be excluded at this stage. Therefore, further investigations, including theoretical calculations, are required to clarify the mechanism of this unusual transformation. A similar spirotetramate system can be found in the structures of altercrasins,^30^ and therefore, their biosynthesis might also involve a P450-catalyzed oxidative spiro-ring formation. Meanwhile, **5** can spontaneously interconvert with its C-15 epimer **4**. Considering that the interconversion between **4** and **5** occurs rapidly, it is likely that the carboxy group in **4** and **5** facilitates rapid epimerization. The carboxylate may act as a base to deprotonate at C-15, and subsequent reprotonation results in epimerization at this position (Figure S13).

**Figure 6:**
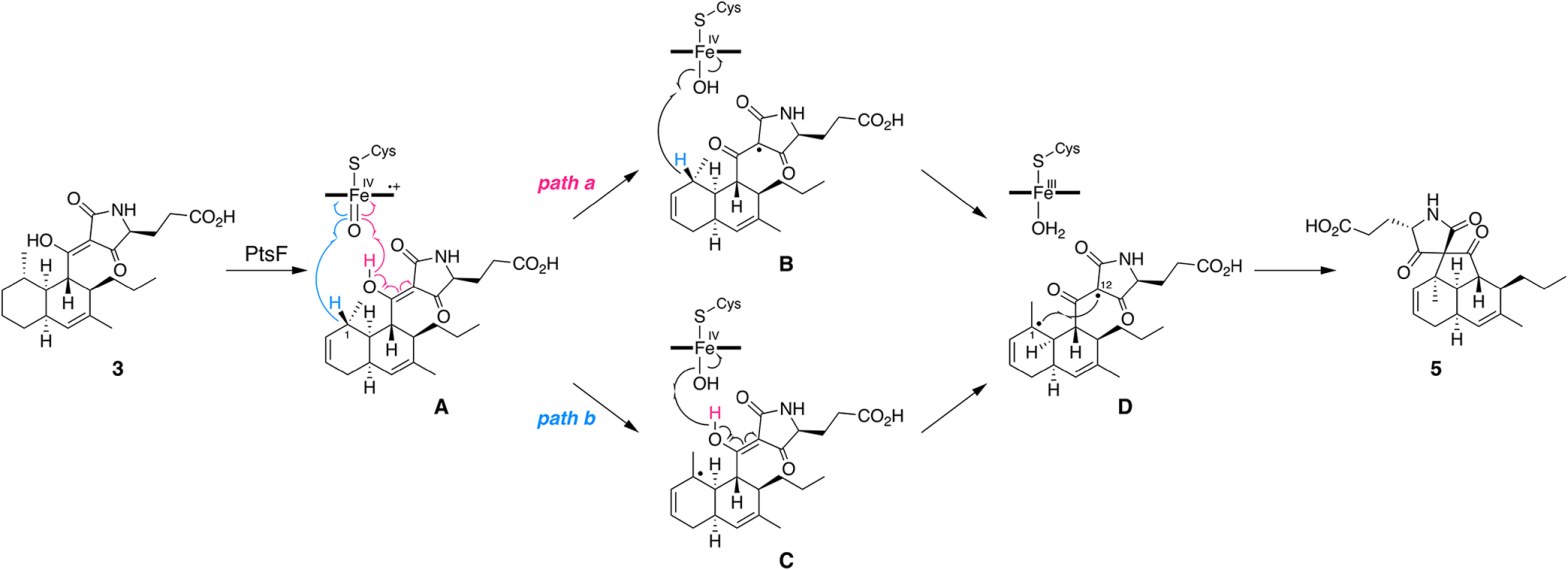
Proposed reaction mechanism for the PtsF-catalyzed spirotetramate formation. See Figure S11 for alternative possible mechanisms.

The final step of biosynthesis, catalyzed by the NRPS-like CAR PtsG, requires a four-electron reduction and results in the stereoselective formation of **1** with the 15*R* configuration. One possible explanation for the product selectivity is that the A domain of PtsG only accepts **4** with the 15*R* configuration; however, as mentioned above, we were unable to determine the substrate specificity of the A domain. Another possible scenario is that both **4** and **5** can be loaded onto the enzyme. In this case, online epimerization likely occurs prior to reduction by the R domain, which proceeds exclusively with the substrate bearing the 15*R* configuration. This epimerization may also be catalyzed by the R domain, analogous to those catalyzed by ketoreductase (KR) domains in modular type I PKSs.^31^ In any case, the R domain catalyzes a four-electron reduction to yield the alcohol **1** as the major product, which clearly contrasts with the general understanding that CAR enzymes perform a strict two-electron reduction.^32^ Thus, although *Nc*CAR, a CAR enzyme from the fungus *Neurospora crassa*, is known to be capable of four-electron reduction,^33^ PtsG represents a rare example in which a CAR enzyme performs four-electron reduction as its primary function. We noted that PtsG exhibits the highest similarity (39% protein sequence identity) with FSL6 encoded by the BGC of fusarielin H,^34^ according to the FunBGCs database. Although FSL6 was predicted not to be involved in fusarielin biosynthesis,^34^ given the requirement of four-electron reduction in the formation of fusarielin H, FSL6 may also serve as a CAR enzyme, similar to PtsG. Finally, considering that **4** and **5**, containing a carboxy group, did not display antifungal activity, the PtsG-catalyzed four-electron reduction with stereoinversion is a crucial step to afford a compound with the highest biological activity.

## Conclusions

In this study, we developed an improved fungal genome mining tool targeting biosynthetic gene clusters (BGCs) containing genes that encode potential self-resistance proteins by updating FunBGCeX. Using this platform, we successfully identified a previously undescribed acetolactate synthase (ALS) inhibitor, pterrespiramide A (**1**), which features a unique spirotetramate moiety and exhibits antifungal and herbicidal activities. Additionally, we identified biosynthetic enzymes with unique functions, including the P450 PtsF and the CAR PtsG, whose detailed characterization will be the subject of future investigations. Building upon this work, we will continue to characterize additional BGCs harboring diverse resistance genes, thereby facilitating the discovery of a wide range of bioactive molecules with potential applications in drug development.

## Supplementary Material









## Data Availability

All original codes for FunBGCeX (v1.0.0) are deposited at Zenodo under the DOI 10.5281/zenodo.17113445 and are also available at https://github.com/ydmatsd/funbgcex. CCDC 2487927 contains the supporting crystallographic data for this paper. These data can be obtained free of charge from The Cambridge Crystallographic Data Centre via www.ccdc.cam.ac.uk/structures. The other data underlying this study are available in the published article and its Supporting Information.

## Notes

Experimental details, analytical data, tables of gene cluster information, DNA and protein sequences, primer sequences, constructed plasmids, and *A. oryzae* transformants, figures of FunBGCeX outputs, HPLC/LC–MS profiles, graphs/photos for bioassay experiments, BGC comparisons, FunARTS output, reaction mechanisms, SDS-PAGE analysis, and NMR data/spectra, and references (PDF)
List of the selected proteins from *A. fumigatus* Af293 (Data S1) (XLSX)
List of fungal genomes used in this study (Data S2) (XLSX)
Summary of the BGCs extracted in this study (Data S3) (XLSX)
