# Supplementary material for: Resistance Gene-Guided Discovery of a Fungal Spirotetramate as an Acetolactate Synthase Inhibitor
Source: J Am Chem Soc. 2025 Oct 28;147(45):42100–9. doi: 10.1021/jacs.5c16272 (PMC12616698; doi:10.1021/jacs.5c16272)
Supplement: Supplementary file 1 [file ja5c16272_si_001.pdf]

Supporting Information for

Resistance Gene-Guided Discovery of a Fungal Spirotetramate as an Acetolactate Synthase Inhibitor

Tsz Ki Chan,[1] Xingxing Wei,[1] Li Ma,[2] Hang Wang,[3] Pan Liao,*[,2,4,5,6] and Yudai Matsuda*[,1]

[1]Department of Chemistry, City University of Hong Kong, Tat Chee Avenue, Kowloon, Hong Kong SAR, China

[2]Department of Biology, Hong Kong Baptist University, Kowloon Tong, Hong Kong SAR, China

[3]School of Life Science and Technology, China Pharmaceutical University, Nanjing 210009, China

[4]State Key Laboratory of Agrobiotechnology (CUHK), Shatin, New Territories, Hong Kong SAR, China

[5]AoE Centre for Plant Vacuole Biology and Biotechnology, The Chinese University of Hong Kong, Shatin, New Territories, Hong Kong SAR, China

[6]Institute of Systems Medicine and Health Sciences, Hong Kong Baptist University, Kowloon Tong, Hong Kong SAR, China

*Correspondence should be addressed to Pan Liao (panliao@hkbu.edu.hk) or Yudai Matsuda (ymatsuda@cityu.edu.hk).

Table of Contents

Supplementary Materials and Methods **S2–S10**

Supplementary Tables S1–S5 **S11–S16**

Supplementary Figures S1–S49 **S17–S62**

Supplementary References **S63–S64**

Supplementary Materials and Methods

General experimental procedures

Organic solvents were purchased from Anaqua (Hong Kong) Co. Ltd., and other chemicals were purchased from Wako Chemicals Ltd., Thermo Fisher Scientific, Sigma-Aldrich, or J&K Scientific Ltd., unless noted otherwise. Oligonucleotide primers (Table S3) were purchased from Beijing Genomics Institute. PCR was performed using a T100 Thermal Cycler (Bio-Rad Laboratories, Inc.) with Phanta Max Super-Fidelity DNA Polymerase (Vazyme Biotech Co., Ltd). Analytical HPLC was performed on a Dionex Ultimate 3000 UHPLC system (Thermo Scientific), using a Kinetex C18 column (5 μm, 100 Å, 4.6 x 150 mm; Phenomenex). Preparative HPLC was performed on a Waters 1525 Binary HPLC pump with a 2998 photodiode array detector (Waters Corporation). Flash chromatography was performed using an Isolera Spektra One flash purification system (Biotage). NMR spectra were obtained at 600 MHz (^{1}H)/150 MHz (^{13}C) with a Bruker Ascend Avance III HD spectrometer, and chemical shifts were recorded with reference to solvent signals (^{1}H NMR: $CDCl_3$ 7.26 ppm, DMSO-d_6 2.49 ppm; ^{13}C NMR: $CDCl_3$ 77.0 ppm, DMSO-d_6 39.5 ppm). HR-ESI-MS spectra were obtained with a SCIEX X500R Q-TOF mass spectrometer. Samples for LC-MS analysis were injected into a SCIEX ExionLC AD System with a SCIEX X500R Q-TOF mass spectrometer, using a Kinetex C18 column (5 μm, 100 Å, 4.6 x 150 mm; Phenomenex). Optical rotations were measured with a P-2000 Digital Polarimeter (JASCO Corporation). X-ray diffraction was measured with a Bruker D8 Venture Photon II diffractometer.

Microbial strains

Aspergillus pseudoterreus CBS 116.46, the parental strain of *A. pseudoterreus* ATCC 32359, and *Aspergillus fumigatus* CBS 144.89 were obtained from the Westerdijk Fungal Biodiversity Institute and used for cloning the *pts* genes and conducting the antifungal assay, respectively. *Aspergillus oryzae* NSARU1 (*niaD$^-$, sC$^-$, ΔargB, adeA$^-$, pyrG$^-$*)[1] was utilized as the fungal heterologous expression host. Standard DNA engineering was performed with *Escherichia coli* DH5α (Takara Bio Inc). *E. coli* Transetta (DE3) (TransGen Biotech Co., Ltd.) was used for the expression of the PtsG domains. *Saccharomyces cerevisiae* INVSc1 (Thermo Fisher Scientific) was used for the yeast-based assays.

Resistance gene-guided genome mining in fungi

1. Creation of a list of possible target proteins

All proteins encoded by *Aspergillus fumigatus* Af293 were subjected to a series of BLAST searches against the UniProtKB/Swiss-Prot database, as well as databases with proteins from selected filamentous fungi (*Alternaria alternata* SRC1lrK2f, *Fusarium oxysporum* Fo47, *Histoplasma capsulatum* G186AR, *Neurospora crassa* OR74A, *Penicillium rubens* Wisconsin 54-1255, and *Pyricularia oryzae* 70-15) and yeasts (*Candida albicans* SC5314, *Saccharomyces cerevisiae* S288C, and *Schizosaccharomyces pombe* 972h-). On the basis of the

conservation level in other organisms, a total of 1925 *A. fumigatus* proteins were selected to create a list of possible housekeeping proteins (Supplementary Data 1). During this process, each protein was labeled with its predicted function, and the protein sequence was revised where necessary.

Additionally, although not relevant to the present study, another list of proteins consisting of human proteins classified as "FDA approved drug targets" or "Disease related genes" in the Human Protein Atlas[2] was created for genome mining specifically targeting human proteins.

2. Development of a tool for resistance gene-guided genome mining in fungi

FunBGCeX,[3] which is a fungal genome mining tool developed in our previous study, was modified to achieve the following general procedure for resistance gene-guided genome mining. After detecting core biosynthetic genes and extracting proteins encoded by the genes in their flanking regions from a given fungal genome sequence, the resultant protein sequences are subjected to a BLASTp search against the database with the *A. fumigatus* proteins selected in the previous step using DIAMOND.[4] If there is a hit with >50% sequence identity and >50% coverage, the protein is considered a resistance protein candidate. Each candidate protein is then examined for duplication within the genome; if a homologue with >50% protein sequence identity is found, the protein is considered duplicated. Then, the sequence identity between the duplicated protein and its closest homologue in *A. fumigatus* is compared to that between the protein of interest and its homologue within the same genome. If the identity with the *A. fumigatus* homologue is greater by ≥12% than that with the duplicated protein, the protein is excluded as a resistance protein candidate. Additionally, proteins with ≥85% identity to the *A. fumigatus* homologue are also not considered a resistance protein. Resistance protein candidates are classified as biosynthetic proteins. The detection and extraction of biosynthetic gene clusters (BGCs) are performed as in the previous version of FunBGCeX, and only BGCs with a possible resistance protein are included in the final result.

BGC detection using the human protein database can also be performed as described above, but a requirement of >40% sequence identity and >40% coverage is used when identifying resistance protein candidates.

The latest version of FunBGCeX has been deposited at Zenodo under the DOI 10.5281/zenodo.17113445 and is also available at https://github.com/ydmatsd/funbgcex.

3. Resistance gene-guided genome mining in Pezizomycotina fungi and classification of the resultant biosynthetic gene clusters

A total of 2544 Pezizomycotina reference genomes were downloaded from the NCBI database in November 2023 (Supplementary Data 2). Unannotated genome assembly data were processed by AUGUSTUS[5] and NCBI Datasets[6] to generate annotated genome files in the GenBank format. The 2,544 genomes were then analyzed by FunBGCeX (v1.0.0) using the `-m sre` option for resistance gene-guided genome mining, yielding 7,425 BGCs.

The extracted BGCs were subsequently analyzed by BiG-SCAPE[7] (version 2.0.0b8) with a cutoff value of 1.0 (`--gcf-cutoffs 1`). Each gene cluster family was further divided into smaller families with BGCs sharing the same core biosynthetic proteins and possible resistance proteins. At this point, single-genus families were removed to facilitate the identification of BGCs with genuine resistance proteins. Consequently, 2,901 BGCs were classified into 413 gene cluster families (Supplementary Data 3).

Metabolite analysis of *Aspergillus pseudoterreus*

A. pseudoterreus CBS 116.46 was cultivated on a potato dextrose agar plate at 30 °C for seven days. A small piece of fungal mycelia and agar was cut from the plate, soaked in ethyl acetate, and extracted using an ultrasonic bath. The ethyl acetate layer was transferred to a new tube, and the solvent was removed using nitrogen gas flow. The residue was dissolved in methanol and analyzed by LC–MS, with a solvent system of 20 mM formic acid (solvent A) and acetonitrile containing 20 mM formic acid (solvent B), at a flow rate of 1 mL/min and a column temperature of 40 °C. Separation was performed using a linear gradient from 10:90 (solvent B/solvent A) to 100:0 for 10 min, 100:0 for the following 3 min, and a linear gradient from 100:0 to 10:90 within the following 2.0 min, and then 10:90 for 2.5 min of equilibrium.

Construction of fungal transformation plasmids

To construct fungal expression plasmids for *A. oryzae*, each gene in the *pts* cluster was first amplified from the genomic DNA of *A. pseudoterreus* CBS 116.46 with the primers described in Table S3 and Table S4. The *ptsA* gene was then introduced into the *Sma*I-digested pPyrG-HR[1] vector, using a ClonExpress Ultra One Step Cloning Kit (Vazyme Biotech Co., Ltd). Meanwhile, the other amplified DNA fragments were first ligated to the *Sma*I-digested pTAex3-HR vector,[8] and subsequently, a DNA fragment with the *amyB* promoter (*PamyB*) and the *amyB* terminator (*TamyB*) was amplified from the pTAex3-based plasmid and further introduced into the already constructed single gene-containing or another vector, pAdeA-HR.[9] Detailed methods for the construction of the plasmids used in this study are summarized in Table S4.

Fungal transformation

Transformation of *A. oryzae* NSARU1 was performed by the previously reported protoplast–polyethylene glycol method[10] coupled with CRISPR-Cas9-guided homologous recombination.[1, 8-9] The transformants created in this study and the plasmids used for the transformation are provided in Table S5.

HPLC analysis of each product from *Aspergillus oryzae* transformants and purified compounds

To analyze the metabolites from each *A. oryzae* transformant, the transformants were cultivated on a DPY agar plate [2% dextrin, 1% hipolypepton (Nihon Pharmaceutical Co., Ltd.), 0.5% yeast extract, 0.5% KH_2PO_4, 0.05%

$MgSO_4 \cdot 7H_2O$, and 1.5% agar] for seven days at 30 °C. A small piece of fungal mycelia and agar was cut from the plate, soaked in ethyl acetate, and extracted using an ultrasonic bath. The ethyl acetate layer was transferred to a new tube, and the solvent was removed under nitrogen gas flow. The residue was dissolved in methanol and analyzed by HPLC, with a solvent system of 20 mM formic acid (solvent A) and acetonitrile containing 20 mM formic acid (solvent B), at a flow rate of 1 mL/min and a column temperature of 40 °C. Separation was performed using a linear gradient from 10:90 (solvent B/solvent A) to 100:0 for 10 min, 100:0 for the following 3 min, and a linear gradient from 100:0 to 10:90 within the following 2 min, and then 10:90 for 2.5 min of equilibrium. Purified compounds were analyzed using the same analytical method.

Isolation of each metabolite

To isolate each metabolite, *A. oryzae* transformants were cultivated on 100 DPY agar plates (*ca.* 2 L) for seven days at 30 °C. The fungal cultures, along with agar medium, were crushed into small pieces, soaked in ethyl acetate, and extracted three times using an ultrasonic bath. After filtration, ethyl acetate was removed in vacuo. The obtained crude extract was fractionated by flash chromatography using an Sfär C18 Duo column (30 μm, 50 g; Biotage) and further purified by preparative HPLC using an XBridge BEH C18 OBD Prep Column (100 Å, 5 μm, 19 i.d. x 250 mm; Waters Corporation). Purification methods for each compound are described in detail below.

Purification conditions for pterrespiramide A (**1**) and pterrespiramide B (**2**):

The extract of *A. oryzae*/*ptsACEFG* (0.91 g) was subjected to flash chromatography and eluted stepwise using a water:acetonitrile gradient (100:0 to 0:100). Fractions that contained pterrespiramide A (**1**) and pterrespiramide B (**2**) were purified by reverse-phase preparative HPLC (60% aqueous acetonitrile, 10 mL/min) to yield 25.7 mg of **1** and 9.1 mg of **2**.

Purification conditions for pterramide A (**3**), pterrespiramide C (**4**), and pterrespiramide D (**5**):

The extract of *A. oryzae*/*ptsACEF* (0.71 g) was subjected to flash chromatography and eluted stepwise using a water:acetonitrile gradient (100:0 to 0:100). Fractions that contained pterramide A (**3**) were then purified by reverse-phase preparative HPLC (100% aqueous acetonitrile supplemented with 0.05% TFA, 10.0 mL/min) to yield 80.8 mg of **3**. Fractions that contained pterrespiramide C (**4**) and pterrespiramide D (**5**) were purified by reverse-phase preparative HPLC (55% aqueous acetonitrile supplemented with 0.05% TFA, 10.0 mL/min) to yield 18.9 mg of **4** and 48.7 mg of **5**.

X-ray crystallographic analysis

A single crystal of **1** was grown in acetonitrile/water (2:1, v/v) by evaporating at room temperature. Single crystal X-ray diffraction measurement was performed on a Bruker D8 Venture diffractometer using Cu Kα

radiation at 173 K. The data collection was performed with Bruker Instrument Service v8.5.0.27, and cell refinement and data reduction were carried out using the SAINT program. The structure of **1** was solved by the direct method with the SHELXT program and refined using the SHELXL program. All non-hydrogen atoms were refined anisotropically, whereas hydrogen atoms were placed by geometrical calculations.

Advanced Marfey's method

Compounds **1** and **3** were hydrolyzed in 2 N HCl at 115 °C for 1 hour, followed by cooling on ice for 3 minutes. The solution was then dried under nitrogen gas flow and dissolved in 200 μL of 1 N $NaHCO_3$. Subsequently, the hydrolysate was derivatized with 100 μL of 1% acetone solution of 1-fluoro-2,4-dinitrophenyl-5-L-leucinamide (L-FDLA) at 80 °C for 3 minutes. D- and L-5-hydroxynorvaline and D- and L-glutamic acid standards were derivatized under the same conditions. All samples were analyzed with the aforementioned LC–MS conditions used for analyzing the metabolites from *A. pseudoterreus*.

Expression and purification of A and T–R domains of PtsG

To obtain the intron-free DNA fragments for the A and T–R domains of PtsG, the predicted exons were amplified from the pTAex3-HR-ptsG plasmid constructed for fungal transformation, using the primers described in Table S3. Each amplified fragment was then introduced into the pET-28a(+) vector (Novagen), using a ClonExpress Ultra One Step Cloning Kit (Table S4). The methods used for the *E. coli* transformation and gene expression are the same as described for ScALS. After the completion of cultivation, the *E. coli* cells were collected by centrifugation and resuspended in lysis buffer (50 mM Tris-HCl, pH 7.5, 150 mM NaCl, 5 mM imidazole, and 5% glycerol). After cell lysis by sonication and subsequent centrifugation, the supernatant was loaded on a Ni-NTA affinity column, which was then washed with 30 CV of wash buffer (50 mM Tris-HCl, pH 7.5, 150 mM NaCl, 10 mM imidazole, and 5% glycerol). The His-tagged proteins were eluted from the column using 5 CV of elution buffer (50 mM Tris-HCl, pH 7.5, 150 mM NaCl, 300 mM imidazole, and 5% glycerol). The protein solution was ultrafiltrated and concentrated using a Vivaspin Turbo 15 with a 10,000 MWCO PES membrane (Sartorius) with imidazole-free buffer (50 mM Tris-HCl, pH 7.5, 150 mM NaCl, 5% glycerol). The purity of each purified protein was examined by SDS-PAGE (Figure S14), and the protein concentrations were determined with a NanoDrop OneC spectrophotometer (Thermo Scientific).

Enzymatic reaction assay of A and T–R domains of PtsG

In vitro enzymatic reactions were performed in a 100 μL reaction mixture containing 50 mM Tris-HCl (pH 7.5), 1 mM of each substrate (**3**, **4**, or **5**), 4 mM ATP, 2 mM NADPH, 8 mM $MgCl_2$, 1 mM coenzyme A trilithium salt, 5 μM 4′-phosphopantetheinyl transferase Sfp, 7.5 μM A domain of PtsG, and 7.5 μM T–R didomain of PtsG at 30 °C for five hours. The reaction was terminated by extracting the reaction mixture with ethyl acetate.

The organic layer was dried and dissolved in 50 μL of methanol. The resultant solution was analysed with LC–MS using the method described above for analyzing the metabolites from *A. pseudoterreus*.

Construction of *Saccharomyces cerevisiae* transformants

To obtain the intron-free *ptsB* gene, total RNA was extracted from *A. oryzae* transformant carrying *ptsABCDEFG* genes, which has been statically cultivated in DPY medium for 7 days at 30 °C, using a GeneJET plant RNA Purification Kit (Thermo Scientific). Complementary DNA (cDNA) synthesis was then performed with a HiScript III 1st Strand cDNA synthesis Kit (+ gDNA wiper) (Vazyme Biotech). The acetolactate synthase gene from *S. cerevisiae* (*ilv2*) and the intron-free *ptsB* gene were amplified by PCR and ligated to a *Bln*I-digested pAT426 vector[11] [National BioResource Project (NBRP) yeast, Japan] (Table S4). Yeast transformation was performed using a Frozen-EZ Yeast Transformation II Kit (Zymo Research) according to the manufacturer's instructions. Transformants were subsequently selected on uracil dropout synthetic complete medium (SC–U) agar plate [6.7 g/L Yeast Nitrogen Base without amino acids (Sigma), 1.92 g/L Yeast Synthetic Drop-out Medium supplements without uracil (Sigma), 20 g/L glucose, 20 g/L agar].

Growth inhibition assay of *Saccharomyces cerevisiae*

Successful *S. cerevisiae* transformants were grown in isoleucine, leucine, and valine dropout minimal medium [20 g/L glucose, 6.7 g/L Yeast Nitrogen Base without amino acids (Sigma), 18 mg/L adenine, 76 mg/L arginine, 76 mg/L asparagine, 76 mg/L aspartic acid, 76 mg/L glutamic acid, 76 mg/L histidine, 76 mg/L lysine, 76 mg/L methionine, 76 mg/L phenylalanine, 76 mg/L serine, 76 mg/L threonine, 76 mg/L tryptophan, 76 mg/L tyrosine] to test for growth inhibition in the presence of **1**, **2**, or sulfometuron methyl. Initially, each *S. cerevisiae* strain was cultivated at 30 °C overnight. The cell number was then adjusted to 1×10^5 with the medium. After that, each strain was treated with different concentrations of **1**, **2**, or sulfometuron methyl for 48 hours. The growth inhibition percentage of each strain was calculated by dividing the cell density (OD_{600}) of the treated strain by the OD_{600} of the negative control strain.

Expression and purification of ScALS

The acetolactate synthase gene (*ilv2*) lacking the sequence for the mitochondrial transit peptide was amplified from the genomic DNA of *S. cerevisiae* INVSc1 using the primers provided in Table S3. The mitochondrial transit peptide was removed according to a previous study (PDB: 6U9D).[12] The amplified truncated *ilv2* gene was then introduced into the pET-28a(+) vector (Novagen), using a ClonExpress Ultra One Step Cloning Kit (Table S4). For the expression and purification of the truncated acetolactate synthase from *S. cerevisiae* (ScALS), *E. coli* Transetta (DE3) was transformed using the above-constructed pET-28a(+)-based plasmid. The *E. coli* transformant was first cultivated using shaking at 37 °C/200 rpm in an LB medium containing 50 mg/L kanamycin sulfate and 34 mg/L chloramphenicol. When the cultures reached an OD_{600} of 0.55, 0.05 mM IPTG

was added to induce gene expression, followed by further incubation at 16 °C/200 rpm for 20 h. The cells were then collected by centrifugation and resuspended in lysis buffer (50 mM potassium phosphate buffer, pH 7.0, 10 mM $MgCl_2$, 150 mM NaCl, 5 mM imidazole, 10% glycerol, and 10 μM FAD). After cell lysis by sonication and subsequent centrifugation, the supernatant was loaded on a Ni-NTA affinity column, which was then washed with 30 column volumes (CV) of wash buffer (50 mM potassium phosphate buffer, pH 7.0, 10 mM $MgCl_2$, 150 mM NaCl, 10 mM imidazole, 10% glycerol, and 10 μM FAD). The His-tagged protein was eluted from the column using 5 CV of elution buffer (50 mM potassium phosphate buffer, pH 7.0, 10 mM $MgCl_2$, 150 mM NaCl, 300 mM imidazole, 10% glycerol, and 10 μM FAD). The protein solution was ultrafiltrated and concentrated using a Vivaspin Turbo 15 with a 10,000 MWCO PES membrane (Sartorius) with imidazole-free buffer (50 mM potassium phosphate buffer, pH 7.0, 10 mM $MgCl_2$, 20% glycerol, 10 μM FAD, 1 mM thiamin diphosphate, and 2 mM β-mercaptoethanol). The purity of each purified protein was examined by sodium dodecyl sulfate-polyacrylamide gel electrophoresis (SDS-PAGE) (Figure S14), and the protein concentrations were determined with a NanoDrop OneC spectrophotometer (Thermo Scientific).

Enzymatic reaction assay of ScALS and inhibition studies

The in vitro enzymatic reaction of ScALS was performed in 100 μL of reaction mixture containing 50 mM potassium phosphate buffer (pH 7.0), 10 mM pyruvate, 10 mM $MgCl_2$, 10 μM FAD, 1 mM thiamin diphosphate, 2 mM β-mercaptoethanol, and 1 μM ScALS, in the presence of varied concentrations of **1** or **2**, at 30 °C for one hour. The reaction was quenched by adding 10% H_2SO_4 to a final concentration of 1% (v/v), and the mixture was then heated to 60 °C for 15 minutes. The quantification of acetoin, converted from acetolactate in the previous step, was performed by adding 15 μL 6 M NaOH, 50 μL 0.5 % creatine (in water), and 50 μL 4% α-naphthol (in 2.5 M NaOH), followed by heating at 60 °C for 15 minutes. The absorbance of the resultant mixture at 530 nm was measured using a plate reader, and the relative activity was calculated by comparing with the DMSO control.

Assay for evaluating antifungal activity towards *Aspergillus fumigatus*

The antifungal assay was conducted with a microdilution assay according to established protocol with slight modifications.[13] *Aspergillus fumigatus* CBS 144.89 was subcultured on a PDA plate and incubated at 30 °C for seven days. The spores were harvested by overlaying and scraping the agar plate with 3 mL sterile 20% glycerol. The spore suspension was then diluted with Mueller–Hinton Broth (Sigma-Aldrich) to an OD_{530} of 0.09–0.13. After that, 0.1 mL of the diluted suspension was added to 5 mL Mueller–Hinton Broth to yield the final inoculum. In each well of a 96-well plate, 0.1 mL of the final inoculum was added along with various concentrations of **1** or **2**. The minimum inhibitory concentration (MIC) of **1** or **2** was defined as the lowest concentration at which no visible fungal growth was observed.

Evaluation of the effect of pterrespiramide A on primary root growth of *Arabidopsis thaliana*

To explore whether pterrespiramide A (**1**) shows herbicidal activity, experiments were performed on *Arabidopsis thaliana* as previously described[14] with slight modifications. Compound **1** was initially dissolved in 100% methanol (v/v) to create a stock solution. This stock was then further diluted with DMSO to achieve a working concentration of 5 mM. Subsequently, the 5 mM solution was diluted with Murashige and Skoog (MS) medium to obtain the final desired concentrations of 2 μM, 5 μM, and 10 μM. Wild-type *Arabidopsis thaliana* seeds, provided by Prof. Yang Bi (Hong Kong Baptist University, Hong Kong, China), were surface sterilized with 2% (v/v) sodium hypochlorite (NaClO) for 5 min, then washed three times with sterile distilled water and sown on plates containing MS medium with 2% (w/v) sucrose and 0.8% (w/v) agar. After 3 days of stratification at 4 °C in the dark, the plates were transferred to a growth chamber and placed vertically. The growth conditions in the growth chamber were set at 22 °C with 16-hour light and 8-hour dark cycles, 100 μmol photons m^{-2} s^{-1} light intensity, and 60% relative humidity. Five-day-old *Arabidopsis* seedlings with primary root length around 1 cm on MS medium plates were transferred to new square plates (10 cm×10 cm) containing fresh MS medium with or without DMSO, and fresh MS medium containing different concentrations of **1** (2 μM, 5 μM and 10 μM, respectively). For each concentration of compound **1** treatment or DMSO control, at least 15 seedlings were grown on each plate, and a total of three Petri dishes were used for each concentration. Seedlings were photographed daily for five days, and their root lengths were measured manually with a ruler and confirmed using ImageJ software.

Analytical data

*Pterrespiramide A (**1**).* Colorless crystal; $[\alpha]^{20}_{D}$ +156.6 (*c* 1.0, $CHCl_3$); for NMR data, see Figure S15 to Figure S21; HRMS (ESI) *m/z*: $[M + H]^+$ Calcd for $C_{23}H_{32}NO_4$ 386.2326; Found 386.2312.

*Pterrespiramide B (**2**).* White powder; $[\alpha]^{20}_{D}$ –0.2 (*c* 1.0, $CHCl_3$); for NMR data, see Figure S22 to Figure S28; HRMS (ESI) *m/z*: $[M + H]^+$ Calcd for $C_{23}H_{32}NO_4$ 386.2326; Found 386.2311.

*Pterramide A (**3**).* Yellowish oil; $[\alpha]^{19}_{D}$ –37.7 (*c* 1.0, $CHCl_3$); for NMR data, see Figure S29 to Figure S35; HRMS (ESI) *m/z*: $[M + H]^+$ Calcd for $C_{23}H_{33}NO_5$ 404.2431; Found 404.2426.

*Pterrespiramide C (**4**).* White powder; $[\alpha]^{19}_{D}$ +81.2 (*c* 0.7, acetone); for NMR data, see Figure S36 to Figure S42; HRMS (ESI) *m/z*: $[M + H]^+$ Calcd for $C_{23}H_{29}NO_5$ 400.2118; Found 400.2009.

*Pterrespiramide D (**5**).* White powder; $[\alpha]^{19}_{D}$ +25.8 (*c* 1.0, acetone); for NMR data, see Figure S43 to Figure S49; HRMS (ESI) *m/z*: $[M + H]^+$ Calcd for $C_{23}H_{29}NO_5$ 400.2118; Found 400.2114.

*Crystallographic data for **1**.* $C_{23}H_{31}NO_4$, M = 385.49, a = 7.6703(2) Å, b = 9.8254(2) Å, c = 28.0736(7) Å, α = 90°, β = 90°, γ = 90°, V = 2115.73(9) Å^3, T = 173(2) K, space group $P2_12_12_1$, Z = 4, μ(Cu Kα) = 0.657 mm^{-1}, 26 154 reflections measured, 3862 independent reflections (R_{int} = 0.0726). The final R_1 values were 0.0449 ($I > 2\sigma(I)$). The final $wR(F^2)$ values were 0.1230 ($I > 2\sigma(I)$). The final R_1 values were 0.0463 (all data). The final $wR(F^2)$ values were 0.1248 (all data). The goodness of fit on F^2 was 1.051. Flack parameter 0.14(9). The crystallographic information file (CIF) for this crystal structure was submitted to The Cambridge Crystallographic Data Centre (CCDC) under reference number 2487927.

Table S1. Annotation of each gene in the *pts* cluster from *Aspergillus pseudoterreus* ATCC 32359 (GenBank accession: PIJX01000031.1; nucleotide positions 256,596–282,718).

The *pts* cluster from *Aspergillus pseudoterreus* ATCC 32359

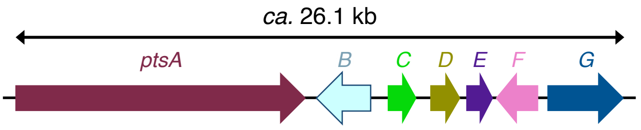

Gene	Amino acids (base pairs)	Protein homologue (origin)	Similarity/Identity (%)	Proposed function
ptsA	3967 (12418)	TasS (*Hapsidospora irregularis*)	69/53	polyketide synthase–nonribosomal peptide synthetase (KS-AT-DH-MT-KR-ACP-C-A-T-DKC)
ptsB	688 (2332)	CfoL (*Aspergillus candidus*)	82/71	acetolactate synthase
ptsC	395 (1188)	Tas3 (*Hapsidospora irregularis*)	72/58	Diels-Alderase
ptsD	400 (1203)	TasR (*Hapsidospora irregularis*)	48/36	transcription factor
ptsE	363 (1092)	CghC (*Chaetomium globosum*)	77/62	*trans*-acting enoylreductase
ptsF	532 (1769)	HepE (*Aspergillus oryzae*)	67/46	cytochrome P450 monooxygenase
ptsG	1058 (3229)	FSL6 (*Fusarium graminearum*)	59/40	carboxylic acid reductase (A-T-R)

Table S2. Sequences of the *pts* genes and their products. Highlighted in magenta are the regions predicted for an intron in this study.

Gene	DNA sequence	Protein sequence
ptsA	atggcggcatctcaacaggagcccattgccattattggaagtgcttgtcgattcccaggaagctcagatactccatcaaagctatgggagcttcttcgatcgccacgcga<br>ccttctaaagcgtgtgccagcggaacgttttgatgcatccgcatattatcatcccgactctagccatcacggtaccacggattgtcaagagtcgtactttcttgacgaggatg<br>tgtctcaatttgacaacgccttcttcaatattcagcccgctgaggctgaggctattgatcctcagcagcgtctcctcatggagacggtctatgactccctgtaagttctaaaat<br>atccctttatgagctaggtcaccgccagtagtggttactgattcatagcttgtaggtgtgcttctggccagactatcgagggcctacggggctcatccactggggtatatgtc<br>ggtgtgatgtgtgacgactggtcccaaatttccaatcgcgactgggacttgatccacacatacgccgctaccggtaccagccgctgtatcatttcaaaccggatttcctactt<br>ctttgattggcatggccctagtatgaccatcgatactgcttgctcatcatcactcgtttgtgtccatcaggctgtgcaagcccttcggagcggcgagtcccgagtggctattgc<br>cgcaggtgctaatctgatactgagtcctggtaagtcaagaatgaacggttgcccacgaacacggctaacattggtcgaattaaggtatgtatattgcggagagcaaact<br>caacatgctgtctcccacggggaggagtcggatgtgggatgccaatgcagacggatacgctcgaggtgaaggccttgcagccgtggtattgaagcctctcagtgctgc<br>aattgaggatggcgaccatgtcgagtgtatcattcgtggcactggtatcaatcaggatggtcgcacagctggcctgaccatgccgagtaacatcgcacaggccaacctc<br>attcggacaacttatgcccgcgctgggttagacatcaatgatcccaaggaccgccctcagtttttccatgcccatggtacgggaacacctgctggggatccgcaggaatc<br>agaggccatctcacgtgcgtttttcgggaacagcaatgtgacggacacactatacgtgggttctatcaagactataattgggtgcgtacattccattcttctttcccttcatcgg<br>acgcgaaacagcacttctggcttactatagtctgagccatactccatgaggccttacccgacaaatcagggaatttagttactaactatggagatgtacagtcataccga<br>gggcacagcaggacttgcgagcctaattggtacctctcaagctctacagcatggaatgatacctcctaacatgcattttaactcgctgagtcctcgagttgcaccttttttata<br>ctcatcttgaggtccccaccgagcttcggccctggcccagtccagtatccggccaaccgcgccgtgcgagtatcaacagtttcggtgagatcagagagccttctagattg<br>gtcaagtgacagtatttactaatccatgcccaggtttcggtggcaccaatgcgcatgctattcttgagtcatatgaagttgaaacgtccaaatcagctgtcgtgcctacattc<br>actccccttgtcatctcagcggcttccaagatctcactgcgtgccatgctctcggaacttagcagcttcctcaagaacaaacctgatacaaacctacgcgacctcgcgtat<br>acgctccacactcgccgctcgaccctacaatttcgacatgtgatcactggcatgaatgttcaggagatcattacgaggattgacaacattgttgctgaagatgactccagtt<br>tgaacacccgttatttcagcgtgccgaatccaaagattctcggcattttcactggtcagggtgcccaatggccacgtatgggagctcagctcttcgaggcttcaccttttgttt<br>ctgagcgtctcgcagagctcgatcgtgctttatgcagtcttcccgaagcggaccgccctcagtggttgcttaaggaccagctgctggcggaggctaaggtatcgaaaatc<br>tcgcaggccgccgtttcacaacctctctgcactgctatacagattgtcctcgtcgatatgctgaagttggccgggataaatctgcacgctgttgtcggtcactcgtctggtga<br>aattggcgctgcatatgcggccggattcctctcggccaccgacgcaatccggattgcttactatcgtggcctctacgccaagcttgccgaatctccaaatggcaccaaag<br>gtgctatgatagctgtcggtacatcgtttgaggatgctgtcgaattttgtgagctcgaggaatttgaagaccgactccaagtggccgctcgcaactcctccactagcgttact<br>ctctccggagacgaggatgccattcttgaggctcttgagatcttccaggatgagggcaagtttgctcgtcagcttaaggtggatacagcctatcactcgcagcacatgctg<br>ccttgtgcggcatcatatcttgaagctatgaataggtgcaatataacagtgcgcgacggcagtggtcctatttggttttctagtgtctctggtggccaagtcatgacgaaaga<br>aatggttcgatctcagtactgggtagagaatatgactcaaactgttctattctcgcctgctgtcactgccgctgtcactgaagctagtccatttgacctcgcactcgagattgg<br>acctcacccggccctgaagagtgtggctctcgacaccatcgaagaatcaacgggcagccgagtcccatattctggtaccctttctcgaggcaagaatgatattctcgag<br>ctctcatccgcacttggatttgcctggacccatctcggtcctaattctgtcaactttgacgcgtttgagaaggccgcatcgggtaatcagcgcccaaaatcacttgtggcgga<br>actgcccaagtatccgtttgaccattcgcgctcgttcatgatgctgactcgcttctctggcggccacgcgaacatccactcaccacctcatccactgcttggccgacgctgtt<br>ttgaacatgaaaccacccaagaagtgcaatggcgtaacttcttgggtccgaaggaggtcagctggctgaacggccacaagcttcaaggccagacagttttcccggcta	MAASQQEPIAIIGSACRFPGSSDTPSKLWELLRS<br>PRDLLKRVPAERFDASAYYHPDSSHHGTTDCQE<br>SYFLDEDVSQFDNAFFNIQPAEAEAIDPQQRLLM<br>ETVYDSLCASGQTIEGLRGSSTGVYVGVMCDDW<br>SQISNRDWDLIHTYAATGTSRCIISNRISYFFDWH<br>GPSMTIDTACSSSLVCVHQAVQALRSGESRVAIA<br>AGANLILSPGMYIAESKLNMLSPTGRSRMWDAN<br>ADGYARGEGLAAVVLKPLSAAIEDGDHVECIIRGT<br>GINQDGRTAGLTMPSNIAQANLIRTTYARAGLDIN<br>DPKDRPQFFHAHGTGTPAGDPQESEAISRAFFG<br>NSNVTDTLYVGSIKTIIGHTEGTAGLASLIGTSQAL<br>QHGMIPPNMHFNSLSPRVAPFYTHLEVPTELRP<br>WPSPVSGQPRRASINSFGFGGTNAHAILESYEVE<br>TSKSAVVPTFTPLVISAASKISLRAMLSELSSFLKN<br>KPDTNLRDLAYTLHTRRSTLQFRHVITGMNVQEII<br>TRIDNIVAEDDSSLNTRYFSVPNPKILGIFTGQGA<br>QWPRMGAQLFEASPFVSERLAELDRALCSLPEA<br>DRPQWLLKDQLLAEAKVSKISQAAVSQPLCTAIQI<br>VLVDMLKLAGINLHAVVGHSSGEIGAAYAAGFLS<br>ATDAIRIAYYRGLYAKLAESPNGTKGAMIAVGTSF<br>EDAVEFCELEEFEDRLQVAARNSSTSVTLSGDE<br>DAILEALEIFQDEGKFARQLKVDTAYHSQHMLPC<br>AASYLEAMNRCNITVRDGSGPIWFSSVSGGQVM<br>TKEMVRSQYWVENMTQTVLFSPAVTAAVTEASP<br>FDLALEIGPHPALKSVALDTIEESTGSRVPYSGTL<br>SRGKNDILELSSALGFAWTHLGPNSVNFDAFEKA<br>ASGNQRPKSLVAELPKYPFDHSRSFMMLTRFSG<br>GHANIHSPPHPLLGRRCFEHETTQEVQWRNFLG<br>PKEVSWLNGHKLQGQTVFPATGYIAMAVEAMAV

	cgggatatattgccatggcagtcgaggcaatggccgtacttgccggcgacaagccaatcggactcatcagcttggatgaattcgttatttcccgtgccgttacattcaatga	LAGDKPIGLISLDEFVISRAVTFNEGEIGVESLVTL
	gggcgagattggtgtggagtctttggtgaccctaaacataatccgttcctcggataatgagctctgtgcaaacttcacctgttgtaccggcttacccttcgacaactcatcctc	NIIRSSDNELCANFTCCTGLPFDNSSSMILNSKAT
	gatgatcttaaattccaaagccactgtcactctggccttccatgagcctgcccctgataccttaccaagtgtgagagcggaagacgccagcttggctgacgtcgatgtcg	VTLAFHEPAPDTLPSVRAEDASLADVDVDIERFY
	acattgagcgcttctatacccagctcacacgtctcggttataattacagcgggtcattcaggtctgtcaagagcattcgacgccggaaggactttgcgactggcattattga	TQLTRLGYNYSGSFRSVKSIRRRKDFATGIIEDES
	ggacgaatcagagaacaattgggaagaccagctcatagtacacccttgctggctagatactgccattcagaccgggtttgctgcttactgctacccccaggatgagcgt	ENNWEDQLIVHPCWLDTAIQTGFAAYCYPQDER
	ctttggacgcttcacgtcccgacggctagcccgtctattgtgatcaacccctactttacgcgcctgggagctgggaaacagcgagttctgccttaccagtccgttgtccgca	LWTLHVPTASPSIVINPYFTRLGAGKQRVLPYQS
	attcgcgaaaggctcagatgagttgcgatgtcgacgttatggctggagatgacggttcgcacacatttgttcagattgaatctcttcaattaaggcccttttctccggctaccg	VVRNSRKAQMSCDVDVMAGDDGSHTFVQIESL
	ctgcttcggatgctgtcctgttctcccattttgactacaaactcgctggacctagtggggtagccgctgttggcactgatcaggtcctgccgcccgaaaaggccggcgttgta	QLRPFSPATAASDAVLFSHFDYKLAGPSGVAAV
	cttgcaaccgagcgcatgggcttcttctatctgcgccgcctggtggaacagattacaccagaggagaagaataacaccctgccacactaccaacggctcttggactgg	GTDQVLPPEKAGVVLATERMGFFYLRRLVEQITP
	gccgaatacgctgtaaacgtcgtctcaagcggcagacatccgaatgtgccacaagaggcagtgcacgacacgcatgagtctatcaaagccattatcaaaaggcatta	EEKNNTLPHYQRLLDWAEYAVNVVSSGRHPNVP
	cagccatacagacgtgcgactggttgaagctgtgggtgagaatatcatcagtgaaattcgcaagcaaggaagcattcttgagcatatgatgaaggatggtgttctcgata	QEAVHDTHESIKAIIKRHYSHTDVRLVEAVGENIIS
	tgttttacgaagatgctgtgggacttgacactgcaaatatctggatcggtcgcatggtcgctcagattgctcatcgctacccgcgcatgcaaatcctggagatcggagccg	EIRKQGSILEHMMKDGVLDMFYEDAVGLDTANIW
	gaactggcgggagtactcgttcaatcttgccagagctgggttctgccttctcttcttacacctatacggatatctcggcgggattctttgagcgtgcccaggaccgtttccaag	IGRMVAQIAHRYPRMQILEIGAGTGGSTRSILPEL
	aatatggcgaccgcatggtatttaagacgtttgacatggagagatcgcccatcgaacaaggatacactgaaggatcctatgacatcgtccttgcgtcgaatgtccttcac	GSAFSSYTYTDISAGFFERAQDRFQEYGDRMVF
	gccacaggaaagctggacgagatgatggccaatgtgagacagctgttgagaccgggtggctatctgattgtacttgagattataaccaatgagttcctcggtatcggtac	KTFDMERSPIEQGYTEGSYDIVLASNVLHATGKL
	tgccatgggcggcctgcctgggtggtgggcaggtgccgcgcaggatgagcgacgtcgggcgggccctaccctgacactgcagcagtgggacaagttgatgcgcgat	DEMMANVRQLLRPGGYLIVLEIITNEFLGIGTAMG
	catggattcggtggcattgaaacggccactccgtcctctcacaagcttcacccttactcggtctttgcatctcaagctgttgacgatcgtgttgccgcattgcgcaatcctcttt	GLPGWWAGAAQDERRRAGPTLTLQQWDKLMR
	ctacaacgtctctgggttctacatccaacctagttgtcgttgggggacgtatgccggcgactcgtgagctagtcgagcaggtttgcgcactcattgagcagcaatactcca	DHGFGGIETATPSSHKLHPYSVFASQAVDDRVA
	agataactcgcctggattgtttggaggacctcaacgaacaaaatctaggtctttcgagctccgtcctttccctcacagatctggacgagccgttcttgcagatgcgtactgc	ALRNPLSTTSLGSTSNLVVVGGRMPATRELVEQ
	ctccaagcttgaggctctcagaaccctgtggcgacggggcggtagcatcttgtgggtgagccgtggatgtcgtgatgacaacccacactcttccatggtgcttgggctttc	VCALIEQQYSKITRLDCLEDLNEQNLGLSSSVLSL
	ccgagctatgaggttcgagtaccccagcataaatctccaaatgctcgatttcgatgttatcacatcccagtcttctcatatcctggctgagacacttatacgtctagaactcct	TDLDEPFLQMRTASKLEALRTLWRRGGSILWVS
	aggcaagtggcgcaaggatgcggatggccttaccaatgagttcctgtggtccttggagccggagctagtttatgaaaacaaccagctcctcataccgcgcatgtatccg	RGCRDDNPHSSMVLGLSRAMRFEYPSINLQMLD
	cacaacgcggctaataaccgctacaacacgtatcgtcgtacaatacatgagacggtcgacccaaggaaccacactatcgccctggaaactaaaggacaatcctatg	FDVITSQSSHILAETLIRLELLGKWRKDADGLTNE
	agctttccagcgtgtcgcctcttcgcctgccagcattgaccctgtttgccgaggatcgcatgtcaattcgagtgtctcattcagttctgcagatggtcaaagttcagaatggag	FLWSLEPELVYENNQLLIPRMYPHNAANNRYNTY
	gctgcttcatgctgtgtgcaggaaccgacatcgccactggccggcagcttattgctctatctgacaaggccgaaactcctgcacccacacggggacagtgggtagtccc	RRTIHETVDPRNHTIALETKGQSYELSSVSPLRLP
	agtatcacaatcgacaaatctagctgaagccctcctttcggtcgccgcacacgccctcgcacaaagcatcatcgctgctgctccaaaaactggcactatcttcatacatg	ALTLFAEDRMSIRVSHSVLQMVKVQNGGCFMLC
	aagccgatcccattctgatggctgcagtgatccaggaggccaatctgtctggcgtgaaggtgtcattctcttcgtccttcaaggataggaggatcaaaaactgcgccttcgt	AGTDIATGRQLIALSDKAETPAPTRGQWVVPVSQ
	acaccgcaatttgcctacgcgcttgatcaagaagcagctacctcacgacatttcctactttgtcaacctagcacaggctgtcggggcccaagaggtcggtcagaccattg	STNLAEALLSVAAHALAQSIIAAAPKTGTIFIHEAD
	ctaactgcctcacgccacacacgcggatcgccagtgcagttgactttgttgggaatgaaacgcttgtctaccctggcgcttcggctgaggcgattgggttggccctagca	PILMAAVIQEANLSGVKVSFSSSFKDRRIKNCAFV
	acggcctggcagcgaagtcaaactaggaatgatgtttcatcatacgagatgtcgaatgtgtcattacaggacatctccaaccattcaattattgatgaaagattacgcgta	HRNLPTRLIKKQLPHDISYFVNLAQAVGAQEVGQ
	gttcactgggatgtcccatccgtcgaaatttctctgcagcccattgataccggtaacatcttccgcgccgatgggacatatttcctcgtgggtctctcaggtcaacttggtcaat	TIANCLTPHTRIASAVDFVGNETLVYPGASAEAIG
	cgctctgtcactggatggtgactcacggagcccgcaatgtcgttctcacaagccgaaatcccaagattcgccatgggtttgtgaagatgatagaggaatcaggcgctact	LALATAWQRSQTRNDVSSYEMSNVSLQDISNHSI
	gtcaagttcatgtccctgtaagttgccctgtatcagttttgatatgacctttgccgtcctatcagacgctcctgtgtctacgctaatcttacatttccagggatgtgacatgccgcg	IDERLRVVHWDVPSVEISLQPIDTGNIFRADGTYF
	aatcacttcattcctgctaccaagaaatatgtcggacgatgccaccggttatcggtgttgcaaatggtgctatgatccttgaagaccagctttttgacaatttaacatgggagt	LVGLSGQLGQSLCHWMVTHGARNVVLTSRNPKI
	cctttaaccgacagttagcccccaaggtcgacggcagcagattgctggacgaactttttttacacctccaagctcgatttcttcattctcttcacgtcattgggcaacatcatcg	RHGFVKMIEESGATVKFMSLDVTCRESLHSCYQ
	gaaactcaggtcaagccagctatgttgcagcaaaccagtacatggttgccttggccgcccagcgcaaaaagcgcggcgtggccgggtcggcaattgccatcagttcc	EICRTMPPVIGVANGAMILEDQLFDNLTWESFNR
	ctcctgggtattggctatgtggagcggtccgagatgtttgactccgatcacttcactagaatcggataccggaacatgtcagaacaggattatctgcagctttttgctgaagg	QLAPKVDGSRLLDELFYTSKLDFFILFTSLGNIIGN
	tatcatcgtcggcaagccgggcaatccagagaactcagagatcgctactggcatgagtcccacatatgacgatcagagcatccaggctcaattccgcagcgacccca	SGQASYVAANQYMVALAAQRKKRGVAGSAIAIS
	agtttgtccatttcatcatggagaaacctgacactcagaccatcaccagcaatactaccattgtacctgctcgcgtccgtcttgcggatgtgaagtctactacagaagcag	SLLGIGYVERSEMFDSDHFTRIGYRNMSEQDYLQ
	ctgcggtaataaaaggtaacatcacacccaatcatgaactatagccatcactaacgcgctttaaatagaatctttcattgcgcgcttgaagcgaattcttcaaatcccgca	LFAEGIIVGKPGNPENSEIATGMSPTYDDQSIQAQ
	agatgagtccgtaaatgagagagctacactggtggagcagggtgtcgattccatcatggctgttgaagtccgttcctggttcctgaaggagttggatgttgacatgcccgt	FRSDPKFVHFIMEKPDTQTITSNTTIVPARVRLAD
	gttgaagattcttagcgccagtgccaccattcaggagctggttgacgagtccatggacagacttcctccttcgatcattgacctcagcaaccttgagtctggtggtcctgtga	VKSTTEAAAVIKESFIARLKRILQIPQDESVNERAT
	ctgccgtacagactcttcctgcccgagtaaactctcctccaacgagcctaccatcggagactcaatctgggtcatcataccccgtatcctcggcgacagcttcacccgca	LVEQGVDSIMAVEVRSWFLKELDVDMPVLKILSA
	accactccgtctacgggttcaggtcttgatacacccgaagagttggaggaacccactaaagcagatgagtcgaaacaacattggcgcgagtctgtgatcaagaactcc	SATIQELVDESMDRLPPSIIDLSNLESGGPVTAVQ
	actgaagcgacccagccaatgtcgtttgggcaatcgaggttttggtttctaacccactacatcaaggacaccaaagcgttcaacataacctgcatgttcaagctcacggg	TLPARVNSPPTSLPSETQSGSSYPVSSATASPAT
	gccacttcgtattgatgccttggaaaaagcggtagaaaagctgggccagcggcatgaggctttgcgcaccaggtatttctggtcagacgatggcaagactccaatgca	TPSTGSGLDTPEELEEPTKADESKQHWRESVIKN
	gggtgtcctctcgggttcccttatgcagctggaagtgaaacatgttacttcagaagctgaggtgtctgaagagtataacgctatgcgcgatcacgagtgggatctgggtga	STEATQPMSFGQSRFWFLTHYIKDTKAFNITCMF
	ctgggtgaccgtcagaattcgcttactttcactttccaactcagtgcactatttgctcgttgggacacaccatatctcgctggatggtcacagtttcaatgtcctgttcctggatct	KLTGPLRIDALEKAVEKLGQRHEALRTRYFWSDD
	cgatgctctatattccaaccgcgctctgccggctctgcctgatgcgtcgcaatacagggcctttgcaagacaacagaggcagctttacgagagcggcaggatggattcc	GKTPMQGVLSGSLMQLEVKHVTSEAEVSEEYNA
	gcgatggaatactatcgtgccatcataccgcacgacttgaagcccataagcttgctcccatttgctaaatctcaagtccgccccgcacttgatcgctacagcacgcatga	MRDHEWDLGDWVTVRIRLLSLSNSVHYLLVGTH
	ggtccagtttcagatccagccaccactcgcctccaagctgaagcacctcgctcgtcggcatcgttccacgtcgttccatctttacctggctgccctccaagcgcttatcttcc	HISLDGHSFNVLFLDLDALYSNRALPALPDASQY
	acatgctcccagaaaccaatgagttcttcattggtattgctgacgccaataggctcgacaaagacttcatgaacagtgtcggtatgctctttaacctgcttcctctacgcttcg	RAFARQQRQLYESGRMDSAMEYYRAIIPHDLKPI
	acagaccggaacctagcgtcaagttcagcgacgtcatccagacagcgcgtactaaggcatatgctgcacttgaacattcgagtgtacctttcgacattctcctaaatgac	SLLPFAKSQVRPALDRYSTHEVQFQIQPPLASKL
	ctcaatgttccgcgttccagctccgctgcacccattttccaaatcctcgtggactaccggttggtagtccaggagcgcacgacttacgccggttgcaagttgagcgacgag	KHLARRHRSTSFHLYLAALQALIFHMLPETNEFFI
	caatggcatgctgctcgaatcggctacgacatgggcctagagataacggagaaccccacgggcgaatcgaaattgactctgaggatgcaggatgcactgtattctca	GIADANRLDKDFMNSVGMLFNLLPLRFDRPEPSV
	ggcgagcacacaacttttcctccgcagtttcgtcaacctccttgaggaagtggcgaagggtgttgatatgactatggaatccctgcctacgtggtccaagtcagatattgac	KFSDVIQTARTKAYAALEHSSVPFDILLNDLNVPR
	catgctcttgatgtgggaagaggtgagccgggatctttagtaccctttgcacgtagagtttgcatactgacgcgcctaacaggcactgacatgaaccttgaatggccgggt	SSSAAPIFQILVDYRLVVQERTTYAGCKLSDEQW
	acgatatctcaccgcgtggaccagatgattcaagagcatgctaccgatatcgcgcttaaagatggatacggccgggttctaacatatcacggcatgggagagcaaatc	HAARIGYDMGLEITENPTGESKLTLRMQDALYSQ
	aagagcattgcccacgccctccgtgctgccaaagtacctgaagggacgacagtcggtgtgttccagacaccttctgcagactggatctgctctttgctagccattctccga	ASTQLFLRSFVNLLEEVAKGVDMTMESLPTWSK
	gtgggtgcggtctacatgcctctggatatccgcaactcgatttcgcgcctgaaacgcatcgtcgattcggctcgcccggccctcatccttgttgacaatgcgatgaagcca	SDIDHALDVGRGTDMNLEWPGTISHRVDQMIQE
	cagatccaggagattgcaccgcatcatatcccttccatcaacgtctctgagcttgaagctgtctccagtgatatcgaattcaacgccgcgagaccagagtcgcccgccat	HATDIALKDGYGRVLTYHGMGEQIKSIAHALRAA
	cgccatgtttacgagtggctcaactggtgagcccaagggcattctcatgtcccatgctagtctttgcgcacattttgagggcttccaccgcgcctttgacatcgaatcgatgg	KVPEGTTVGVFQTPSADWICSLLAILRVGAVYMP
	cacagatggttttacagcacagcacctatagcttcgactactcgctcaatcagatcttcgcggctctcgccggtggtggctgtctgtacgttgccccaggtgacattcgaggt	LDIRNSISRLKRIVDSARPALILVDNAMKPQIQEIA
	gatccacacgagctcgccaaggctataatccagcagggcatcacctatactgccacaaccccatctgagtatgaaatgtggtttcgctttgccatcgacaagctgcgag	PHHIPSINVSELEAVSSDIEFNAARPESPAIAMFTS
	agagcacaacatggaaggcagcgtggttcggtggcgaggttacgaacccaagtgtgattcatggcttccgcagcctcggtttgccagactttcgcacgttctctggctac	GSTGEPKGILMSHASLCAHFEGFHRAFDIESMAQ
	ggcccagctgagatgactgtttcttcaacaaaggccgaagtattctaccgcgacgaggacattcacttcccgttaccgggaggcttcatgctcccgaactactgcgcctat	MVLQHSTYSFDYSLNQIFAALAGGGCLYVAPGDI
	gttgttaatgagaaccttgaaccagttccggtgggcgtccctggcgagatagtcctagggggcgttggagtcgccatgggatacatgggccaagatgacttaacaaagc	RGDPHELAKAIIQQGITYTATTPSEYEMWFRFAID
	agcaatttctaccggaccccttcatgaagacgcacccgcactatgtggcacataactggaaccgtatgtatcgcagcggtgaccgcggctatcttcgcgaggacggcg	KLRESTTWKAAWFGGEVTNPSVIHGFRSLGLPD
	ccatatattgtgtcggtcgtatcgatggcgacacccaggtcaagctgcggggtttccgtgttgagcttggcgagattgaaaatgtcatcatgaaggaagcagccggcact	FRTFSGYGPAEMTVSSTKAEVFYRDEDIHFPLPG

	cttgctcaaaccgcagtcaacttgcgcgatggggttttaatcgcgcacgttgtgtttgagccagcgacattgggcaccgcccgacaatcgttactcgacaagcttcgatct<br>agcctgccacttccgccatacatgcgtccctcgctattcgtgacattggatgagatgcctctgactagccatctcaagatagaccggaaggccatccaagccatgtccctt<br>ccagatgctgtgttgatcccaaccgacgcacagcgtgagctcacgcaaactgagaaagtgctcgctggactatggatggatgtccttccccacaggcccagcagtttg<br>gcacccatcaatgacttcttccatgttggtggcaattctctccttcttgtgcagcttcaggccatgatcaagcgtgcgtttggcacagctccacgcctggtcgacctcatgaac<br>agcagttccctggagacgatggcttccctgatcggtggcagctcggacaccggagccgtcaactgggacaccgaggttgccctagaccactctcttctcaaaaccgcg<br>gaggccatcaaagttacctcccctcgtcgtagcgagaatctccgtgtactcatgacaggggctactgggaacctcggcagctatatagtgccccagctacttcaggatac<br>ccgcgttggccaggtcatgtgtatcctccgcggcgggtctcagcacagtaaggcttctacattgcccaccgatcccaagatctccatggtggacgccgacctctcacttcc<br>caatctgggcctttcagaagtcgacttcaccgtccttgcccagactaccgatgtcgtgctccactgtgcagccaaccgcaacttctgggacgggtacgaggtcctccgtcc<br>agtcaatgtgaacagcgtgaaggctctcgccaatctggcgcttctcaaccaatcgaagcttcatgttctgtcctccggtgctgtagaggtgtacaagaccacccacccac<br>cctcagacggttcggacgggtacgtggcgagcaaatgggtcgtggaacagtacttgcgcaaggcagcgcaagctgttgggctcgatgcaacggtgcatcgccccga<br>agcagctagcactgactcttccagccaccaaagtgtcagggatgccgtcgaggagctgattaaggttgcgaagggaaggctgggtacgcgtcctgacttcgggcgag<br>ttgagggcacggtgcacatggcgccggtgcatgacattgcggaccgcattgttgcgaatgtctgcgagtcacaagacgaggaggcgcggagtcagagagatataaa<br>tgtactgcgctatgctggtactttgcgtgccactaccacagacctcgcgatgcatgcgagcgagatggctggtgccgcggagtttgaagctctcccaacgatgcctgtgct<br>acaatggtttggagaggcaaagagggcagggttcgggcagtttgtgacggcccaggagttggtgatcagtgatggcggactgcgtctggtgtcgaggcgatga	GFMLPNYCAYVVNENLEPVPVGVPGEIVLGGVG<br>VAMGYMGQDDLTKQQFLPDPFMKTHPHYVAHN<br>WNRMYRSGDRGYLREDGAIYCVGRIDGDTQVKL<br>RGFRVELGEIENVIMKEAAGTLAQTAVNLRDGVLI<br>AHVVFEPATLGTARQSLLDKLRSSLPLPPYMRPS<br>LFVTLDEMPLTSHLKIDRKAIQAMSLPDAVLIPTDA<br>QRELTQTEKVLAGLWMDVLPHRPSSLAPINDFFH<br>VGGNSLLLVQLQAMIKRAFGTAPRLVDLMNSSSL<br>ETMASLIGGSSDTGAVNWDTEVALDHSLLKTAEA<br>IKVTSPRRSENLRVLMTGATGNLGSYIVPQLLQD<br>TRVGQVMCILRGGSQHSKASTLPTDPKISMVDA<br>DLSLPNLGLSEVDFTVLAQTTDVVLHCAANRNFW<br>DGYEVLRPVNVNSVKALANLALLNQSKLHVLSSG<br>AVEVYKTTHPPSDGSDGYVASKWVVEQYLRKAA<br>QAVGLDATVHRPEAASTDSSSHQSVRDAVEELIK<br>VAKGRLGTRPDFGRVEGTVHMAPVHDIADRIVA<br>NVCESQDEEARSQRDINVLRYAGTLRATTTDLA<br>MHASEMAGAAEFEALPTMPVLQWFGEAKRAGF<br>GQFVTAQELVISDGGLRLVSRR*
ptsB	atgctccgttccgcccaggcggcgcgagcctggaagacggcctcaatcagccgtcccttcacccatcgtcgtcctcttccaattctttcatcaatccaaagaccactgtctc<br>ctgtacggcgtcatgctacgactgccaatgccatcactgagtgagttgtcttcgcccatttctcttacgcaccccatcccccccgtcgaggaacgcaagtcccccgagaat<br>gtgtatgctcataaacggcccaggaacgcacaagagagagtcaccccatccttcaacgcatcgacgtcgaaggatgtacagcccctgatcgatccacgccatggag<br>aaatggatgaatcgtatgttgatcgaccatcgccgcatccggtatattgccattgacagaggcccaattaggttcattggaatgactggaggggctatattccacgaaatg<br>atgaaacggcagggtgtcaagaacatctgtacgcagcaaccaaggcccataatatctatttcacgttgagatgataaccgtacctagtcgggtaccctggcggtgccat<br>tcttcccgtgttcgacgccatctacaactcccgaaactttgactttgttctgtcaaaacacgagcaaggtgctgggcatatggcggagggatatgcccgcgtatccggaaa<br>accaggggtggtcttggtgactagtggtcctggtgcgaccaacgtagtcaccccaatggccgacgcattggccgatggagttcccatggtggtgttctgcggccaggttg<br>ccacaaccgccattgggagtgacgcttttcaggaggcggacatggtaggaatctcccaagcgtgcaccaaatggaatgttatggtgaagagcgtggccgaattgccg<br>aggcgtatcaacgaagcctttgaaattgccacaagcggcaggccaggcccggtgctcgttgatctgcctagagacgtcacgggtggcatctgtcggaaagccatccct<br>acggagtccacccttcctcggtccgcctctgtcgcagcacgccaggccctagagttgaataagaagcagctgaaccagtcgattaaaaaggtagcgaaccttatcaac<br>aatgcccaaagcccagttatctttgccggccatggcgtcgtctgctcagaaggcggccccgagctgctgaagactctcgcggacaaggcgtccattcccatcaccactt<br>ctctccacggactcggagcatttgacgagcttgacgagaaggctctgcatatgttgggaatgcacgggtctgcatatgcaaatatggcgatccagaatgccgatctgatc<br>atcgcgctcggggggcgtttcgatgagcgggttacctgcaatatcaccaaattcgccccaaaggctcatgcggctgcaaaggaaggtcgaggtggtattgttcactttga<br>aataatgccgaaaaatatcaataaggttgtccaggccactgaggcagtcgagggtgatgtggctgccaatctacggctcttgattccccacgtccaggagaagtcgatg<br>tccgatcgcaaggcctggttcgaccagatcaatgaatggaagagaaagtggcctctcaatgacttcgagaaggccgaacgttctggatttatcaagccccagactctg<br>attgaagagctgagcaacttgacggcggaccgcaaagaccgcacctacatctcaacgggcgttggccaacaccagatgtgggtcgctcagcactttcgctggagac<br>atccacgtaccatgatcacatcgggcggtctgggaacgatgggatacggtctgcctgctgcaatcggcgctagcgttgccaaaccggacgccctcgttattgatatagat<br>ggtgacgcatcgtttgccatgagcctgaccgagatgtctaccgcctcacagttcaacatccccgtgaaggtcatcgttttgaacaatgaggaacaggggatgatcacac<br>aatggcagaacctatactacgaggatcgatacgctcactgccaccagaagaacccggactttgtcaagcttgccgagacgatgggattgcaggcccgccgggttatc<br>aaaccagatgacgttgttgatagcctgaagtggctgatcaacactaatggacccgctcttctcgaggttgtcaccgataagaaagtgcccgtgctgccgatggttcctggc<br>ggatctggtttggatgaattcatcacatgggatagtggtatgtattgctggccgtctaaacaactctcgctgaccacagtcgttaaaacagcaaaagataaggcacgaag<br>ggagttgatgcggcagcgtacgcgtggtcttcacgggtag	MLRSAQAARAWKTASISRPFTHRRPLPILSSIQRP<br>LSPVRRHATTANAITENAQERVTPSFNASTSKDV<br>QPLIDPRHGEMDESFIGMTGGAIFHEMMKRQGV<br>KNIFGYPGGAILPVFDAIYNSRNFDFVLSKHEQGA<br>GHMAEGYARVSGKPGVVLVTSGPGATNVVTPM<br>ADALADGVPMVVFCGQVATTAIGSDAFQEADMV<br>GISQACTKWNVMVKSVAELPRRINEAFEIATSGR<br>PGPVLVDLPRDVTGGICRKAIPTESTLPRSASVAA<br>RQALELNKKQLNQSIKKVANLINNAQSPVIFAGHG<br>VVCSEGGPELLKTLADKASIPITTSLHGLGAFDEL<br>DEKALHMLGMHGSAYANMAIQNADLIIALGGRFD<br>ERVTCNITKFAPKAHAAAKEGRGGIVHFEIMPKNI<br>NKVVQATEAVEGDVAANLRLLIPHVQEKSMSDR<br>KAWFDQINEWKRKWPLNDFEKAERSGFIKPQTLI<br>EELSNLTADRKDRTYISTGVGQHQMWVAQHFR<br>WRHPRTMITSGGLGTMGYGLPAAIGASVAKPDA<br>LVIDIDGDASFAMSLTEMSTASQFNIPVKVIVLNN<br>EEQGMITQWQNLYYEDRYAHCHQKNPDFVKLAE<br>TMGLQARRVIKPDDVVDSLKWLINTNGPALLEVV<br>TDKKVPVLPMVPGGSGLDEFITWDSAKDKARRE<br>LMRQRTRGLHG*
ptsC	atgagtacgacgactactactgccgccaccacaaccttcacctccaccctgaaggtggatgaatccatcgtcgaggatcccgccccagagatggacttcatccccaac<br>agtggaaacgtcttccctaaattagtcgacaagttcaacaagaccgccgtggaggtgtggcttttcgatgccatggccgccgatggcaccaccgcgttcaccgtgtccttc<br>gttcgcgacatcctcactgcaccggctgggttccgcattcaaatcaatgccactttctccgacggcaccaaatggaccagcccattgatcttcccagagtcgaccatcact<br>tcggagggccccgatctcggtcatgggcgcgtggtgggggtctggcgcaccgacaaggacactagtcaggcgggcttcgaggtggccgctgacctgtccacctccgtt<br>gtttcattcgatgtgcccggcaaaatcacaggcacgctgaagcaccggtcattggggtatcctggattacctcagacggcacgcgaagctcaaatggcgccggaggc<br>gtactggatgcgacccatccccattgctgatgccaccgtcgacatgacgttttacactaccaaccccgatggtgagacaaccagccggcgaatggtcatcgacgagga<br>catgcgcgccacgggcggcgtggaccgttcgtgggagcctatgccctggagcaaggtcatgactgactcatattttctgcgcgcaaaggccggtccttatgtgatccag<br>gtcatgcgattggtgggcaggccagagcagaactacgagctctatgcgacggcacgtctataccgcgacgggaaattggtgtgtgcgccactgcgggcgctgccacc<br>caatggagcggtggataaccctgggagcgacgacacggtagtggtggaaaagttattcgacggagagggtgtgctggcgatcttcagacacaagaacgttgggtgtc<br>gtgttgagttccgttcgggggggccctggtggagagcgatgggtgtttgaggcccgccatcaccgcgcctggtggagcaagccgtctagccctccagggcctaatgcaa<br>ctggccatgctgggtttgtcgcctccgtggtcggaggccaaacgggctcggcggaatcccaccagggctggggtatatctgggcaagttgatatgcccgagtaa	MSTTTTTAATTTFTSTLKVDESIVEDPAPEMDFIP<br>NSGNVFPKLVDKFNKTAVEVWLFDAMAADGTTA<br>FTVSFVRDILTAPAGFRIQINATFSDGTKWTSPLIF<br>PESTITSEGPDLGHGRVVGVWRTDKDTSQAGFE<br>VAADLSTSVVSFDVPGKITGTLKHRSLGYPGLPQ<br>TAREAQMAPEAYWMRPIPIADATVDMTFYTTNP<br>DGETTSRRMVIDEDMRATGGVDRSWEPMPWSK<br>VMTDSYFLRAKAGPYVIQVMRLVGRPEQNYELY<br>ATARLYRDGKLVCAPLRALPPNGAVDNPGSDDT<br>VVVEKLFDGEGVLAIFRHKNVGCRVEFRSGGPG<br>GERWVFEARHHRAWWSKPSSPPGPNATGHAG<br>FVASVVGGQTGSAESHQGWGISGQVDMPE*
ptsD	atgccctcgcccatcggaaccgcctttcatgggcaggcatccatccgctcatcgtgtgagcgctgtcgtttccaaaagttgaaatgcaccggacctgcgggtcctgacgg<br>ccacggtccatgccagcgatgcgcccgagccaaagtcgactgcgtgttcggtcgacgacggccaaccagccggattagcgagtccaagcgtcggtcagaggccac<br>cacgaacaacctttccagagcgactctccctaacccgctgaccccatcaacggcgtccgttacccccgttgtcaccatggccaatggcccgagcatcgagcctgcagc<br>agactcgacgatgtttgacatgcgcggatgggatagtgttcaattgcaggaggtcatggataaagccaccaactttgccaaccatctcgatcactatgacatgccgccg<br>gccaatgggttggatgatatcttcctgttcgacaccgacacggaaaacacggcgccaggttcgtcatcctcgcaggcagtacagaacccgcccgtaatgacagggaa<br>tatatgtggcaacagtgataacagtggtcttgtccagtcactgctcgctctgctttcagagatgcaacagcggctgagagtgctacaggagggtccatggcagtttggcag<br>tacctgtgggctagatgactaccccatcggcactgtgctccatctctctcagcaattcagtaccttggtaggcccctgtctccggggggctaccgtatcgtgttatgacccca<br>cgggaccattgtccagcgtcgacgggacaccgggggactctctcgccatggtcctagttttgagtgggtacatgtcgttgatgcgtctctatagcatcgtgcttggccacttt<br>caggcgcatctgagtcagatctcgaaccaggaggaggttcttgcccgcaactccttgagtccaacgcttcagctaggggagcttccctgcgccaccacgacccacggc<br>ctgggtcgtatccacatggcgctgtgtatgttgcaggactccctcaacagcgtggaagaccagttggggaaagggggcgagttcgcgcgacagatggtcatgacgctc<br>ctgagaaaggagaccttcttgaccaagggcaatttgcagcacggagaaagcggattgagcagacaagcaacggccgtgaagggcctattgcgggaaaagatgag<br>tctctag	MPSPIGTAFHGQASIRSSCERCRFQKLKCTGPA<br>GPDGHGPCQRCARAKVDCVFGRRRPTSRISESK<br>RRSEATTNNLSRATLPNPLTPSTASVTPVVTMAN<br>GPSIEPAADSTMFDMRGWDSVQLQEVMDKATN<br>FANHLDHYDMPPANGLDDIFLFDTDTENTAPGSS<br>SSQAVQNPPVMTGNICGNSDNSGLVQSLLALLS<br>EMQQRLRVLQEGPWQFGSTCGLDDYPIGTVLHL<br>SQQFSTLVGPCLRGATVSCYDPTGPLSSVDGTP<br>GDSLAMVLVLSGYMSLMRLYSIVLGHFQAHLSQI<br>SNQEEVLARNSLSPTLQLGELPCATTTHGLGRIH<br>MALCMLQDSLNSVEDQLGKGGEFARQMVMTLL<br>RKETFLTKGNLQHGESGLSRQATAVKGLLREKM<br>SL*
ptsE	atggtctcgataacccagactcaaactgccctcgttggcgacccagaagggaatatcgtgctctctcactcggcgcctgtgccagctctcgaagacgaccgcatcgctg<br>tcgctgtcaaagccgtctctctaaccccgtcgacaccaagatggtgggcgactaccacaccccgggggccatctccggctgcgatttcgccggtgtcgtcacggccgt	MVSITQTQTALVGDPEGNIVLSHSAPVPALEDDRI<br>AVAVKAVSLNPVDTKMVGDYHTPGAISGCDFAG

	tggacccgcggccgcagcccacggcaatatcaaagtcggcgaccgcatctgcgcagccatctcaggattgaatccgctccgtccggacaccggagccttcgcggcg<br>cacacaacaacgccatactgggcctccctcaagctaccgcccagctggagcttcgcagacggcgccagtctgggcacgccgtggatgaccgtcggcacggggctgt<br>tcaagagcctgggactaccaggcgggccgctcgaccccatcagccccaattccaaaggcaagatccccacggtgctggtcaacggcgggtccagcgcgacagga<br>accgcggcgttgcagctgctcaagctggccgggtaccggacggtggcgacgtgctcgccgcgcaacttcgagctggcgcggtcgttcggtgccgacgacgtcttcgat<br>tataactcgcccacctgtgccgaggatatccgcgcgctgacgcgcaactcgctgcgctacgccctcgactgcatcaccaccaccgacagtatgcgtctgtgttatgcgg<br>ctctcggccgctccggtggtcgctacacggctctggatgcatacagcgaggctgttgccggttcgcgcaaagtggtgcgttccgagtgggtgttggggccggaaatgctg<br>ggagaggatatcgcgtggccggagccgcatggtcgtccggcgaatcccgatctcaaggcgttttgcatcgagtggacgcgcacgcttcagacgcttttggataggaatc<br>tgattcgtacacatccactgttggtccgcgatactgggttggaaggagtgctgggcggatttgaggagattcgcgccaagaaggtctcgggacagaagctgatctacac<br>gctggggaaatag	VVTAVGPAAAAHGNIKVGDRICAAISGLNPLRPDT<br>GAFAAHTTTPYWASLKLPPSWSFADGASLGTPW<br>MTVGTGLFKSLGLPGGPLDPISPNSKGKIPTVLV<br>NGGSSATGTAALQLLKLAGYRTVATCSPRNFELA<br>RSFGADDVFDYNSPTCAEDIRALTRNSLRYALDC<br>ITTTDSMRLCYAALGRSGGRYTALDAYSEAVAGS<br>RKVVRSEWVLGPEMLGEDIAWPEPHGRPANPDL<br>KAFCIEWTRTLQTLLDRNLIRTHPLLVRDTGLEGV<br>LGGFEEIRAKKVSGQKLIYTLGK*
ptsF	atgtcttcgtatcaagtcgtggccaacgactccgcggagtcattgatggactcttggggaatcttaggagttccagtcaacagcagaatgttcgcctacctcgttgctgccg<br>ccctctttggcatcgtactctactctcgtcccacaacaaagtccaatgtcccactgctcaacccacgacactggtacgagtggactgacaatagagccaaacaatacttt<br>atcaaaaattccaagagtatgatcgaggaccaattcgcaaagacgcctgataggccgtttcgggtcatcagtgacactggcgtagcgacggtcctgccaccttgcctgg<br>cccatgagattcggaatgatccacgtctcaattttgggggattcacgaaaaaggtaggttttctttgagaaagtcaaggatctcgcttatgccgtgctctaggcagctccag<br>gtcatatccccggctttgagggattccgtgacgcgttccacgataaccctttgattcatgagatcaccatccgcgatctgacgaaggagctcgacaaagtgacagggcc<br>agtctcagaggaggcggaggtttccctgagccatctgtttggtgagagtagtggtgaggccatctgtgcggacactttaaataggaaatacaccgactaatctagataga<br>gtggcatgcccttccgctcacctcgaccgtgttcagcctcatcactcgtacgatggcgcgagtcttcctcggccctgagctgtgccgcaatgatgagtggcacgccatcac<br>caagaagtactccacggatgtttaccaagcctcatttgccctccgcgattggcccgtcgctcttcactccgtcgtgcactggttcctcacccccctgccagaacgcacgtgca<br>gatgtccaaagagcccgggagatcattgcgcctgttttggagcagcgtcgcaagtatagaaaagaagccgaggctggaaaaaaggatgtaccccaatacaacgac<br>gccatggagtggatcgagcgggccgctaagggccagccatacgatgcggccatcacgcagttgacgctgtccgtagtagcggtccacaatactactgaccttttctgcc<br>aggtattgattgacctcgcgaagaacactgacatgctggaaccccttcgcgacgagattaagaccgtcatcaaggatcacggctggaagaagacggccttataccac<br>ctaaagctcctcgacagcgtcatcaaagagagccagcgaatgaagcccatgctcctgggtaggttgattccactcaccacttgcgtgcgtccgtgaggccaggtattct<br>aacattgtttccagctgcgatgcgtcgtgtggccctagaggacgtcaccctgtcagatggtactatcctccccaaaggcgacatggtagccgtgtccacacacaatatgc<br>gggatccgaatctctatcctgaccctgacaagtgggacggacgccgcttcattcggatccgcgagaccgtccccggcaaggaaaacgctgcacagttggtgacgact<br>accccggagcatcttggattcggacacggttacaatgcttgtccagggcggttctttgcggcgaacgagatgaagattattctggttcatttgctgctcaagtatgaatggcg<br>actggctccggacactgacccgaaacccatcttctcgggcttcagtatccgactcgaccagtcgactcagatggagtttcggagacggtatggtgatgagggaattctga<br>aatttatggattag	MSSYQVVANDSAESLMDSWGILGVPVNSRMFAY<br>LVAAALFGIVLYSRPTTKSNVPLLNPRHWYEWTD<br>NRAKQYFIKNSKSMIEDQFAKTPDRPFRVISDTG<br>VATVLPPCLAHEIRNDPRLNFGGFTKKAAPGHIP<br>GFEGFRDAFHDNPLIHEITIRDLTKELDKVTGPVS<br>EEAEVSLSHLFGESSEWHALPLTSTVFSLITRTM<br>ARVFLGPELCRNDEWHAITKKYSTDVYQASFALR<br>DWPVALHSVVHWFLTPCQNARADVQRAREIIAP<br>VLEQRRKYRKEAEAGKKDVPQYNDAMEWIERAA<br>KGQPYDAAITQLTLSVVAVHNTTDLFCQVLIDLAK<br>NTDMLEPLRDEIKTVIKDHGWKKTALYHLKLLDSV<br>IKESQRMKPMLLAAMRRVALEDVTLSDGTILPKG<br>DMVAVSTHNMRDPNLYPDPDKWDGRRFIRIRET<br>VPGKENAAQLVTTTPEHLGFGHGYNACPGRFFA<br>ANEMKIILVHLLLKYEWRLAPDTDPKPIFSGFSIRL<br>DQSTQMEFRRRYGDEGILKFMD*
ptsG	atggggtcaaccgagctgttcaccgtggatcagattattcggcatttgaccaacacagcaccggacaaggtaatcatctcctatccgactcccgagctggaatacaccg<br>actacacatgcacacacatagacaagctcaccagggccgccgcaagtctttttccgcagacgctgaaggagtctcccaagcacggcgatgctcaggaggcccccgt<br>agtgggaattacaggaccctcgaacttggaatattatattactttcctggctttgctgcgactcggggtgactccactggtcctttcaccccggttagccgttcaaggctttgca<br>catttgctccgcgaaagtaactgtcgaacagttctcgcatcgaatcagctatgcagcgttctcgagcgcgtgaggtccgagttcgacatgtcggcgctggaaaccatccc<br>gatggcctcaatcgaggccctcgtggatcgccccggggacgagggattcgtgtttcgccagatggacatctccagcccgagcaaatcgcctgatttattgatgcatacg<br>ggcggtacaaccggcctgcccaagctcgtgcccctctgcacaagcgactggataaccctcacacagcggatcataggccgcgtcaacttgcccgacaccctgtctac<br>cctcccgatataccacagccatgggcaagggttgctgattcgatcattggtggcggggacgaagctcgctctcatgaatgcgctgaggccgattaccgcggggacggt<br>gctggctggtctcgacaagtcgggagtgtcggcgctcgataccgatccgtacacgctcaagatcctggccgaagatccagagggcattgagcgattgtccaggttgtgg<br>ttcgtcagcgtgggagggtccgcgcttcccgatcatcttggagatcatctcatcacgcgcggtgtcagactccgccagctatacgggcagaccgaaagcggcggcctg<br>atggcgccatcgtcagacactcaagacgggtggaactggctcgtccctatgccgcacgtcgagccttatctcaggttcgaaaaggtggacggggatcttcaccacctcg<br>ttgttctccctggtcttcagacgaagagacttacaaaccggcccgacggggcgtatgggacgaacgaccttttttaagcaacacccaaccgatcctcgaaaatggaaatt<br>cgctgcgagacacgatgatattattgtgatgctgaacggggagaaggcggactcaacaccactggaacatgctgtcggcggcagtgagtatgtccacgtagcggttgt<br>ctttggggccggtcgtagctccctgggtatgctcgtggttccctccgcgaaagcgaccggcctgtcgcgtgaagagctcaaggagaaaattgcagcgaatctagaccta<br>gggaactcgcgcgttccgggttatgccaaggtctcgacggactctgtggttttcaaagaggtcggcacgcaagtaccgatgacccccaaggcgactgtgatccggccg<br>caggttctcgcccagtttgcgtcggatatcgacgagttctacgcggagcgggagagggcgcagaatagtttagcgaaagctattcccgacgacctggtacacacggtg<br>gtccgacgcgttgtgtgcacgacgttgggggtacctgaggccggcgatggatctatgaccgatgccgatgacttctttacccagggaatggactcactgcttgcctcacat<br>gtgcgtgctcgtctgttgcgcgaggtcaatatgggaggtcgaacattgcgaactaatgtcgccttcgactacccaactatcacacagctttctcaacatattctagatgtgag<br>gcatggcagcagtgaaggggtcattgtatcgatcgaggagttggcacgaggtcttgtacggaaatacaccaggcaactaagccctgttgccacatctggcgagctggg<br>gacatgtgaatccctggcagaaattcccaatggcgcttcacaaagcatcgtggtgcgtacggtctgcttgaaagtctccatatcgtcactaatggaaaagttagctccttac<br>cggcgccacaggaaccatcggacgctttgttctatataacctccttatccgggacgacgttgagcgagtttattgcctcgtacgcaccaactcagacgaagccgccgtcc<br>aacgggtcagaaaggctctcgaaggtgctcgtctcctcgccgctctcgagtccagtcagctcgaaaaggtcacagcattatccagtaacattgcagccgccgaccttgg<br>cctttcggccgcagcgtacgtggcgctccgcgcttccgtcacgaccgtcatccacaacgcctgggccgtcaacttcaacatgcacctagccagtttcgagaacccgtcg<br>atcgcaagtgttctgcacctgttgaactttgcccgccaatccatccgggcctcaaaaccgaacttcgctttcatctccagcaccgcgacagtcctcaacgcccatacgacc<br>cccatcgccgagactcgccatggatgggacgtcgtcgggaatatgggatacgcgcaatccaaatgggtcgccgaggagatctgcacacacgctgcgcagcacgac<br>ggacccgacagccctaggatccagatcgtccgcgtcggccagatcgccggggacactgtccatgggatctggaacccgcgggaggctatcccatccgtggttcaga<br>cagcactgaccattggagcgttgccgacaatccagggggggcgatgagacgcacttttggctgccggtggatatctgtggagccgctgttgtgcagctcgccctgtcgtcg<br>ggggactcatcgtcctccccttctgtgttccacgtctctaacggagagggactttgctggaacgcggatttcctgccggccttgcgagaggctggtctgcagttcgacgagc<br>ttccccaacaggagtgggtgagtcgcctggagcaatcggatccggatgtgcggcgcaatccgccgtaccggttgttggaccatttcagggagagatatggcaatgtga<br>cgttcaaggacgcgggtgatgagcaccattcccttgacatcagctgtgcaagggccagcgcgccggctttgaagcgcgcggtgcgcgtagataacttgcttgttggcaa<br>gttcgtgaagtattggttggatgttgcgtggaaggacttggaaaagggaccctctgaggattaa	MGSTELFTVDQIIRHLTNTAPDKVIISYPTPELEYT<br>DYTCTHIDKLTRAAASLFPQTLKESPKHGDAQEA<br>PVVGITGPSNLEYYITFLALLRLGVTPLVLSPRLAV<br>QGFAHLLRESNCRTVLASNQLCSVLERVRSEFD<br>MSALETIPMASIEALVDRPGDEGFVFRQMDISSP<br>SKSPDLLMHTGGTTGLPKLVPLCTSDWITLTQRII<br>GRVNLPDTLSTLPIYHSHGQGLLIRSLVAGTKLAL<br>MNALRPITAGTVLAGLDKSGVSALDTDPYTLKILA<br>EDPEGIERLSRLWFVSVGGSALPDHLGDHLITRG<br>VRLRQLYGQTESGGLMAPSSDTQDGWNWLVPM<br>PHVEPYLRFEKVDGDLHHLVVLPGLQTKRLTNRP<br>DGAYGTNDLFKQHPTDPRKWKFAARHDDIIVML<br>NGEKADSTPLEHAVGGSEYVHVAVVFGAGRSSL<br>GMLVVPSAKATGLSREELKEKIAANLDLGNSRVP<br>GYAKVSTDSVVFKEVGTQVPMTPKATVIRPQVLA<br>QFASDIDEFYAERERAQNSLAKAIPDDLVHTVVR<br>RVVCTTLGVPEAGDGSMTDADDFFTQGMDSLLA<br>SHVRARLLREVNMGGRTLRTNVAFDYPTITQLSQ<br>HILDVRHGSSEGVIVSIEELARGLVRKYTRQLSPV<br>ATSGELGTCESLAEIPNGASQSIVLLTGATGTIGR<br>FVLYNLLIRDDVERVYCLVRTNSDEAAVQRVRKA<br>LEGARLLAALESSQLEKVTALSSNIAAADLGLSAA<br>AYVALRASVTTVIHNAWAVNFNMHLASFENPSIA<br>SVLHLLNFARQSIRASKPNFAFISSTATVLNAHTT<br>PIAETRHGWDVVGNMGYAQSKWVAEEICTHAAQ<br>HDGPDSPRIQIVRVGQIAGDTVHGIWNPREAIPS<br>VVQTALTIGALPTIQGGDETHFWLPVDICGAAVV<br>QLALSSGDSSSSPSVFHVSNGEGLCWNADFLPA<br>LREAGLQFDELPQQEWVSRLEQSDPDVRRNPP<br>YRLLDHFRERYGNVTFKDAGDEHHSLDISCARAS<br>APALKRAVRVDNLLVGKFVKYWLDVAWKDLEKG<br>PSED*

Table S3. Primers used in this study.

Primer	Sequence (5′ to 3′)
ptsA-F1	TCGAGCTCGGTACCCTACCACGATGGCGGCATCTCAAC
ptsA-F2	TCGTTCTCACAAGCCGAAATCCCAAGATTC
ptsA-R1	ATTTCGGCTTGTGAGAACGACATTGCG
ptsA-R2	CTACTACAGATCCCCGCGAAACTTTGACTTCACCTC
ptsB-F	TCGAGCTCGGTACCCTCATCAGAATGCTCCGTTCCGCC
ptsB-R	CTACTACAGATCCCCATCTACCCGTGAAGACCACG
ptsC-F	TCGAGCTCGGTACCCATGAGTACGACGACTACTACTGCC
ptsC-R	CTACTACAGATCCCCACACTTCTCTCCTCTATTTGCC
ptsE-F	TCGAGCTCGGTACCCATGGTCTCGATAACCCAGACTC
ptsE-R	CTACTACAGATCCCCATGGCTATTTCCCCAGCGTGTAG
ptsF-F	TCGAGCTCGGTACCCGATGTCTTCGTATCAAGTCGTGGC
ptsF-R	CTACTACAGATCCCCGACGCGCATGTAAAGTACATGC
ptsG-F	TCGAGCTCGGTACCCATGGGGTCAACCGAGCTGTT
ptsG-R	CTACTACAGATCCCCTCTAGGAGAGCACAACACTACG
InF-pAdeA_XbaI-F	GCAGGTCGACTCTAGCCCATCATGGTGTTTTGATC
InF-pAdeA_XbaI-R	TAGTAGATCCTCTAGGTAAGATACATGAGCTTCGG
InF-pTAex3_SdaI-F	TGTACTTCTTGTTGCATGCCCCCATCATGGTGTTTTGATC
InF-pTAex3_SdaI-R	GCAGACTCTAGAGTCGAACCGTAAGATACATGAGCTTCGGTG
InF-linker-F1	GCTCGCGAGCGCGTTCCACTGCATCATCAGTCTAG
InF-linker-R1	AACGCGCTCGCGAGCAAGTACCATACAGTACCGCG
InF-linker-R2	TAAACGCGCACGCGACATTAATCCGGATCCTTTCC
InF-linker-F2	TCGCGTGCGCGTTTACCCATCATGGTGTTTTGATC
NdeI-ptsG_A-F	CGCGCGGCAGCCATATGGGGTCAACCGAGCTGTTC
EcoRI-ptsG_A-R	GACGGAGCTCGAATTCAAGCTTTCGCTAAACTATTCTGCGC
NdeI-ptsG_T-F	CGCGCGGCAGCCATATGGCGAAAGCTATTCCCGACGAC
ptsG_R-R1	CACGATGCTTTGTGAAGCGC
ptsG_R-F1	TCACAAAGCATCGTGCTCCTTACCGGCGCCACAGGAA
EcoRI-ptsG_R-R	GACGGAGCTCGAATTGTCGCATGTTAATCCTCAGAGGG
BlnI-ilv2-F	CTCATATACACCTAGATGATCAGACAATCTACGC
BlnI-ilv2-R	CCGTTTAAACCCTAGTCAGTGCTTACCGCCTGTAC
BlnI-ptsB-F	CTCATATACACCTAGATCAGAATGCTCCGTTCCGCC
BlnI-ptsB-R	CCGTTTAAACCCTAGCATCTACCCGTGAAGACCACG
NdeI-ilv2_ΔN57-F	CGCGCGGCAGCCATATGGCTCCAAGTTTCAATGTTGATCCA
EcoRI-ilv2_ΔN57-R	GACGGAGCTCGAATTGTGCTTACCGCCTGTACG

Table S4. Plasmids constructed in this study and PCR conditions for the amplification of the inserts for the plasmid constructions.

Plasmid	Inserts	Primer 1	Primer 2	PCR template	Vector
pPyrG-HR-ptsA	1st half of *ptsA*<br>2nd half of *ptsA*	PtsA-F1<br>PtsA-F2	PtsA-R1<br>PtsA-R2	gDNA of *A. pseudoterreus*	pPyrG-HR digested with *Sma*I
pTAex3-HR-ptsB	*ptsB*	PtsB-F	PtsB-R	gDNA of *A. pseudoterreus*	pTAex3-HR digested with *Sma*I
pTAex3-HR-ptsC	*ptsC*	PtsC-F	PtsC-R	gDNA of *A. pseudoterreus*	pTAex3-HR digested with *Sma*I
pTAex3-HR-ptsE	*ptsE*	PtsE-F	PtsE-R	gDNA of *A. pseudoterreus*	pTAex3-HR digested with *Sma*I
pTAex3-HR-ptsF	*ptsF*	PtsF-F	PtsF-R	gDNA of *A. pseudoterreus*	pTAex3-HR digested with *Sma*I
pTAex3-HR-ptsG	*ptsG*	PtsG-F	PtsG-R	gDNA of *A. pseudoterreus*	pTAex3-HR digested with *Sma*I
pTAex3-HR-ptsCE	PamyB-*ptsC*-TamyB	InF-pTAex3_SdaI-F	InF-pTAex3_SdaI-R	pTAex3-HR-ptsC	pTAex3-HR-PtsE digested with *Sda*I
pAdeA-HR-ptsF	PamyB-*ptsF*-TamyB	InF-pAdeA_XbaI-F	InF-pAdeA_XbaI-R	pTAex3-HR-ptsF	pAdeA-HR digested with *Xba*I
pAdeA-HR-ptsG	PamyB-*ptsG*-TamyB	InF-pAdeA_XbaI-F	InF-pAdeA_XbaI-R	pTAex3-HR-ptsG	pAdeA-HR digested with *Xba*I
pAdeA-HR-ptsFG	PamyB-*ptsF*-TamyB<br>PamyB-*ptsG*-TamyB	InF-pAdeA_XbaI-F<br>InF-linker-F1	InF-linker-R1<br>InF-pAdeA_XbaI-R	pTAex3-HR-ptsF<br>pTAex3-HR-ptsG	pAdeA-HR digested with *Xba*I
pAdeA-HR-ptsFGB	PamyB-*ptsF*-TamyB<br>PamyB-*ptsG*-TamyB<br>PamyB-*ptsB*-TamyB	InF-pAdeA_XbaI-F<br>InF-linker-F1<br>InF-linker-F2	InF-linker-R1<br>InF-linker-R2<br>InF-pAdeA_XbaI-R	pTAex3-HR-ptsF<br>pTAex3-HR-ptsG<br>pTAex3-HR-ptsB	pAdeA-HR digested with *Xba*I
pET28a-ptsG_A	*ptsG* (A domain)	NdeI-ptsG_A-F	EcoRI-ptsG_A-R	pTAex3-HR-ptsG	pET28a digested with *Nde*I & *Eco*RI
pET28a-ptsG_TR	1st half of *ptsG* (T–R didomain)<br>2nd half of *ptsG* (T–R didomain)	NdeI-ptsG_T-F<br>ptsG_R-F1	ptsG_R-R1<br>EcoRI-ptsG_R-R	pTAex3-HR-ptsG	pET28a digested with *Nde*I & *Eco*RI
pAT426-ilv2	*ilv2*	BlnI-ilv2-F	BlnI-ilv2-R	gDNA of *S. cerevisiae*	pAT426 digested with *Bln*I
pAT426-ptsB	*ptsB*	BlnI-ptsB-F	BlnI-ptsB-R	cDNA	pAT426 digested with *Bln*I
pET28a-ilv2 (ΔN57)	*ilv2* (ΔN57)	NdeI-ilv2_ΔN57-F	EcoRI-ilv2_ΔN57-R	gDNA of *S. cerevisiae*	pET28a digested with *Nde*I & *Eco*RI

Table S5. *Aspergillus oryzae* transformants constructed in this study.

Strain	Host strain	Plasmids used for transformation
A. oryzae/*ptsACE*	*A. oryzae* NSARU1	pPyrG-HR-ptsA, pTAex3-HR-ptsCE
A. oryzae/*ptsACEF*	*A. oryzae* NSARU1	pPyrG-HR-ptsA, pTAex3-HR-ptsCE, pAdeA-HR-ptsF
A. oryzae/*pptsACEFG*	*A. oryzae* NSARU1	pPyrG-HR-ptsA, pTAex3-HR-ptsCE, pAdeA-HR-ptsFG
A. oryzae/*ptsABCEFG*	*A. oryzae* NSARU1	pPyrG-HR-ptsA, pTAex3-HR-ptsCE, pAdeA-HR-ptsFGB

BGCs Overview

1 2 3 4 5 6 7 8

BGC5 - Aspergillus pseudoterreus	
**Scaffold / Position**	PIJX01000031.1 / 256596...286465
**Length (bp)**	29870
**Core enzymes**	PKS-NRPS, NRPS-like
**Duplicated fungal proteins**	Acetolactate synthase catalytic subunit, mitochondrial
**Duplicated human proteins**	–
**Similar BGC / Similarity score**	FBGC00093 (Sch 210972) / 28.3

Download BGC GenBank file

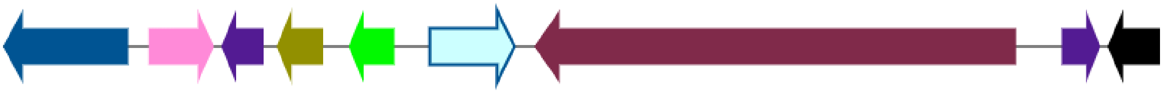

Gene information	
**Locus tag / Position**	A_pseudoterreus_005181 / 11002...13194 (+)
**Predicted function**	–
**Predicted domain organization**	–
**Closest homologue in FunBGCs**	acetolactate synthase CfoL (FBGC00534); identity: 72.3%
**Homologue in A. fumigatus**	AFUA_3G10310; Acetolactate synthase catalytic subunit, mitochondrial; 69.1% (duplicated)
**Human homologue**	–
**Representative protein families**	TPP_enzyme_N, TPP_enzyme_M, TPP_enzyme_C
**Amino acid length**	691

Copy AA sequence to clipboard

BLASTp on this protein

Figure S1. Example of output from FunBGCeX. The biosynthetic gene cluster (BGC) displayed here corresponds to the *pts* cluster.

BGC1

BGCs Overview

1 2 3 4 5 6 7 8

BGC1 - Aspergillus terreus NIH2624	
**Scaffold / Position**	NT_165939 / 36186...87559
**Length (bp)**	51374
**Core enzymes**	HR-PKS, HR-PKS
**Duplicated fungal proteins**	3-hydroxy-3-methylglutaryl-coenzyme A reductase 1
**Duplicated human proteins**	3-hydroxy-3-methylglutaryl-CoA reductase
**Similar BGC / Similarity score**	FBGC00278 (lovastatin) / 99.5

Download BGC GenBank file

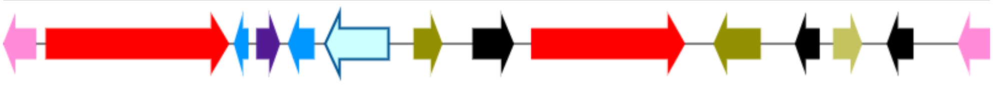

Gene information	
**Locus tag / Position**	ATEG_09965 / 16762...20071 (-)
**Predicted function**	HMG-CoA reductase
**Predicted domain organization**	–
**Closest homologue in FunBGCs**	HMG-CoA reductase LovR (FBGC00278); identity: 100.0%
**Homologue in A. fumigatus**	AFUA_2G03700; 3-hydroxy-3-methylglutaryl-coenzyme A reductase 1; 51.4% (duplicated)
**Human homologue**	3-hydroxy-3-methylglutaryl-CoA reductase; 53.7% (duplicated)
**Representative protein families**	HPIH, Sterol-sensing, HMG-CoA_red
**Amino acid length**	1048

Copy AA sequence to clipboard

BLASTp on this protein

BGC2

BGCs Overview

1 2 3 4 5 6 7 8

BGC2 - Aspergillus terreus NIH2624	
**Scaffold / Position**	NT_165938 / 371434...389192
**Length (bp)**	17759
**Core enzymes**	HR-PKS
**Duplicated fungal proteins**	ATP synthase subunit beta, mitochondrial
**Duplicated human proteins**	–
**Similar BGC / Similarity score**	FBGC00285 (citreoviridin) / 99.5

Download BGC GenBank file

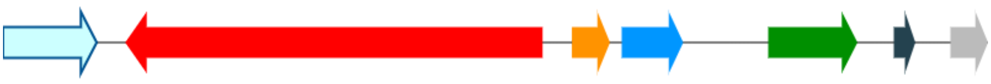

Gene information	
**Locus tag / Position**	ATEG_09616 / 1...1691 (+)
**Predicted function**	ATP synthase
**Predicted domain organization**	–
**Closest homologue in FunBGCs**	ATP synthase subunit beta CtvE (FBGC00285); identity: 97.4%
**Homologue in A. fumigatus**	AFUA_5G10550; ATP synthase subunit beta, mitochondrial; 80.6% (duplicated)
**Human homologue**	–
**Representative protein families**	ATP-synt_ab_N, ATP-synt_ab
**Amino acid length**	413

Copy AA sequence to clipboard

BLASTp on this protein

(to be continued on the next page)

BGC3

BGCs Overview

1 2 3 4 5 6 7 8

BGC3 - Aspergillus terreus NIH2624	
**Scaffold / Position**	NT_165937 / 159864...203620
**Length (bp)**	43757
**Core enzymes**	NRPS, NRPS
**Duplicated fungal proteins**	Phospho-2-dehydro-3-deoxyheptonate aldolase, tyrosine-inhibited
**Duplicated human proteins**	–
**Similar BGC / Similarity score**	FBGC00350 (asperphenamate) / 56.1

Download BGC GenBank file

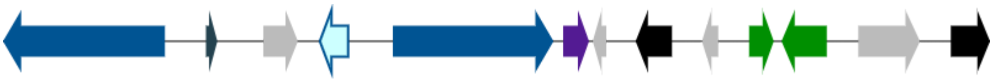

Gene information	
**Locus tag / Position**	ATEG_09067 / 14046...15301 (-)
**Predicted function**	phospho-2-dehydro-3-deoxyheptonate aldolase
**Predicted domain organization**	–
**Closest homologue in FunBGCs**	phospho-2-dehydro-3-deoxyheptonate aldolase ApmC (FBGC00350); identity: 84.1%
**Homologue in A. fumigatus**	AFUA_7G04070; Phospho-2-dehydro-3-deoxyheptonate aldolase, tyrosine-inhibited; 64.7% (duplicated)
**Human homologue**	–
**Representative protein families**	DAHP_synth_1
**Amino acid length**	379

Copy AA sequence to clipboard

BLASTp on this protein

BGC4

BGCs Overview

1 2 3 4 5 6 7 8

BGC4 - Aspergillus terreus NIH2624	
**Scaffold / Position**	NT_165937 / 228330...245391
**Length (bp)**	17062
**Core enzymes**	HR-PKS
**Duplicated fungal proteins**	Pheromone-regulated membrane protein 10
**Duplicated human proteins**	–
**Similar BGC / Similarity score**	FBGC00669 (cordycepin) / 16.9

Download BGC GenBank file

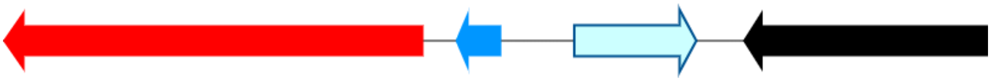

Gene information	
**Locus tag / Position**	ATEG_09090 / 9894...12012 (+)
**Predicted function**	–
**Predicted domain organization**	–
**Closest homologue in FunBGCs**	–
**Homologue in A. fumigatus**	AFUA_3G13940; Pheromone-regulated membrane protein 10; 52.0% (duplicated)
**Human homologue**	–
**Representative protein families**	–
**Amino acid length**	653

Copy AA sequence to clipboard

BLASTp on this protein

(to be continued on the next page)

BGC5

BGCs Overview

1 2 3 4 5 6 7 8

BGC5 - Aspergillus terreus NIH2624	
**Scaffold / Position**	NT_165936 / 1163001...1192503
**Length (bp)**	29503
**Core enzymes**	TC (Class1)
**Duplicated fungal proteins**	Carboxyvinyl-carboxyphosphonate phosphorylmutase
**Duplicated human proteins**	–
**Similar BGC / Similarity score**	FBGC00306 (AM-toxin) / 4.2

Download BGC GenBank file

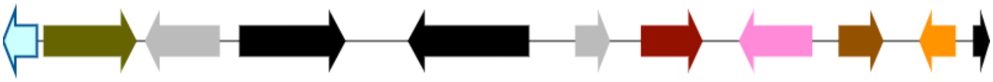

Gene information	
**Locus tag / Position**	ATEG_08912 / 1...1017 (-)
**Predicted function**	phosphoenolpyruvate mutase
**Predicted domain organization**	–
**Closest homologue in FunBGCs**	–
**Homologue in A. fumigatus**	AFUA_2G03820; Carboxyvinyl-carboxyphosphonate phosphorylmutase; 51.0% (duplicated)
**Human homologue**	–
**Representative protein families**	PEP_mutase
**Amino acid length**	307

Copy AA sequence to clipboard

BLASTp on this protein

BGC6

BGCs Overview

1 2 3 4 5 6 7 8

BGC6 - Aspergillus terreus NIH2624	
**Scaffold / Position**	NT_165935 / 528338...557513
**Length (bp)**	29176
**Core enzymes**	HR-PKS
**Duplicated fungal proteins**	Sphingolipid C4-hydroxylase SUR2
**Duplicated human proteins**	–
**Similar BGC / Similarity score**	FBGC00123 (1233A) / 12.8

Download BGC GenBank file

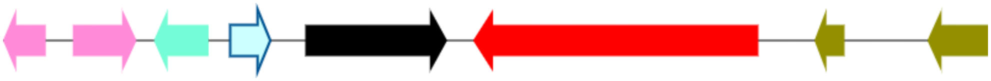

Gene information	
**Locus tag / Position**	ATEG_08170 / 6727...7923 (+)
**Predicted function**	fatty acid hydroxylase
**Predicted domain organization**	–
**Closest homologue in FunBGCs**	–
**Homologue in A. fumigatus**	AFUA_1G16850; Sphingolipid C4-hydroxylase SUR2; 52.8% (duplicated)
**Human homologue**	–
**Representative protein families**	FA_hydroxylase
**Amino acid length**	375

Copy AA sequence to clipboard

BLASTp on this protein

(to be continued on the next page)

BGC7

BGCs Overview

1 2 3 4 5 6 7 8

BGC7 - Aspergillus terreus NIH2624	
**Scaffold / Position**	NT_165933 / 1283154...1300349
**Length (bp)**	17196
**Core enzymes**	TC (UbiA)
**Duplicated fungal proteins**	Uracil phosphoribosyltransferase
**Duplicated human proteins**	–
**Similar BGC / Similarity score**	FBGC00026 (funiculolide D) / 7.6

Download BGC GenBank file

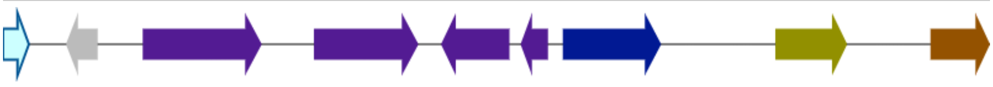

Gene information	
**Locus tag / Position**	ATEG_07297 / 1...451 (+)
**Predicted function**	–
**Predicted domain organization**	–
**Closest homologue in FunBGCs**	–
**Homologue in A. fumigatus**	AFUA_2G16200; Uracil phosphoribosyltransferase; 50.4% (duplicated)
**Human homologue**	–
**Representative protein families**	–
**Amino acid length**	132

Copy AA sequence to clipboard

BLASTp on this protein

BGC8

BGCs Overview

1 2 3 4 5 6 7 8

BGC8 - Aspergillus terreus NIH2624	
**Scaffold / Position**	NT_165929 / 463498...483837
**Length (bp)**	20340
**Core enzymes**	TC (Tri5)
**Duplicated fungal proteins**	Dihydroxy-acid dehydratase, mitochondrial
**Duplicated human proteins**	–
**Similar BGC / Similarity score**	FBGC00438 (aspterric acid) / 100.0

Download BGC GenBank file

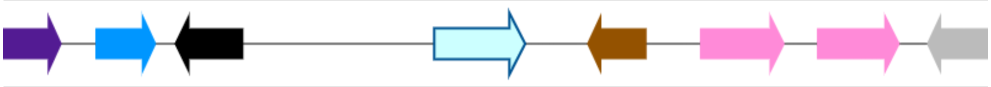

Gene information	
**Locus tag / Position**	ATEG_04415 / 8908...10781 (+)
**Predicted function**	dihydroxyacid dehydratase
**Predicted domain organization**	–
**Closest homologue in FunBGCs**	dihydroxyacid dehydratase AstD (FBGC00438); identity: 100.0%
**Homologue in A. fumigatus**	AFUA_2G14210; Dihydroxy-acid dehydratase, mitochondrial; 72.8% (duplicated)
**Human homologue**	–
**Representative protein families**	ILVD_EDD
**Amino acid length**	598

Copy AA sequence to clipboard

BLASTp on this protein

Figure S2. Results of FunBGCeX analysis of the *Aspergillus terreus* NIH 2624 genome using the resistance gene-guided genome mining mode. BGC1, BGC2, and BGC8 correspond to the biosynthetic gene clusters of lovastatin, citreoviridin, and aspterric acid, respectively. The Gene Information table displays details for each resistance gene candidate.

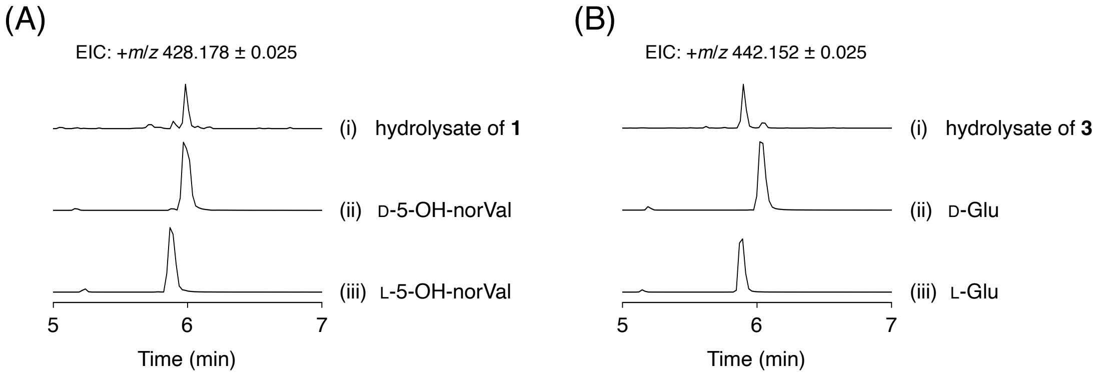

Figure S3. LC–MS analysis of L-DLA derivatives of (A) the hydrolysate of **1** and D- and L-5-hydroxynorvaline and (B) the hydrolysate of **3** and D- and L-glutamic acid.

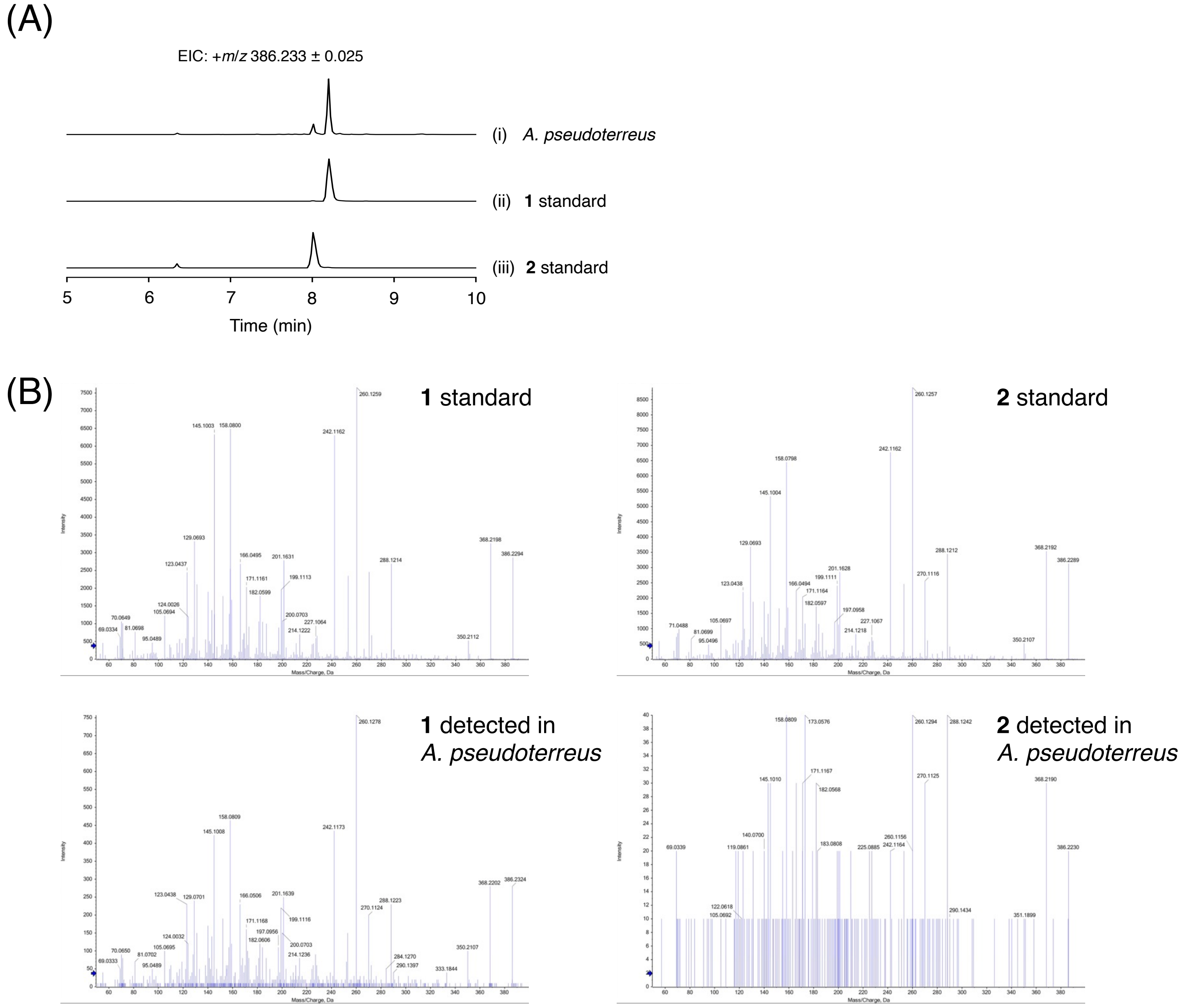

Figure S4. (A) LC–MS profile of the metabolites from *A. pseudoterreus* CBS 116.46 and (B) MS/MS spectra of **1** and **2** detected in *A. pseudoterreus* CBS 116.46 and their standards.

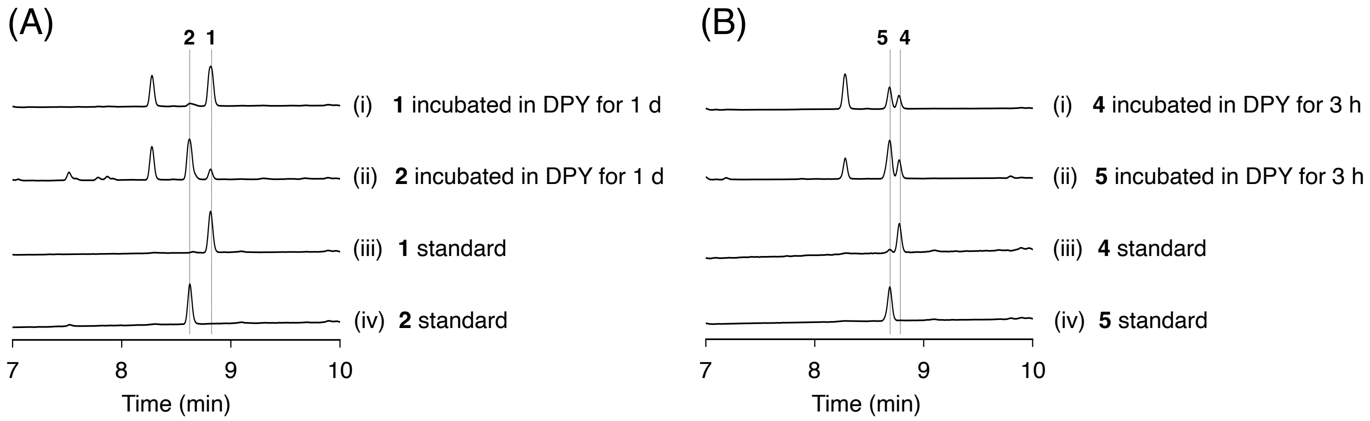

Figure S5. HPLC profiles of compounds **1**, **2**, **4**, and **5** incubated in DPY medium: (A) compounds **1** and **2** incubated for 1 day; (B) compounds **4** and **5** incubated for 3 hours. The chromatograms were monitored at 190 nm.

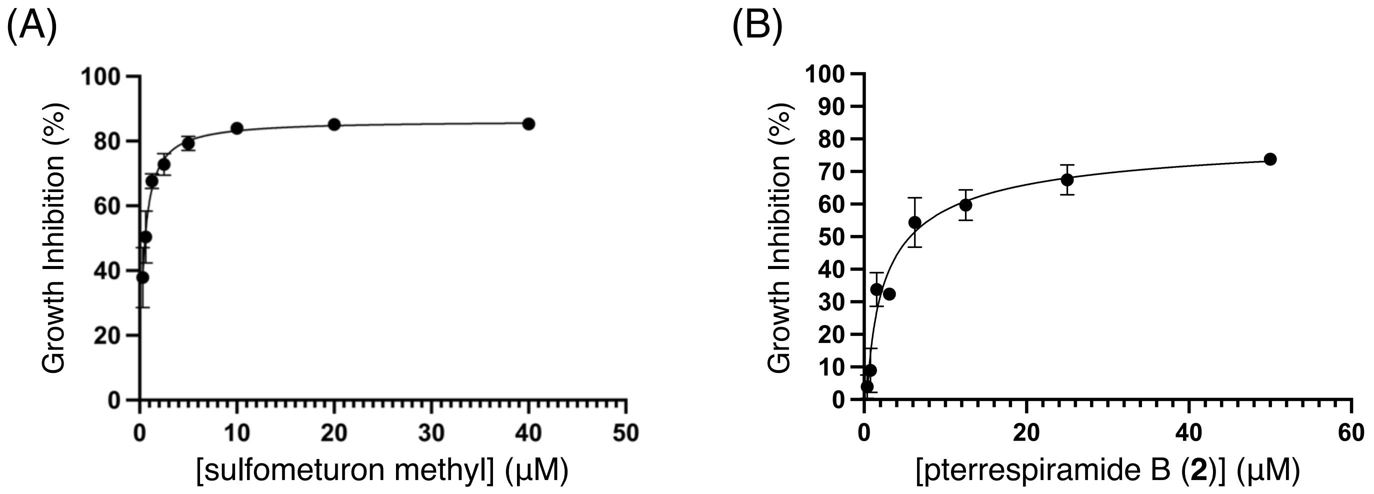

Figure S6. Growth inhibition of *S. cerevisiae* by (A) sulfometuron methyl and (B) pterrespiramide B (**2**).

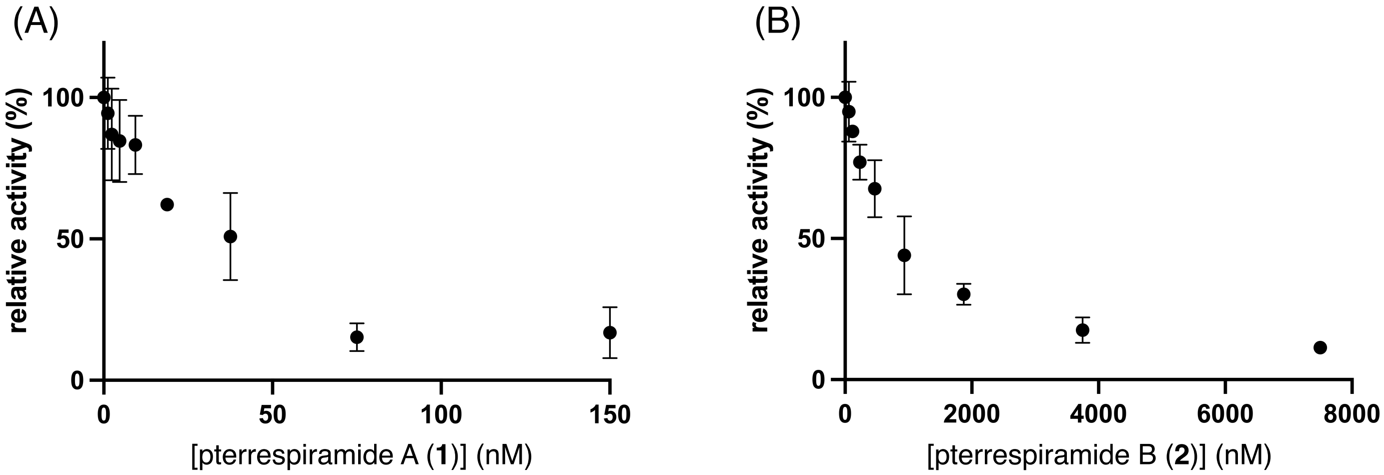

Figure S7. Inhibition of ScALS by (A) pterrespiramide A (**1**) and (B) pterrespiramide B (**2**).

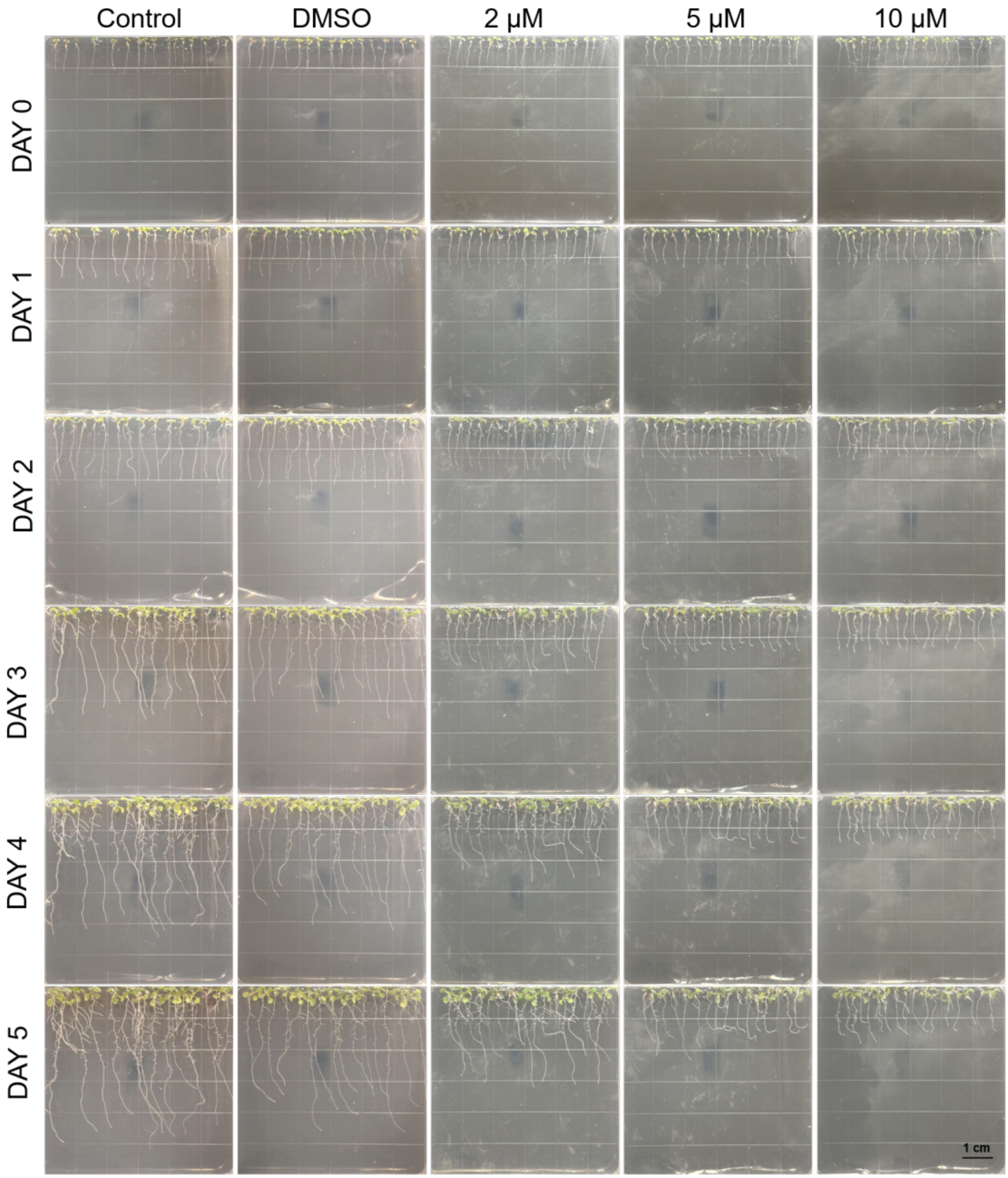

Figure S8. Representative images of *Arabidopsis* seedlings exhibiting reduced primary root growth after treatment with pterrespiramide A (**1**). *Arabidopsis* seedlings germinated on MS medium for 5 days were subsequently moved to fresh MS medium containing different concentrations of compound **1** (2, 5, or 10 µM) or DMSO for 5 days. Photographs were taken daily up to 5 days after transfer. Scale bar: 1 cm.

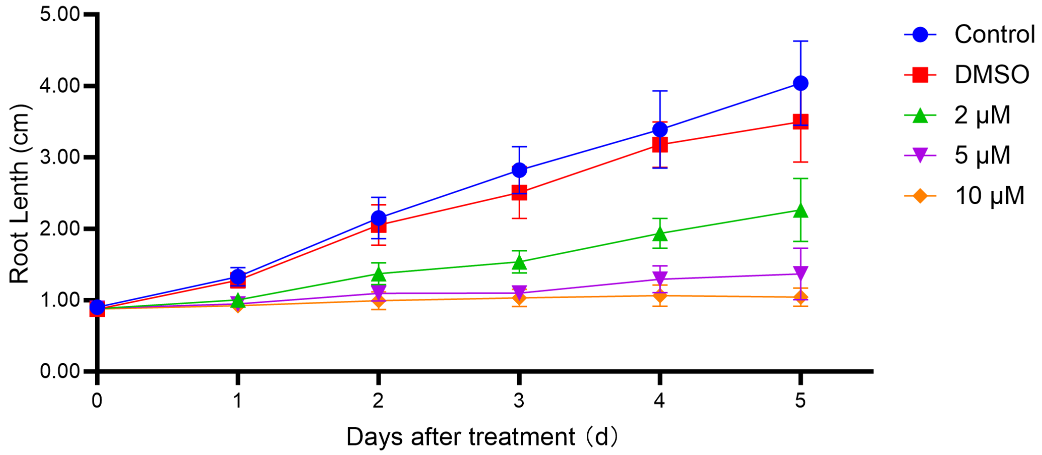

Figure S9. Primary root length measurement after compound **1** treatment. *Arabidopsis* seedlings germinated on MS medium for 5 days were subsequently moved to fresh MS medium containing different concentrations of compound **1** (0, 2, 5, or 10 μM) or DMSO for 5 days (days 0–5).

(A)

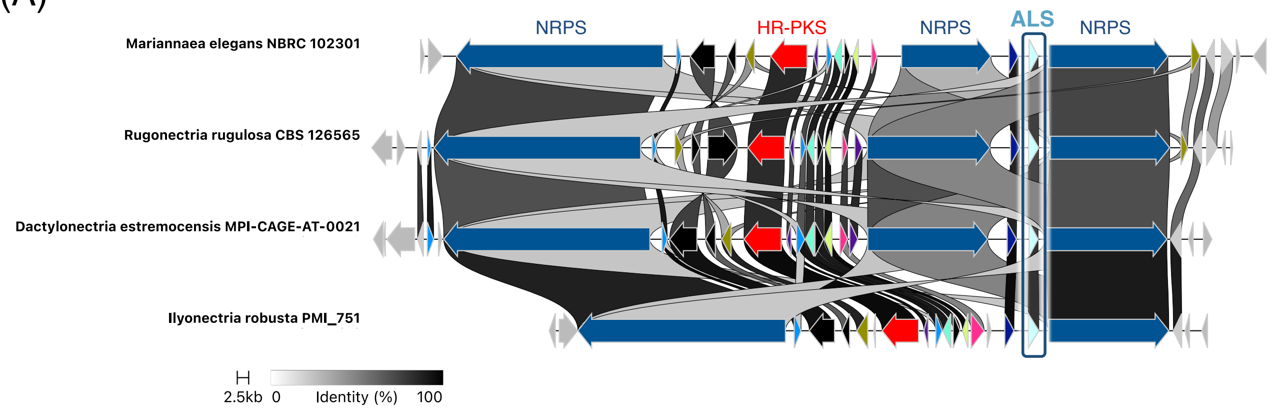

(B)

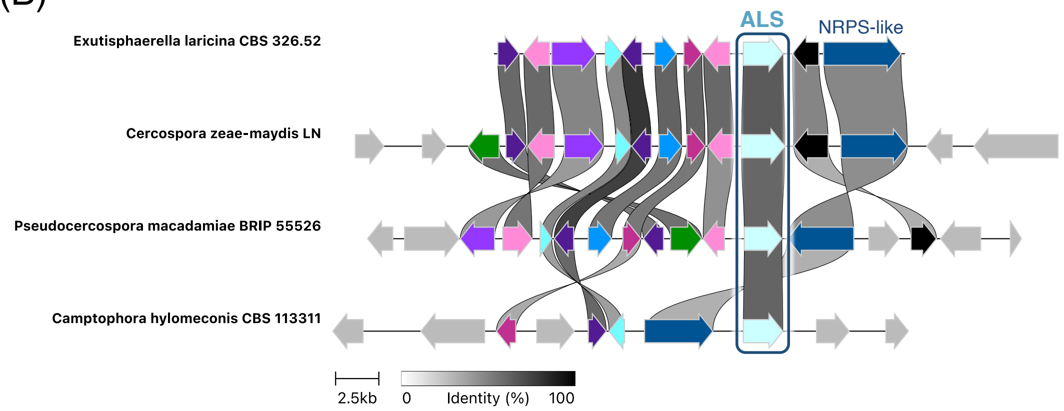

Figure S10. Selected gene clusters encoding acetolactate synthase (ALS) homologues from (A) Family 107 and (B) Families 39 and 296 (Supplementary Data 3). The comparison of gene clusters was performed using clinker.[15]

-	r157c2	NRPS-like,NRPS,T1PKS	scaffold_157	236803 - 302718	5	0

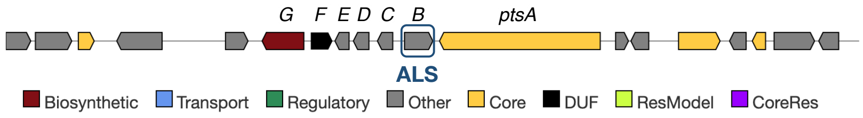

Sequence id	Location (start-end)	Type	Gene	Description	Function
5218	242404 - 243628	Core	168372at4890	Squalene monooxygenase: COG:C-H-E	Energy production and conversion
5235	302517 - 303955	Core	285140at4890	Pheromone a factor receptor: COG:T-M-K	Signal transduction mechanisms
5232	294460 - 295489	Core	344023at4890	mRNA-decapping enzyme subunit 1: COG:n/a	Unclassified
5230	288766 - 291976	Core	47936at4890	PH-like domain superfamily: COG:G-M-I-Q	Carbohydrate transport and metabolism
5227	270300 - 282718	Core	8502at4890	L-aminoadipate-semialdehyde dehydrogenase: COG:Q-I-H	Secondary metabolites biosynthesis, transport and catabolism

Figure S11. FunARTS analysis of the genome of *Aspergillus pseudoterreus* ATCC 32359. The genomic region corresponding to the *pts* cluster is shown. The *ptsB* gene, which encodes a self-resistance protein, was not recognized as a potential resistance gene in this analysis.

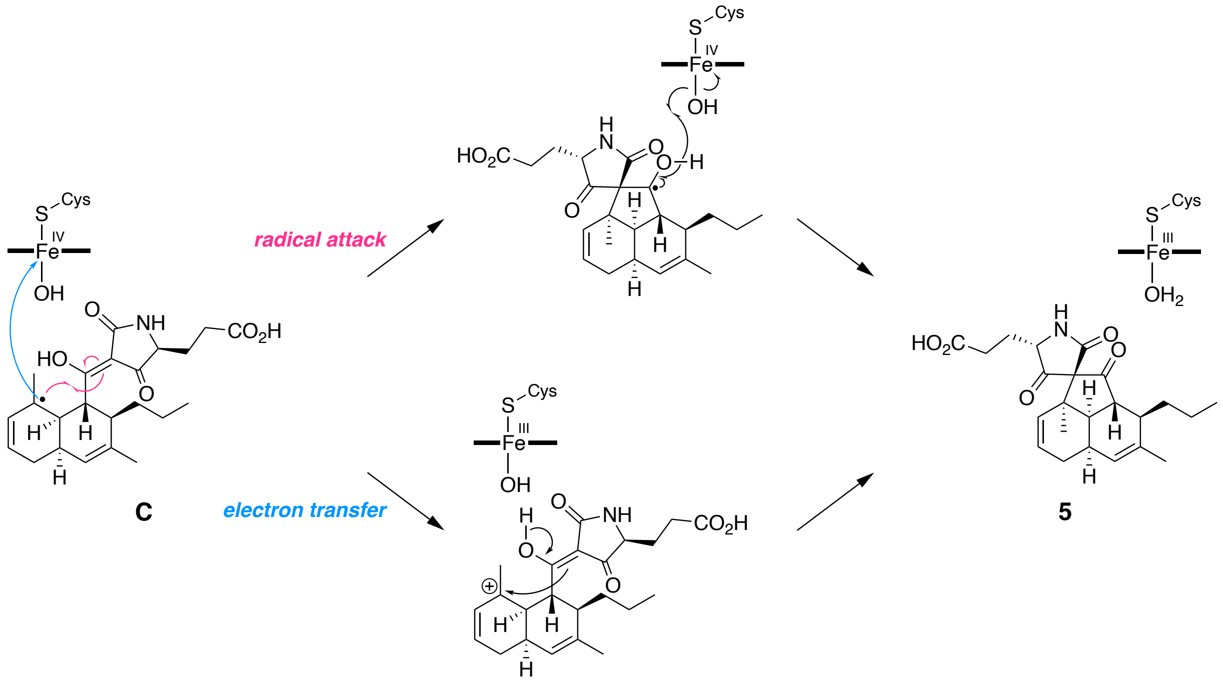

Figure S12. Alternative possible reaction mechanisms for the PtsF-catalyzed spirotetramate formation (also see Figure 6).

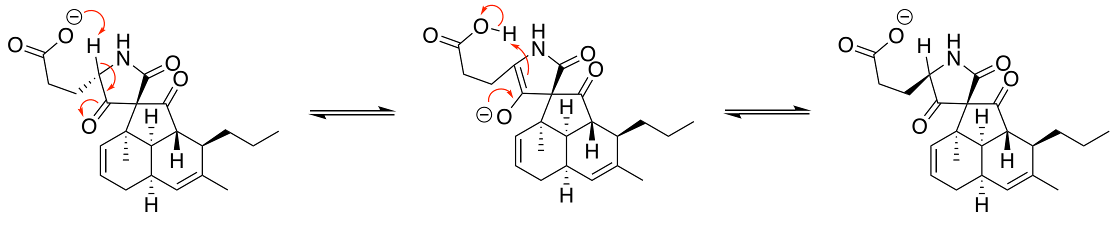

Figure S13. Proposed reaction mechanism of the epimerization between **4** and **5**.

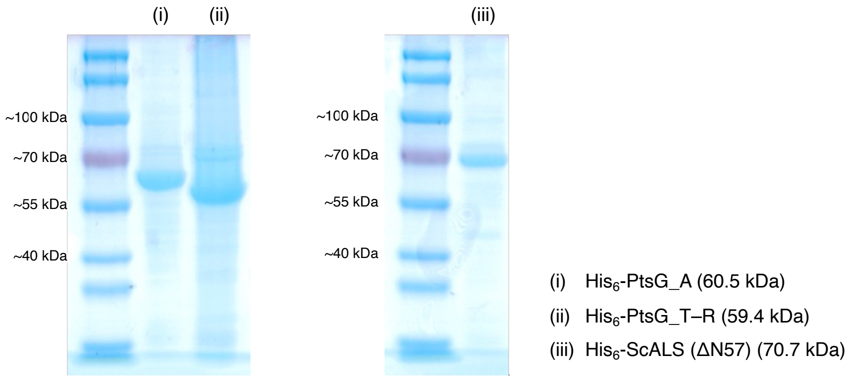

Figure S14. SDS-PAGE analysis of the purified protein.

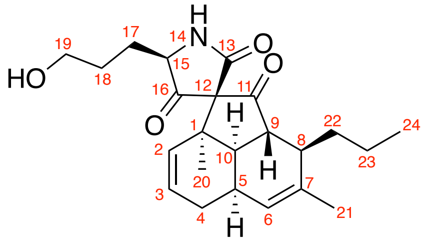

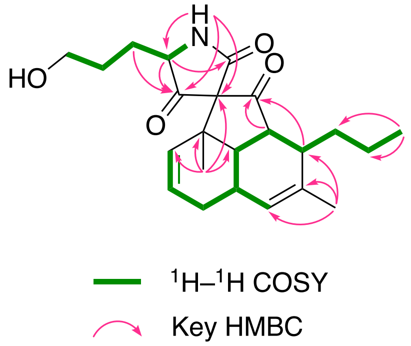

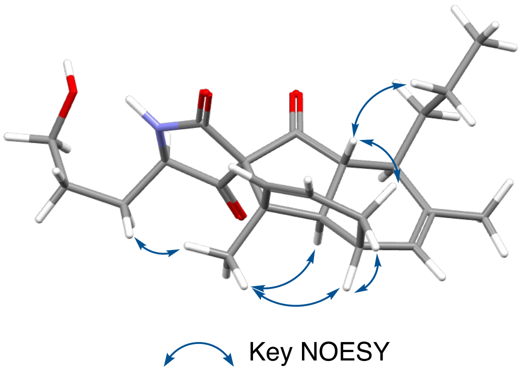

position	δ_C, type	δ_H, mult. (J in Hz)
1	46.0, C	
2	129.7, CH	5.89, dquin (10.4, 2.7)
3	124.0, CH	5.67, ddd (10.4, 5.2, 2.3)
4	27.9, CH_2	2.33 (α), dtd (18.9, 5.1, 1.4)
		1.75 (β), m
5	28.3, CH	2.47, m
6	129.2, CH	5.70, brd (6.1)
7	136.5, C	
8	41.4, CH	2.48, m
9	48.5, CH	2.56, dd (13.9, 10.3)
10	44.4, CH	2.39, ddd (13.9, 4.1, 1.3)
11	208.2, C	
12	75.1, C	
13	170.0, C	
14	–	7.45, s
15	62.7, CH	3.98, dd (9.2, 4.1)
16	207.8, C	
17	29.6, CH_2	1.97, m
		1.56, m
18	28.8, CH_2	1.76, m
		1.68, m
19	62.1, CH_2	3.76, ddd (10.6, 6.4, 4.6)
		3.67, ddd (10.6, 7.4, 4.3)
20	24.0, CH_3	1.18, s
21	20.6, CH_3	1.64, s
22	31.0, CH_2	1.90, m
		1.56, m
23	16.9, CH_2	1.22, m
		1.01, m
24	14.5, CH_3	0.86, t (7.3)

^{1}H NMR: 600 MHz, ^{13}C NMR: 150 MHz (in $CDCl_3$)

Figure S15. NMR data of pterrespiramide A (**1**).

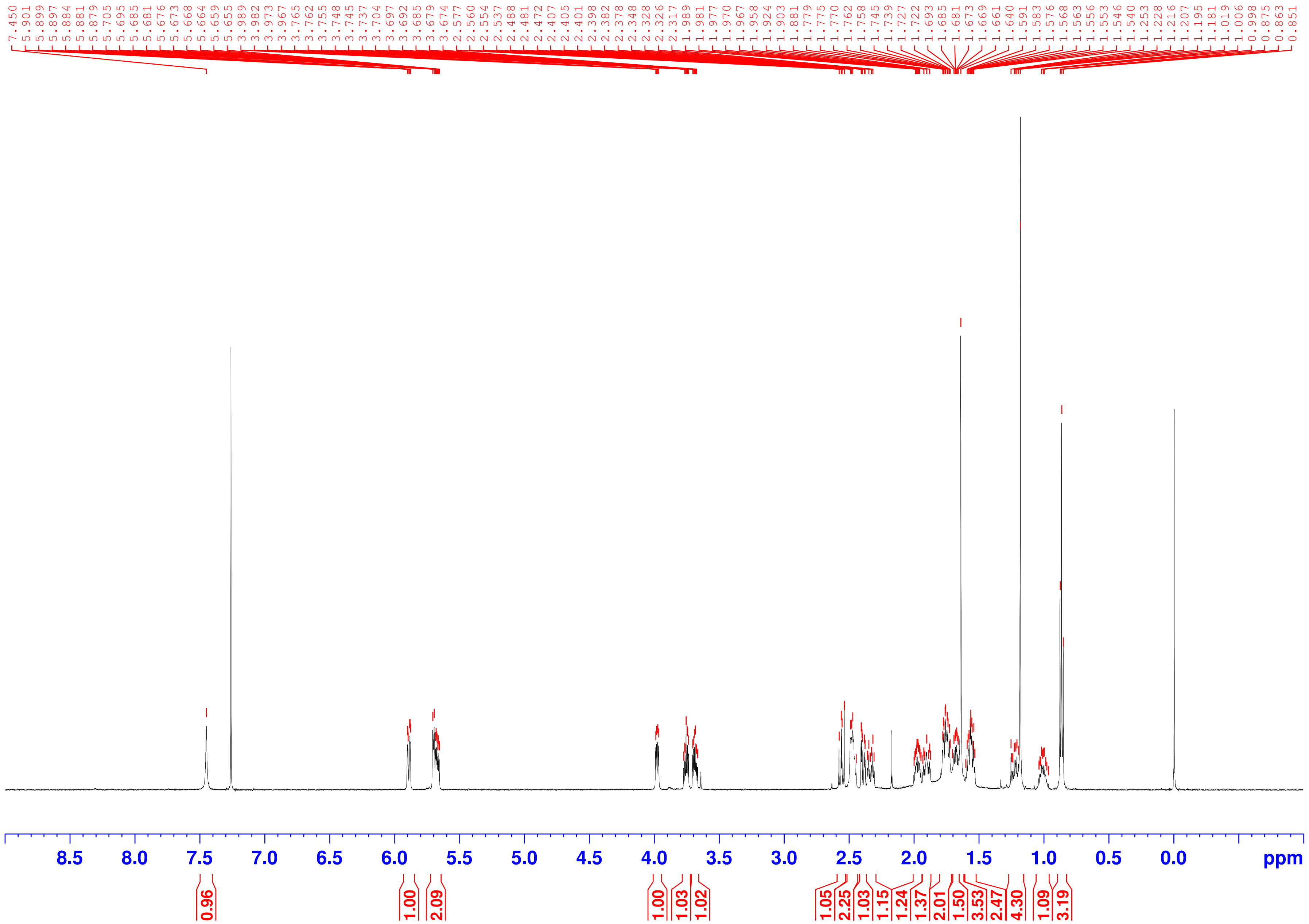

Figure S16. ^{1}H NMR spectrum of **1** in $CDCl_3$ at 600 MHz.

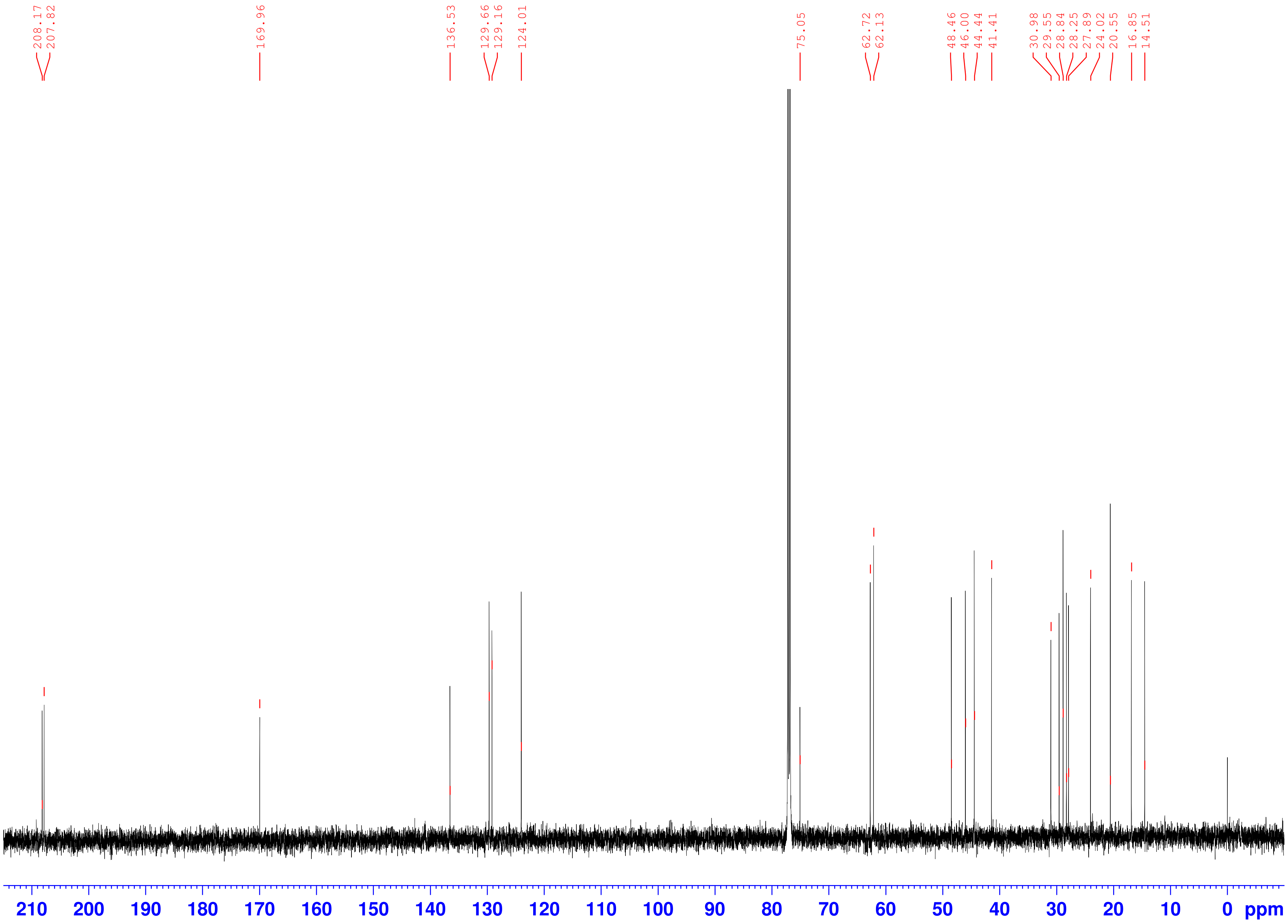

Figure S17. $^{13}C\{^{1}H\}$ NMR spectrum of **1** in $CDCl_3$ at 150 MHz.

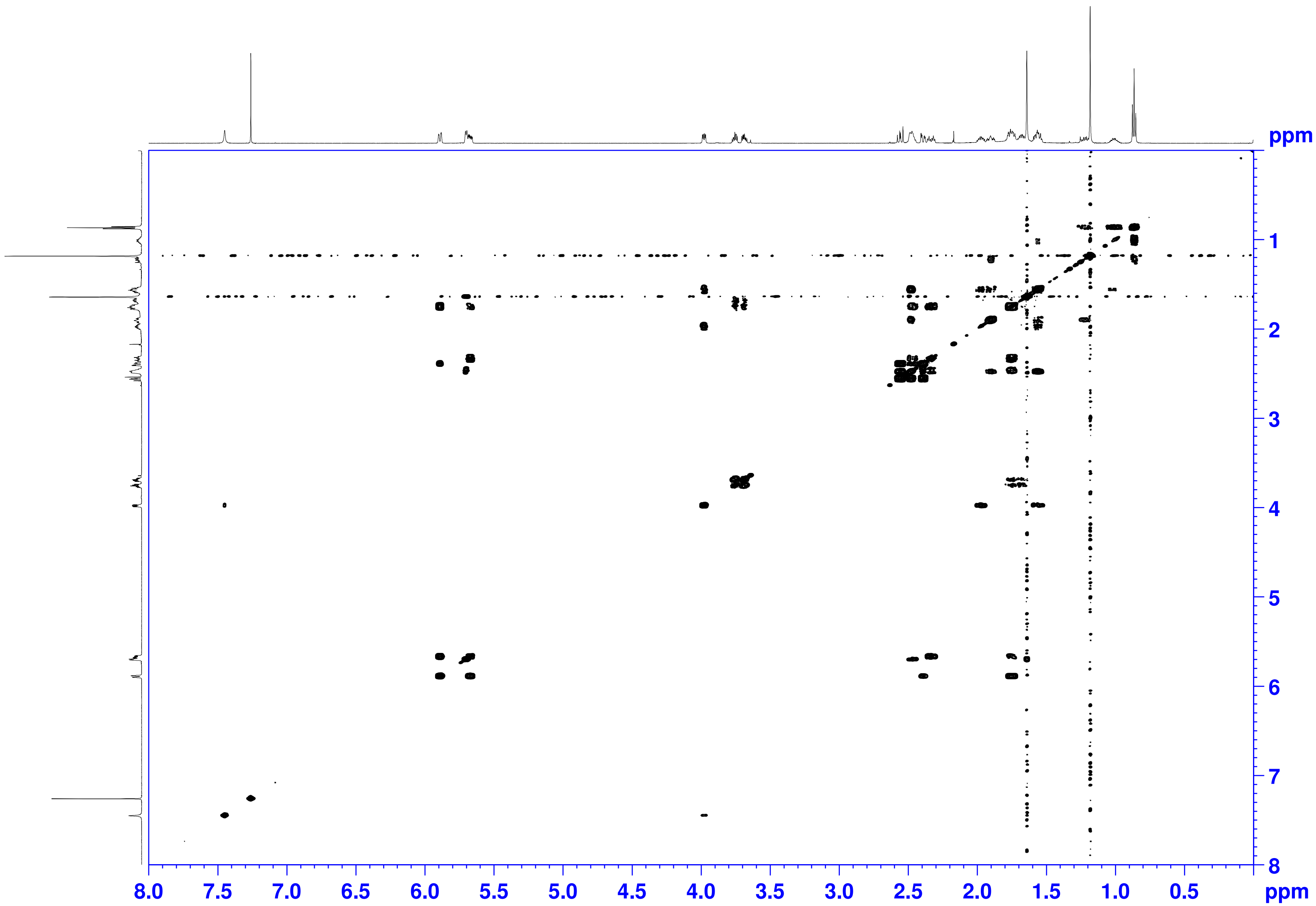

Figure S18. ^{1}H-^{1}H COSY spectrum of **1** in $CDCl_3$.

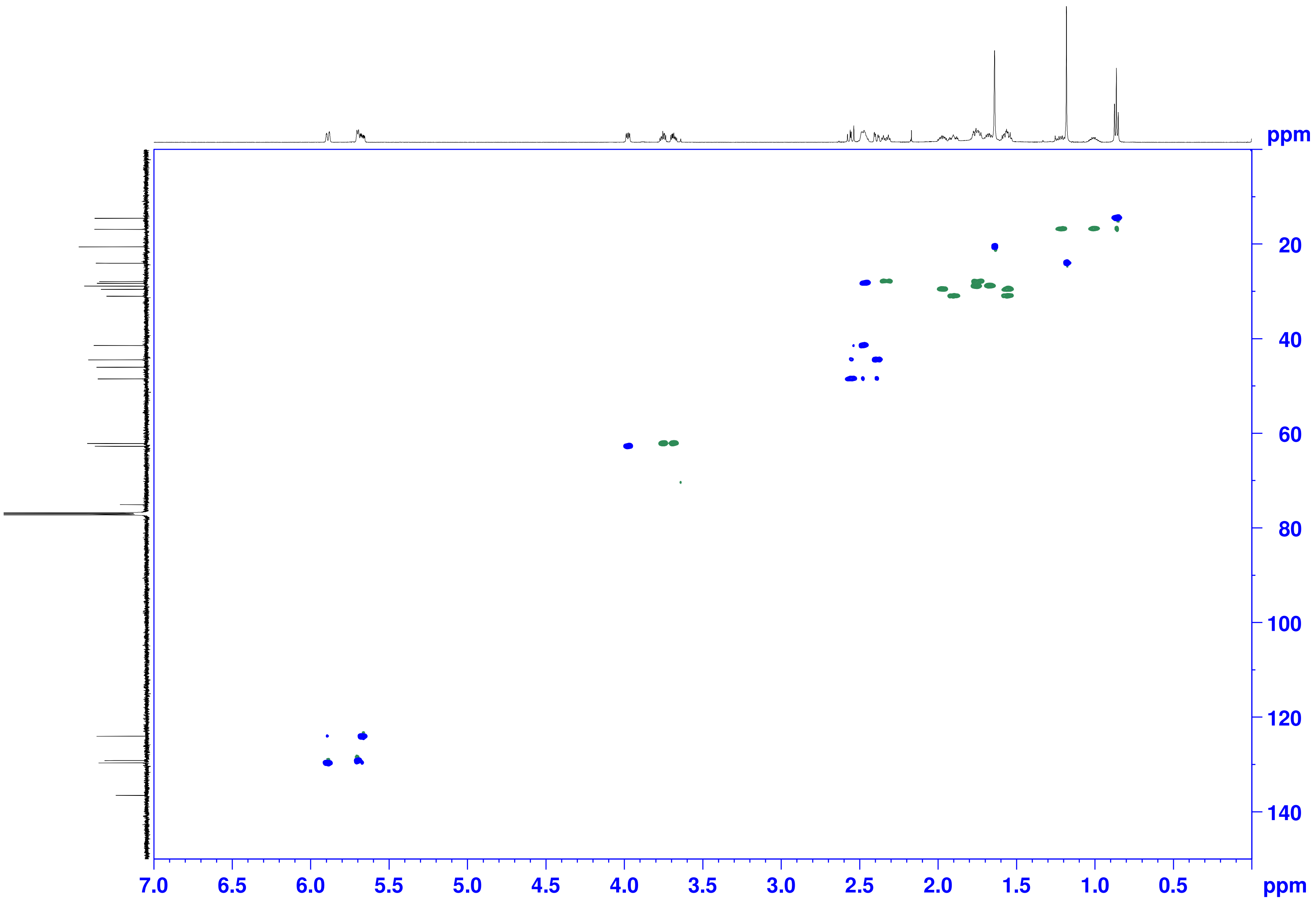

Figure S19. HSQC spectrum of **1** in $CDCl_3$.

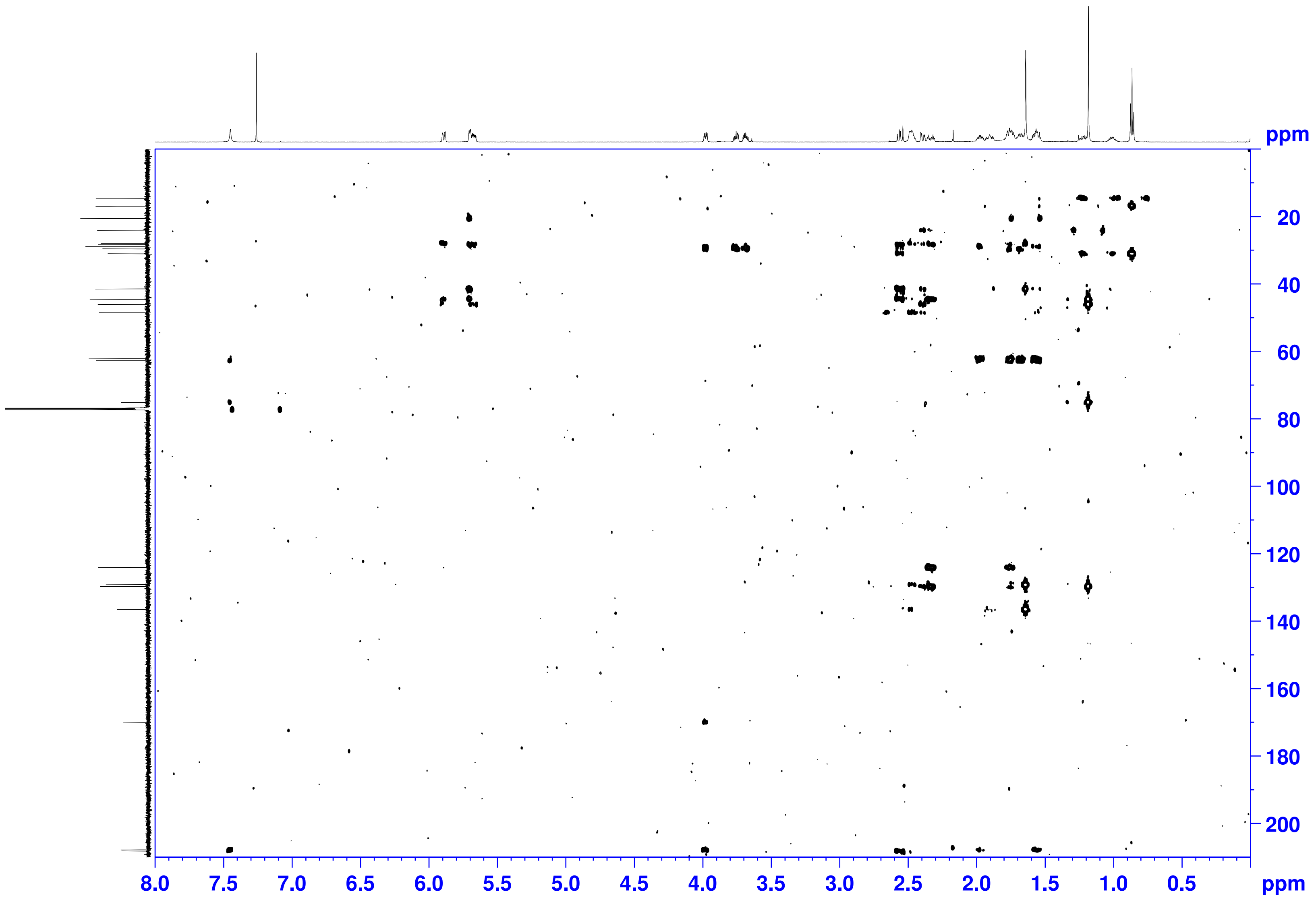

Figure S20. HMBC spectrum of **1** in $CDCl_3$.

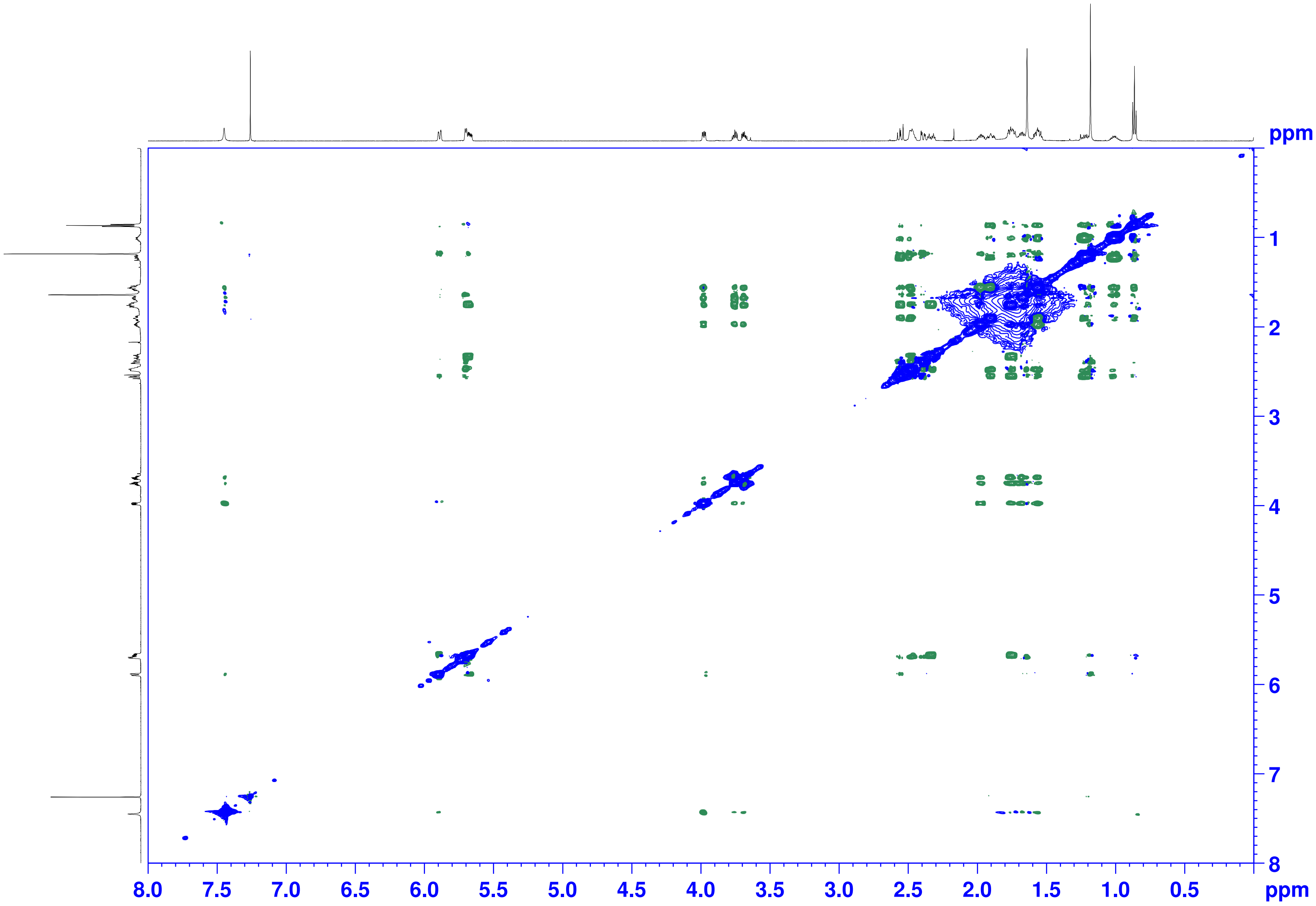

Figure S21. NOESY spectrum of **1** in $CDCl_3$.

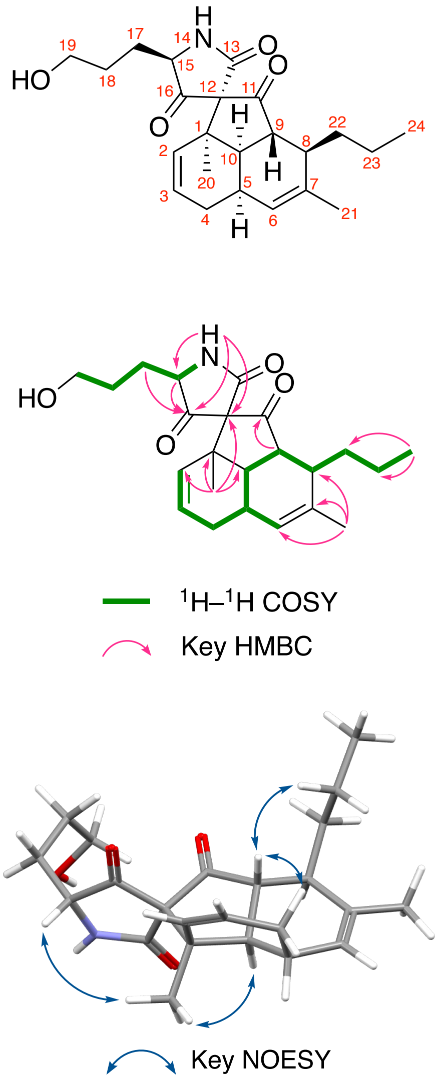

position	δ_C, type	δ_H, mult. (J in Hz)
1	47.0, C	
2	129.04, CH	5.40, dquin (10.4, 1.5)
3	125.4, CH	5.70, ddd (10.4, 5.3, 2.3)
4	28.0, CH_2	2.34 (α), dtd (19.0, 5.5, 1.1)
		1.73 (β), m
5	28.5, CH	2.47, m
6	128.97, CH	5.69, brd (5.5)
7	136.6, C	
8	41.0, CH	2.49, m
9	44.5, CH	2.47, t (12.0)
10	44.5, CH	2.41, brd (12.7)
11	209.8, C	
12	74.9, C	
13	172.2, C	
14	–	8.12, s
15	64.0, CH	3.89, dd (7.5, 4.1)
16	206.9, C	
17	28.7, CH_2	2.04, m
		1.78, m
18	28.3, CH_2	1.73, m
		1.60, m
19	62.3, CH_2	3.69, m
20	24.0, CH_3	1.34, s
21	20.5, CH_3	1.63, s
22	30.9, CH_2	1.89, m
		1.57, m
23	16.9, CH_2	1.21, m
		1.01, m
24	14.5, CH_3	0.87, t (7.3)

^{1}H NMR: 600 MHz, ^{13}C NMR: 150 MHz (in $CDCl_3$)

Figure S22. NMR data of pterrespiramide B (**2**).

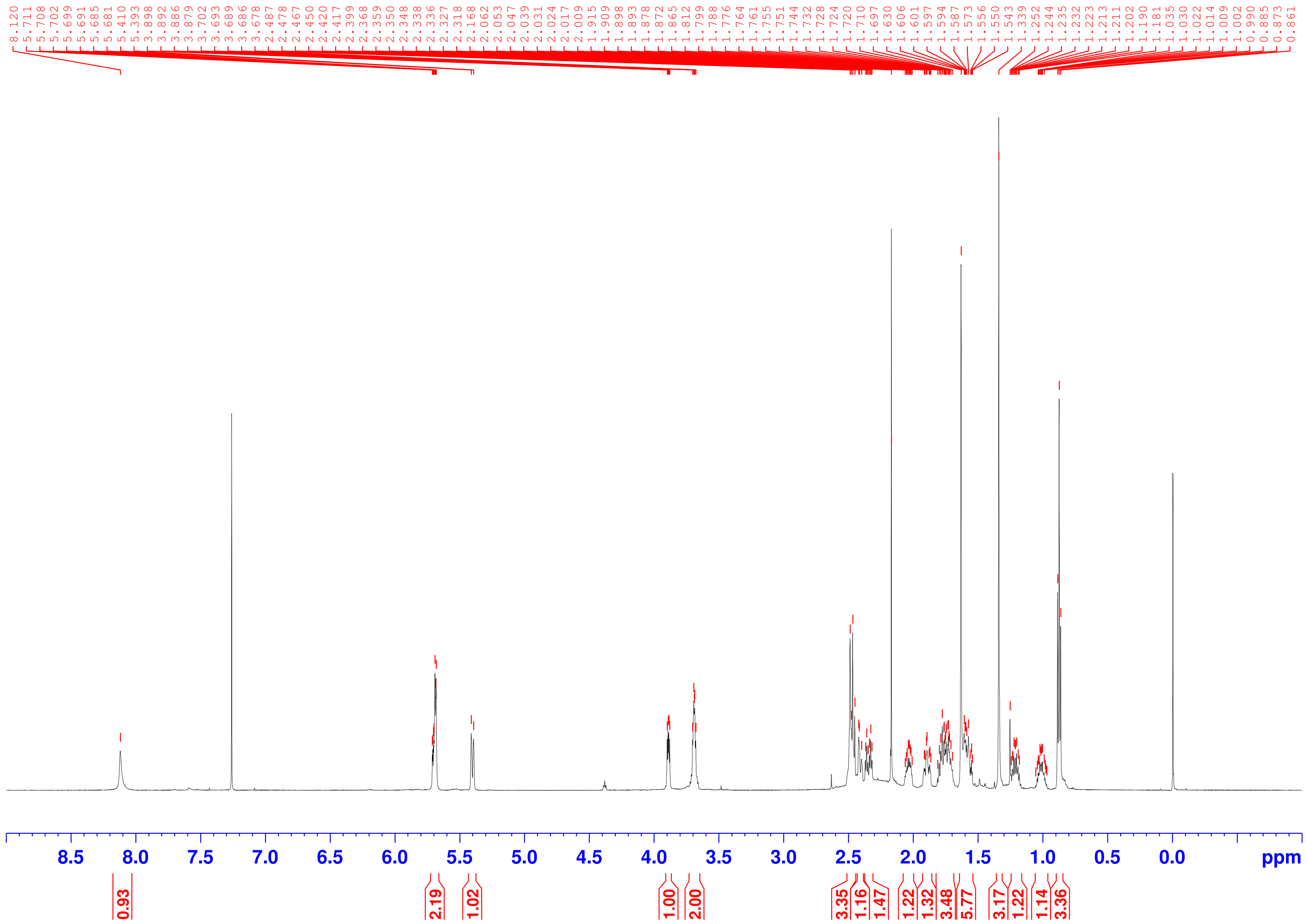

Figure S23. ^{1}H NMR spectrum of **2** in $CDCl_3$ at 600 MHz.

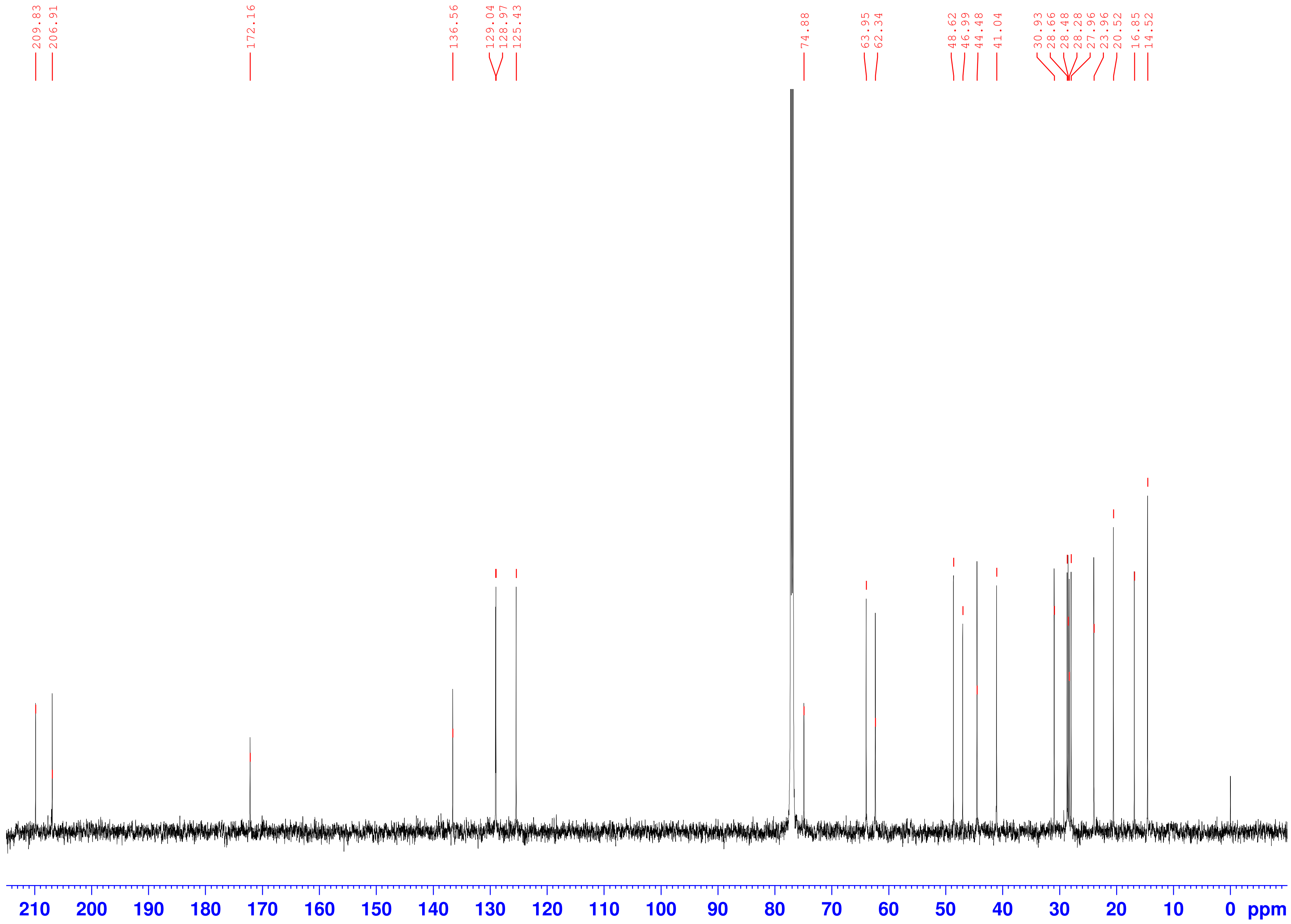

Figure S24. $^{13}C\{^{1}H\}$ NMR spectrum of **2** in $CDCl_3$ at 150 MHz.

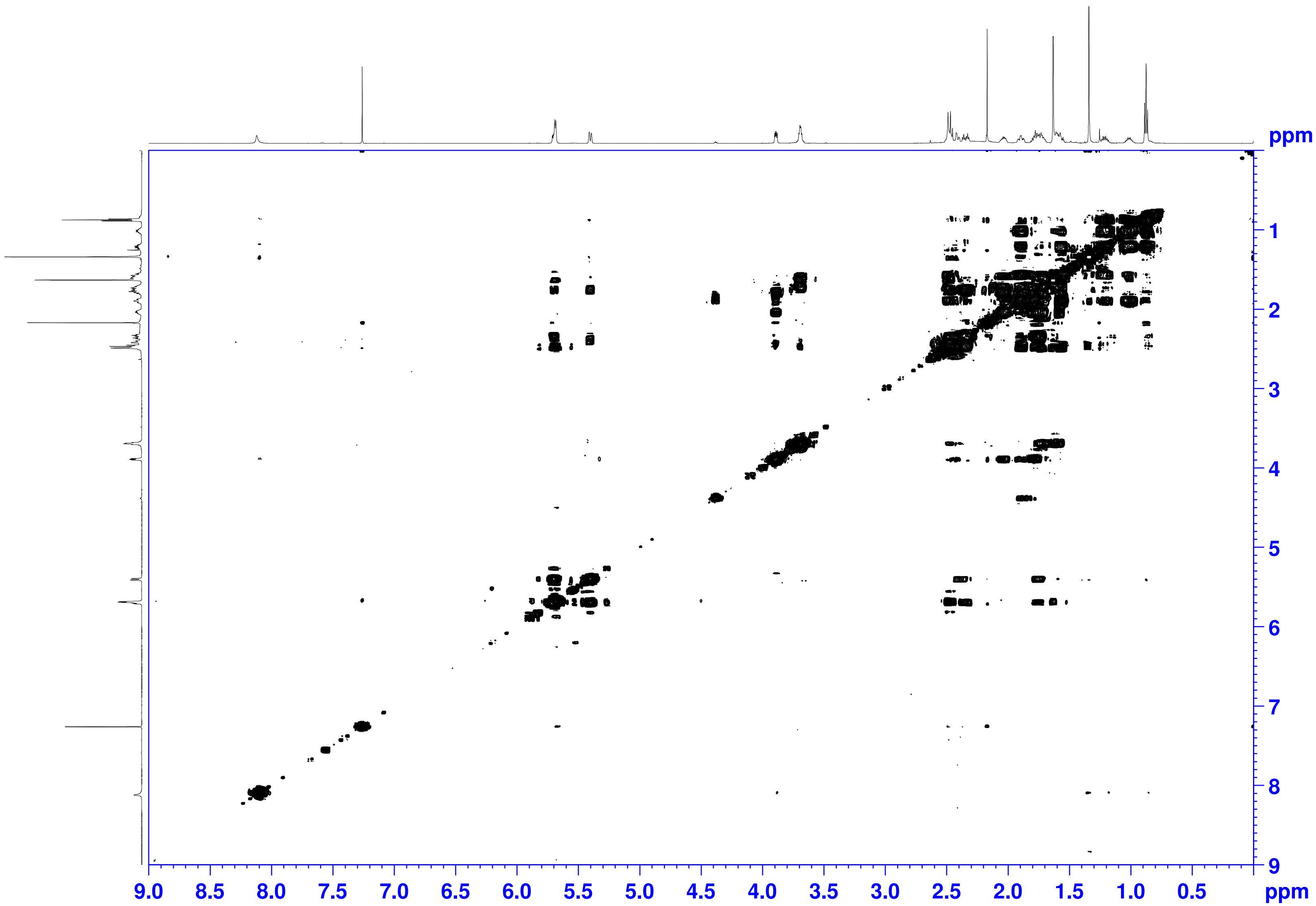

Figure S25. ^{1}H-^{1}H COSY spectrum of **2** in $CDCl_3$.

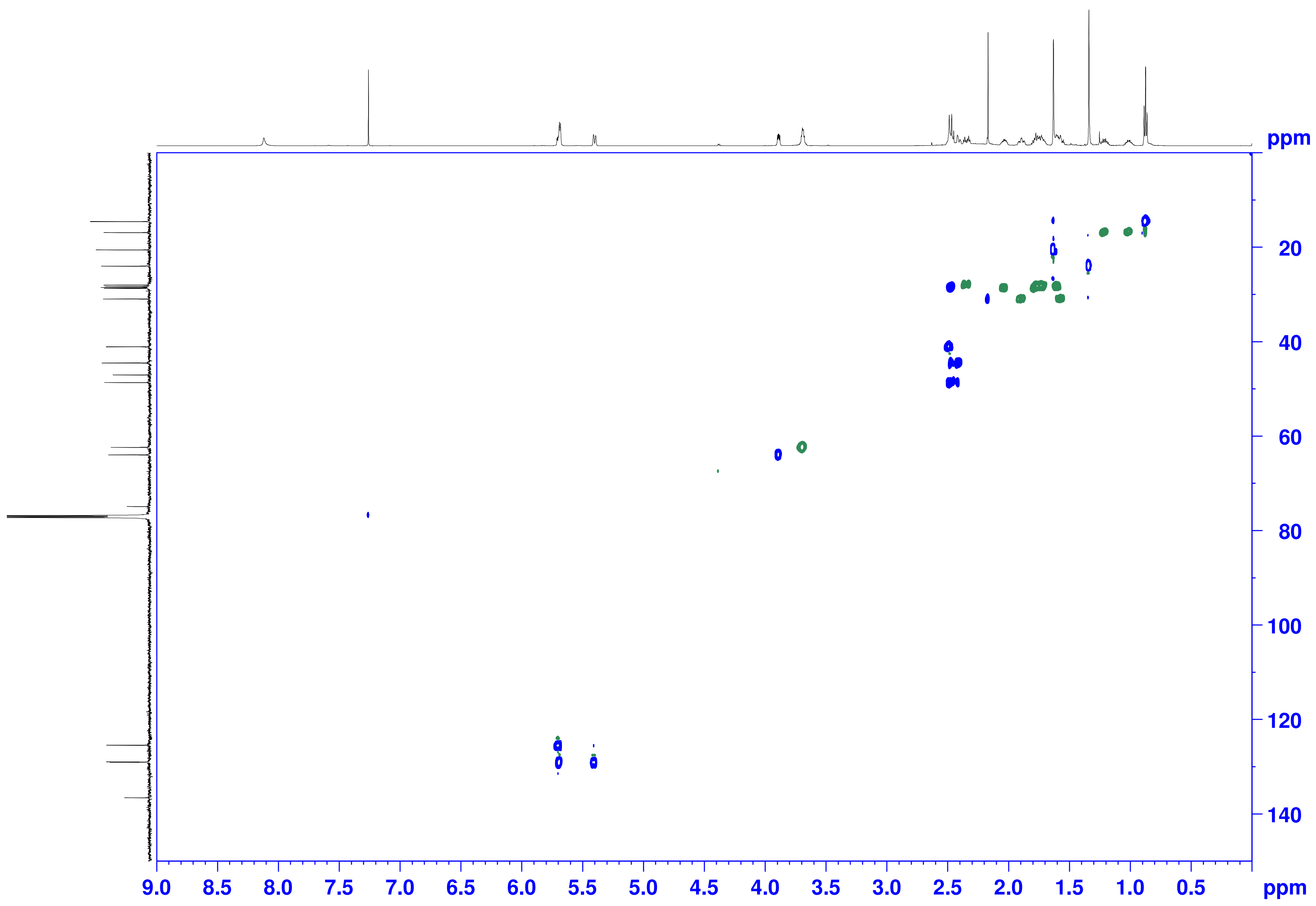

Figure S26. HSQC spectrum of **2** in $CDCl_3$.

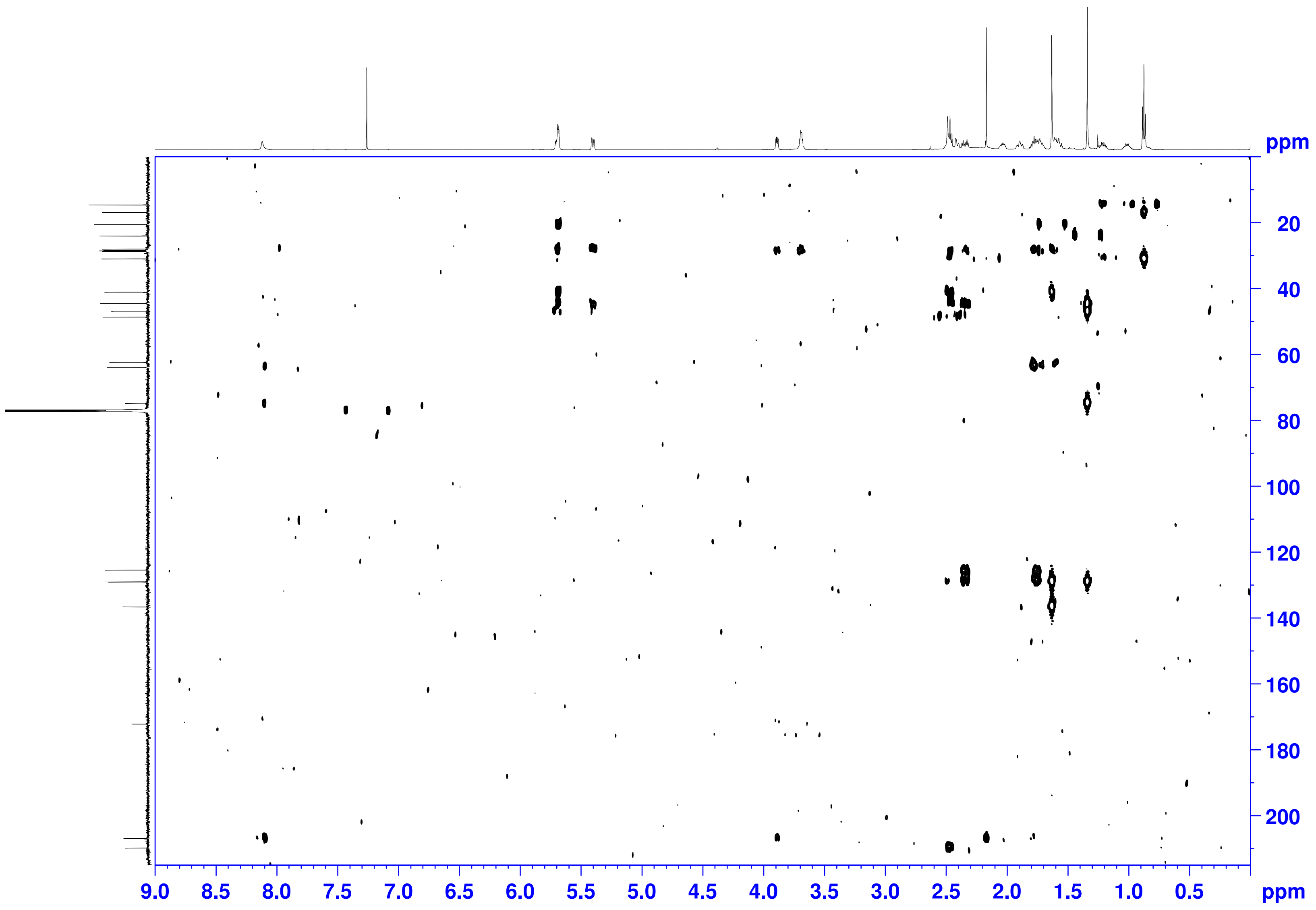

Figure S27. HMBC spectrum of **2** in $CDCl_3$.

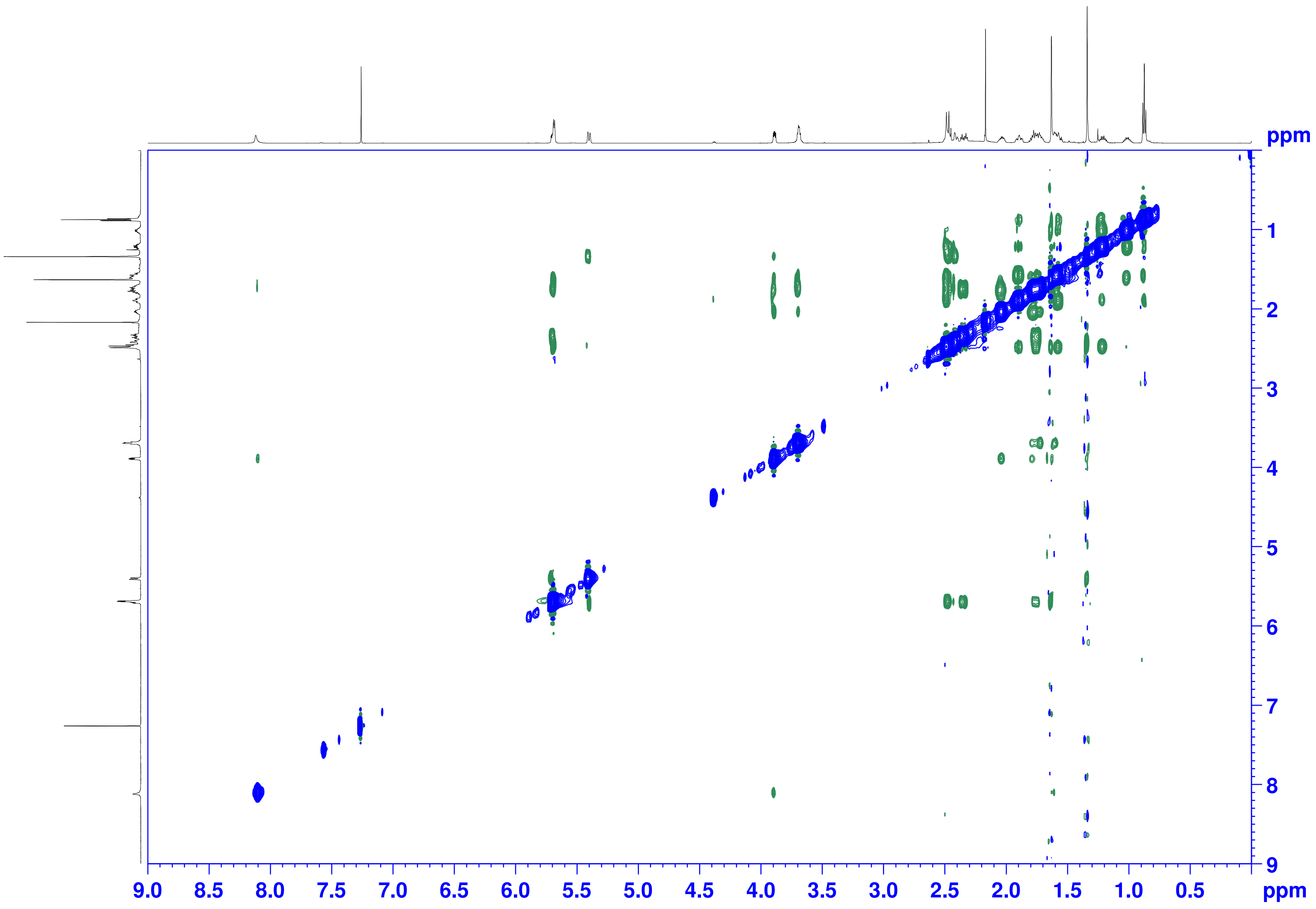

Figure S28. NOESY spectrum of **2** in $CDCl_3$.

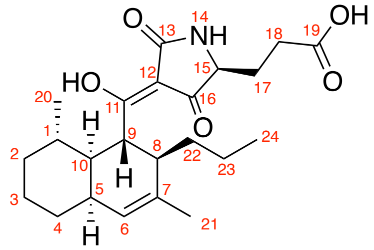

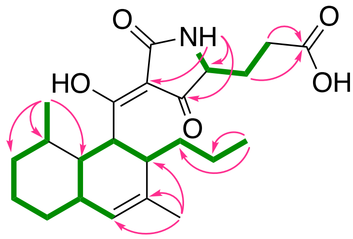

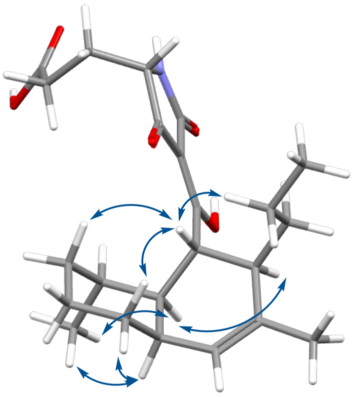

position	δC, type	δH, mult. (*J* in Hz)
1	29.8, CH	1.57, m
2	26.1, CH_2	1.10 (α), m
		1.47 (β), m
3	19.4, CH_2	1.37, m
4	29.4, CH_2	1.57 (α), m
		1.07 (β), m
5	31.1, CH	2.20, m
6	128.8, CH	5.50, d (5.1)
7	133.2, C	
8	41.8, CH	2.43, m
9	39.2, CH	3.98, t (10.7)
10	41.6, CH	1.76, m
11	191.2, C	
12	103.3, C	
13	174.9, C	
14	–	9.05, s
15	60.6, CH	3.93, brt (5.7)
16	195.3, C	
17	26.8, CH_2	1.91, m
		1.68, m
18	28.7, CH_2	2.24, m
19	173.6, C	
20	19.0, CH_3	0.95, d (7.2)
21	20.6, CH_3	1.58, s
22	31,5, CH_2	1.45, m
		1.14, m
23	16.7, CH_2	1.38, m
		0.92, m
24	14.5, CH_3	0.76, t (7.1)
11-OH	–	12.16, brs

^{1}H NMR: 600 MHz, ^{13}C NMR: 150 MHz (in DMSO-d_6)

Figure S29. NMR data of pterramide (**3**).

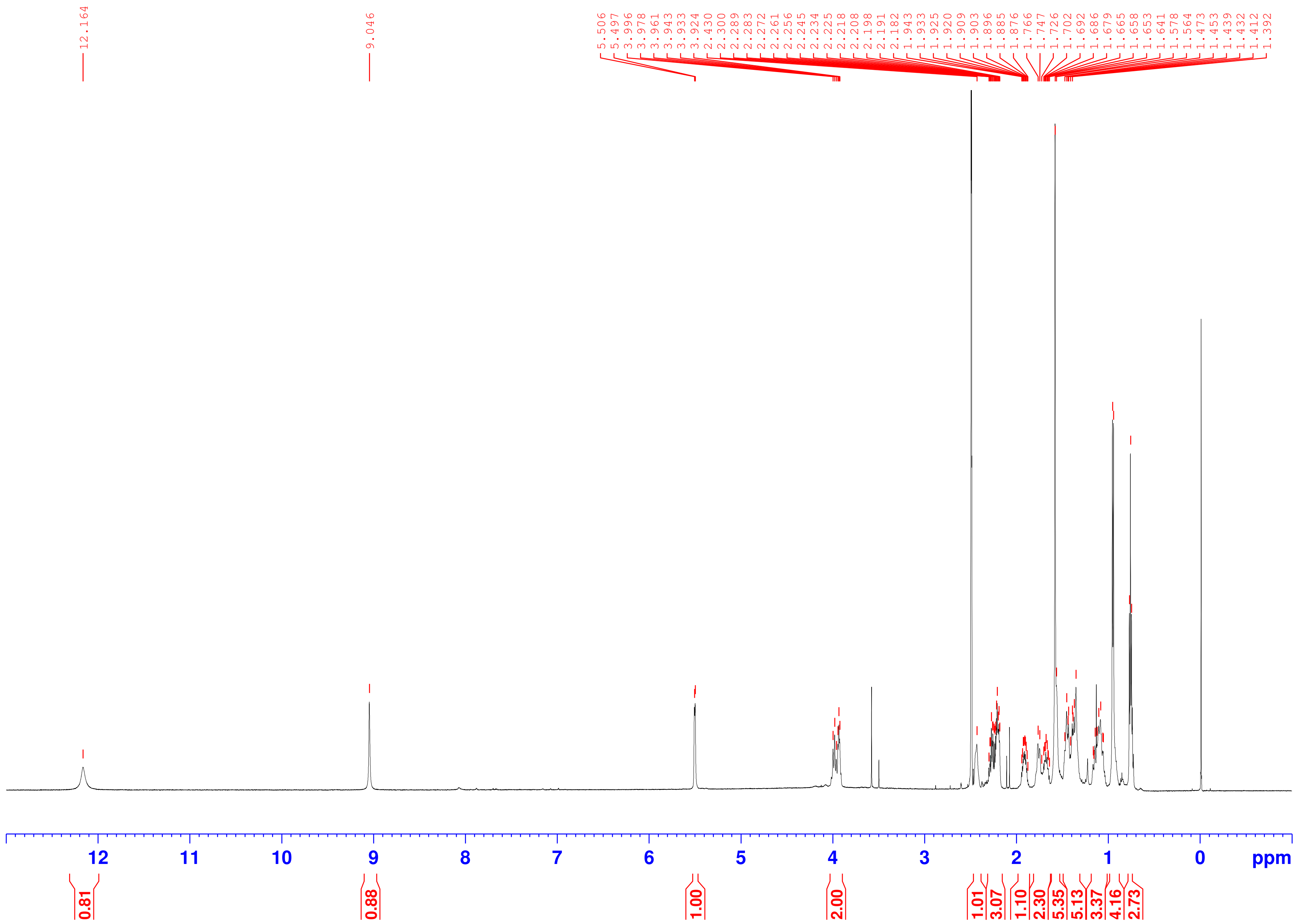

Figure S30. ^{1}H NMR spectrum of **3** in DMSO-d_6 at 600 MHz.

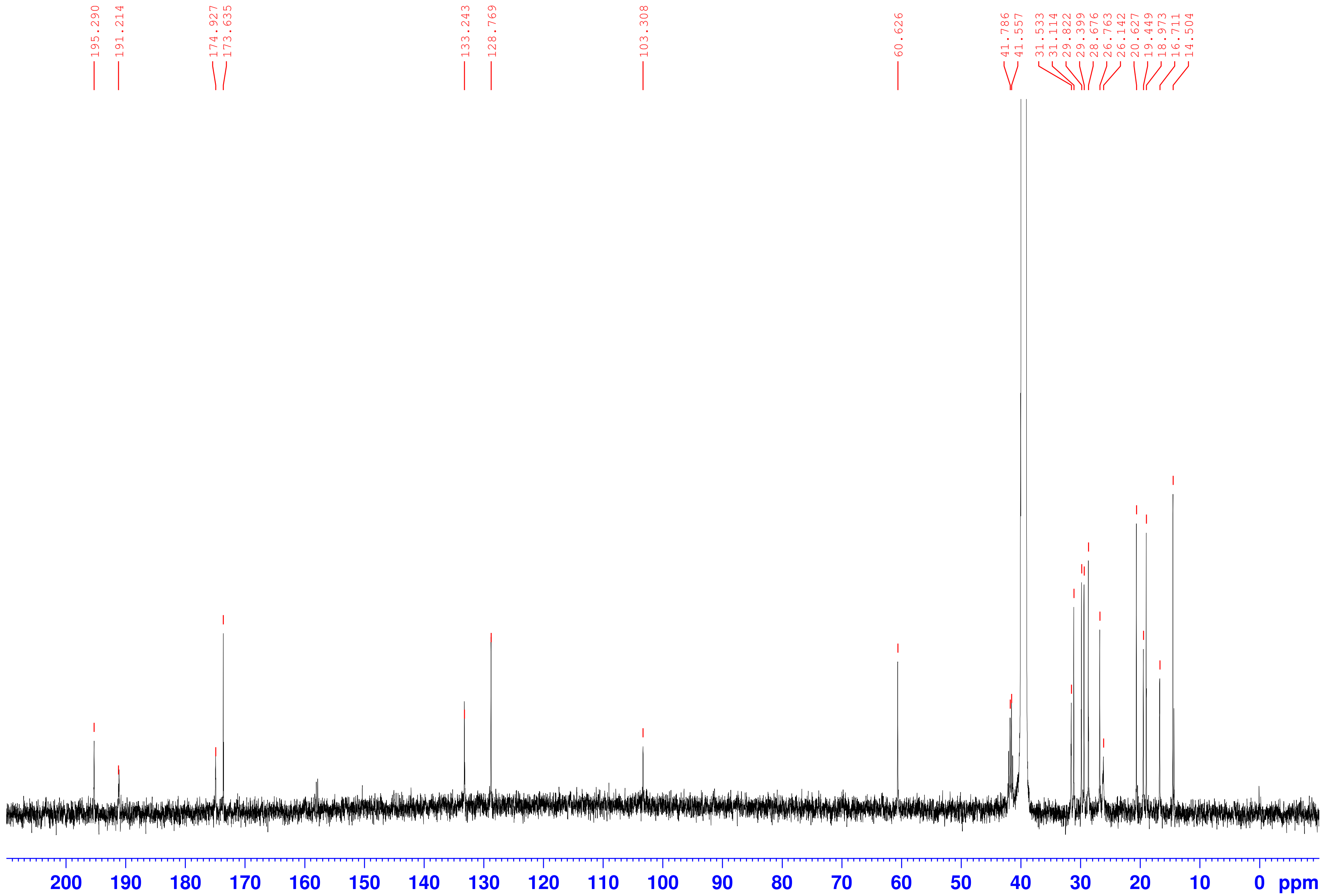

Figure S31. $^{13}C\{^{1}H\}$ NMR spectrum of **3** in DMSO-d_6 at 150 MHz.

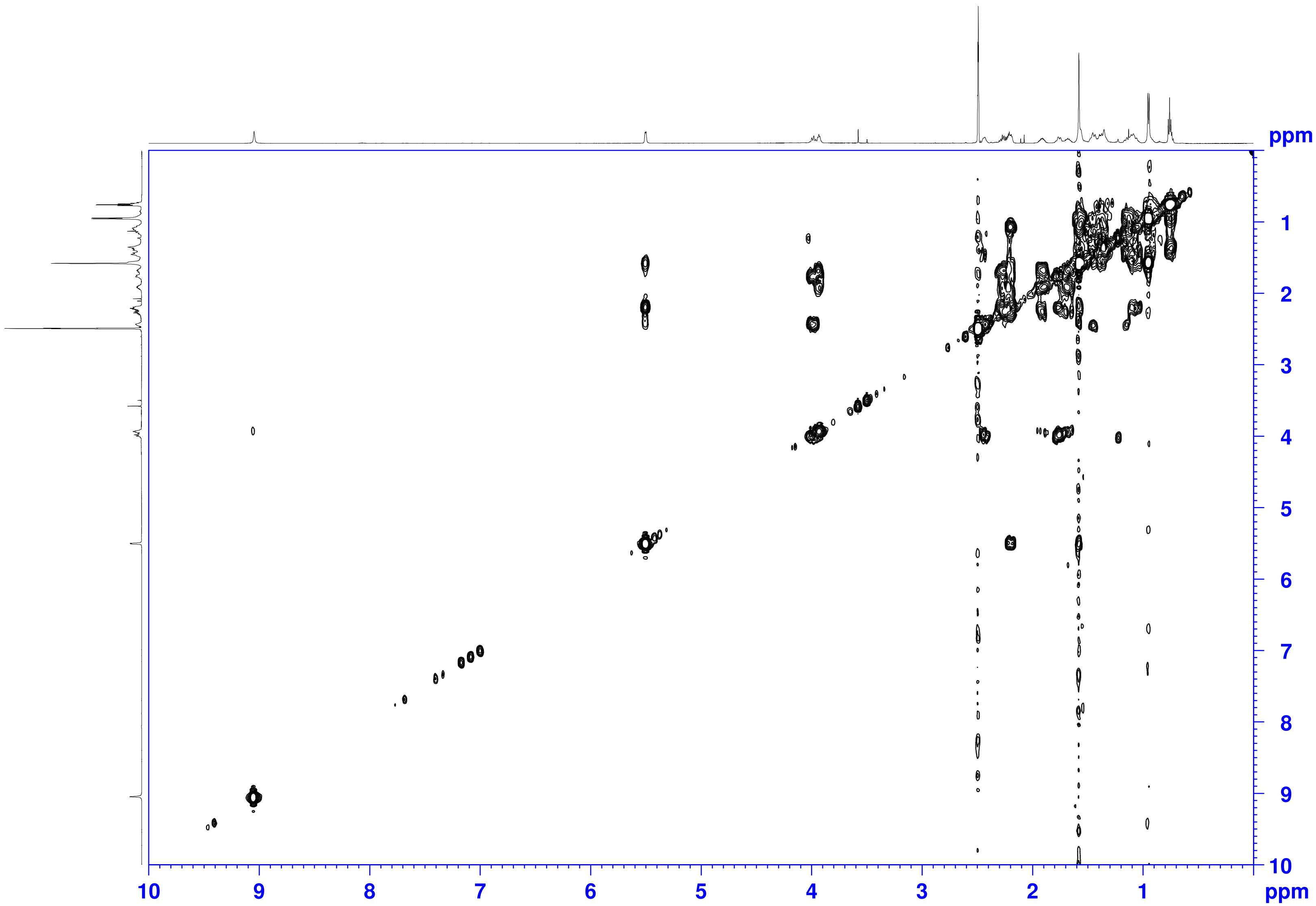

Figure S32. ^{1}H-^{1}H COSY spectrum of **3** in DMSO-d_6.

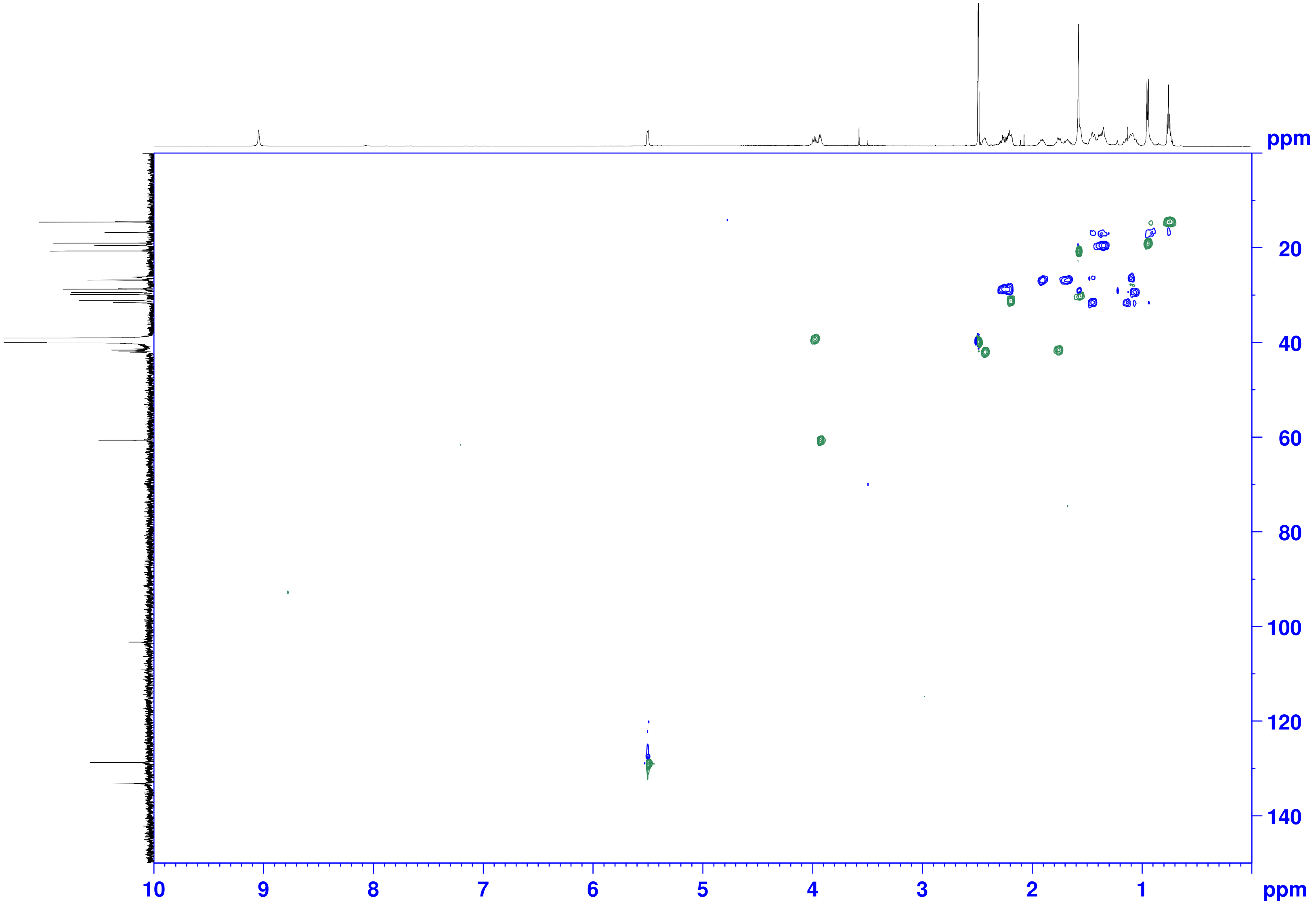

Figure S33. HSQC spectrum of **3** in DMSO-d_6.

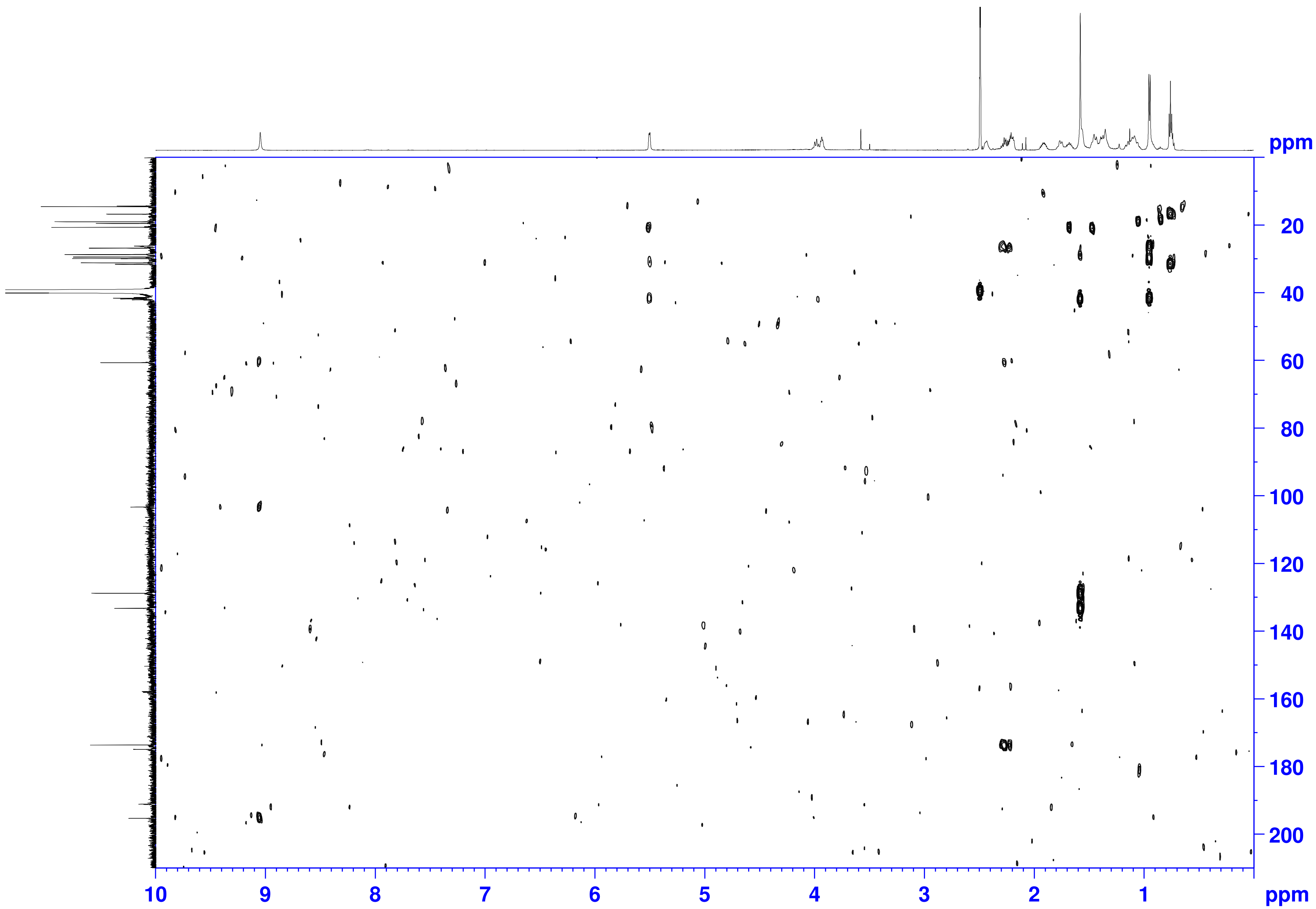

Figure S34. HMBC spectrum of **3** in DMSO-d_6.

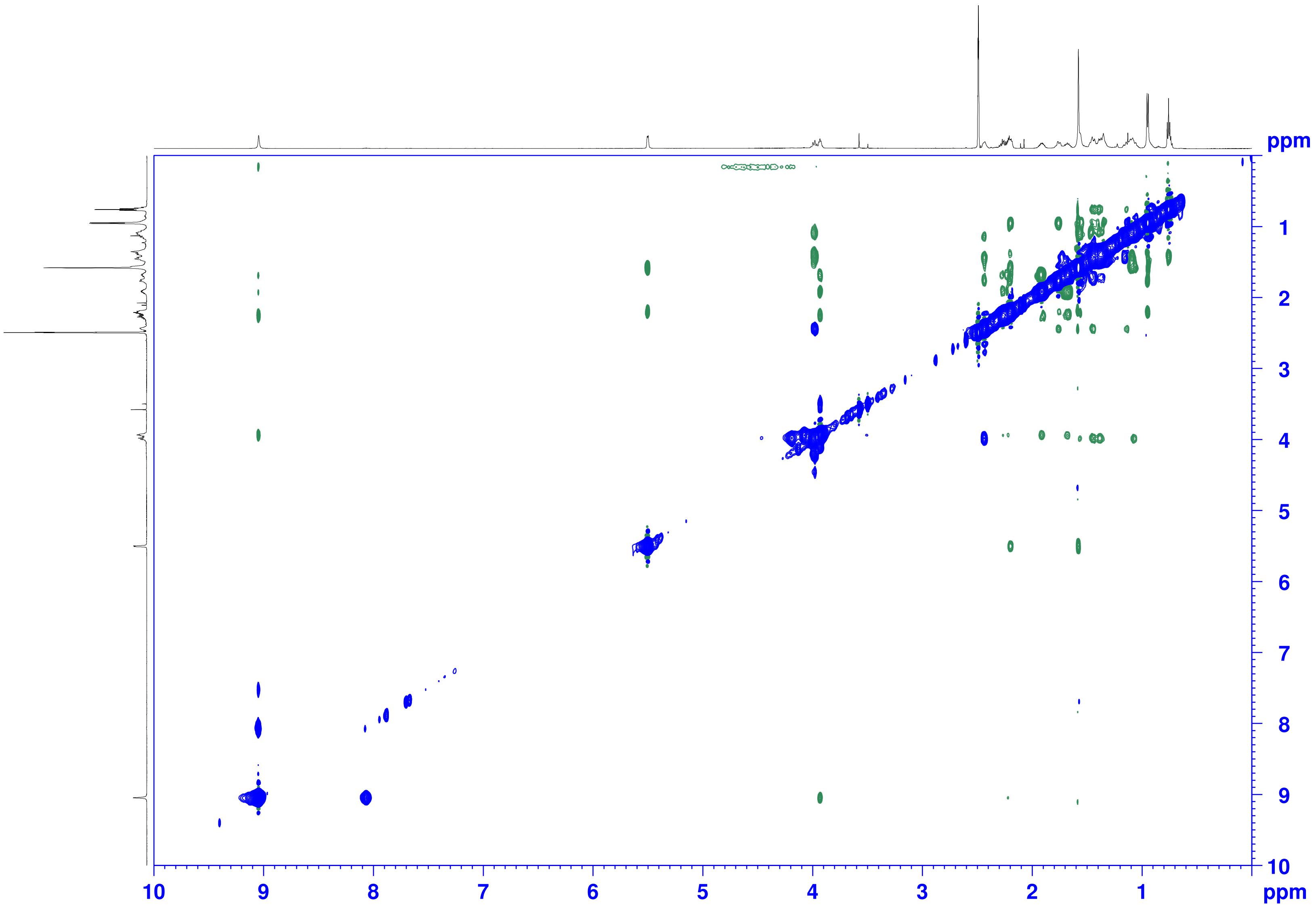

Figure S35. NOESY spectrum of **3** in DMSO-d_6.

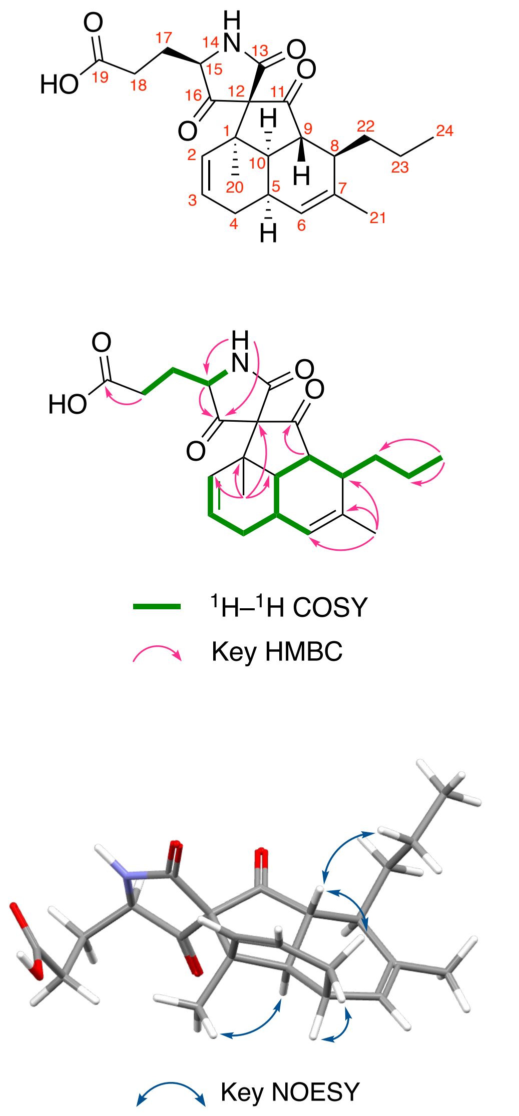

position	δ_C, type	δ_H, mult. (J in Hz)
1	46.4, C	
2	129.4, CH	5.93, dquin (10.4, 1.4)
3	124.1, CH	5.65, ddd (10.4, 5.2, 2.2)
4	27.9, CH_2	2.34 (α), dtd (18.9, 5.5, 1.3)
		1.74 (β), ddt (19.0, 9.8, 2.5)
5	28.1, CH	2.48, m
6	129.2, CH	5.71, brd (5.9)
7	136.5, C	
8	41.6, CH	2.49, m
9	48.4, CH	2.53, dd (13.4, 10.2)
10	44.3, CH	2.41, ddd (13.9, 4.1, 1.1)
11	207.4, C	
12	75.1, C	
13	172.4, C	
14	–	9.49, s
15	64.4, CH	3.94, dd (6.8, 2.0)
16	207.5, C	
17	26.9, CH_2	2.21, m
		1.89, m
18	32.1, CH_2	2.59, ddd (18.0, 6.4, 2.9)
		2.52, m
19	177.6, C	
20	23.6, CH_3	1.19, s
21	20.5, CH_3	1.64, s
22	30.9, CH_2	1.92, m
		1.57, ddt (14.1, 12.5, 4.0)
23	16.8, CH_2	1.22, m
		1.01, m
24	14.5, CH_3	0.87, t (7.3)

^{1}H NMR: 600 MHz, ^{13}C NMR: 150 MHz (in $CDCl_3$)

Figure S36. NMR data of pterrespiramide C (**4**).

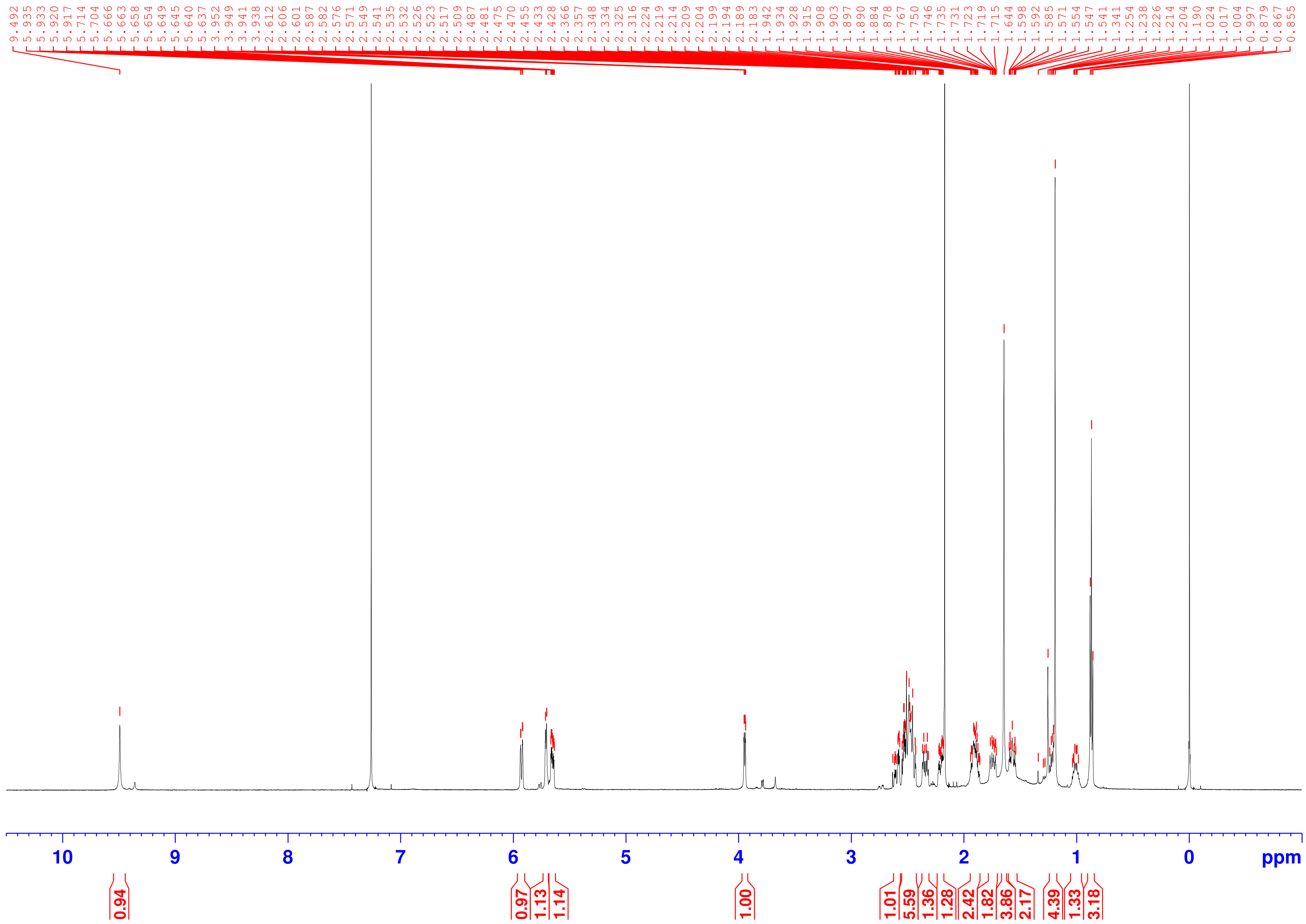

Figure S37. ^{1}H NMR spectrum of **4** in $CDCl_3$ at 600 MHz.

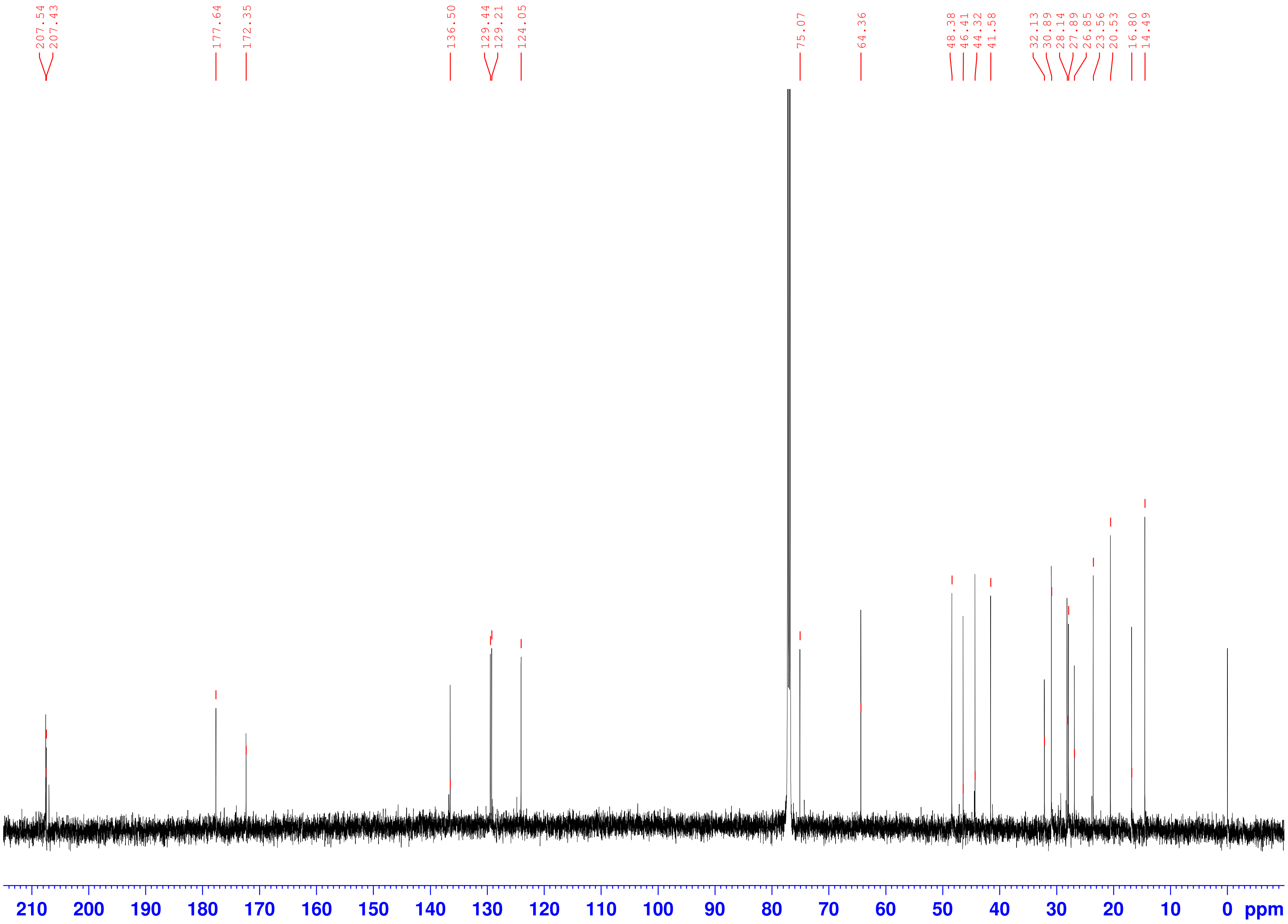

Figure S38. $^{13}C\{^{1}H\}$ NMR spectrum of **4** in $CDCl_3$ at 150 MHz.

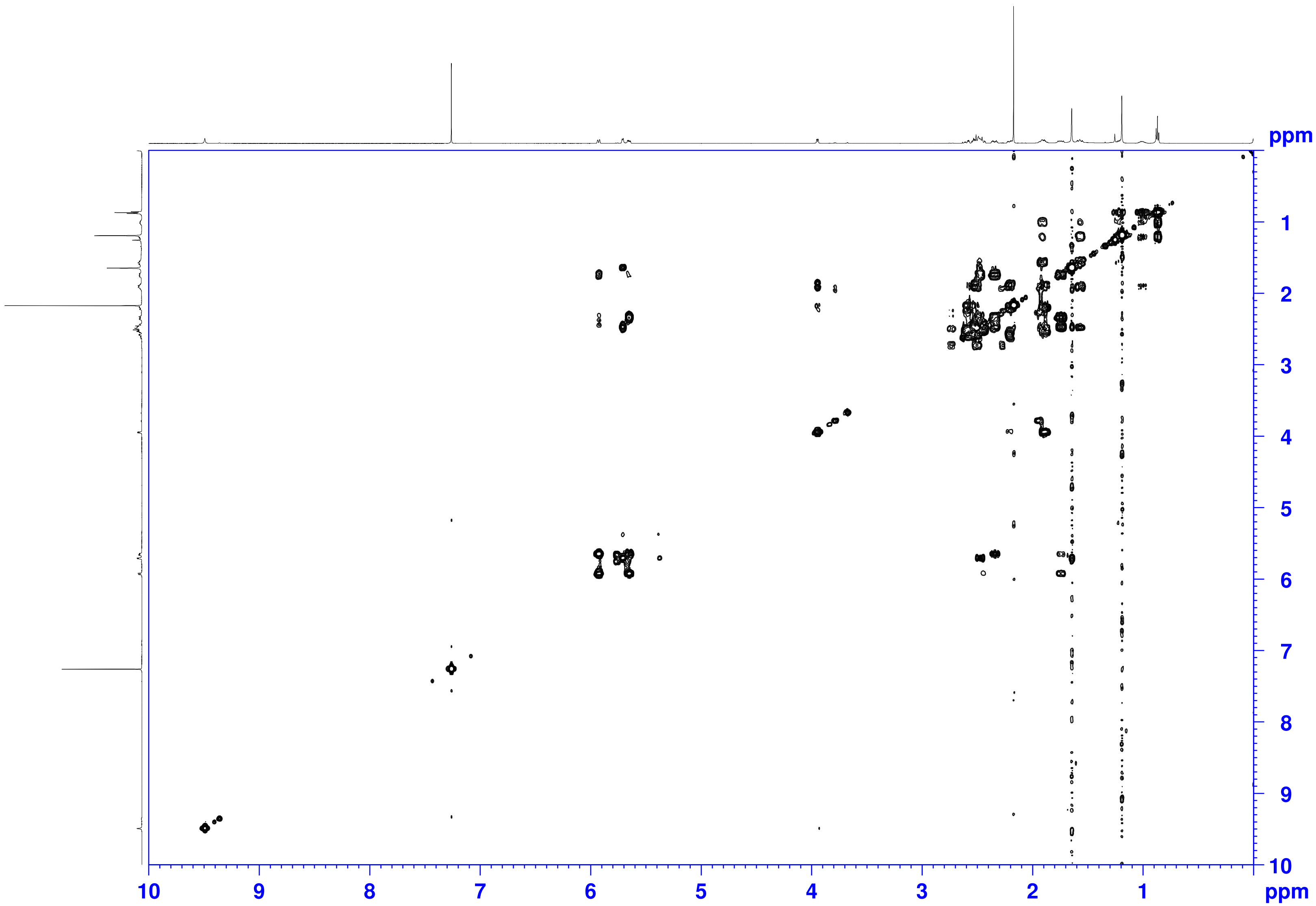

Figure S39. ^{1}H-^{1}H COSY spectrum of **4** in $CDCl_3$.

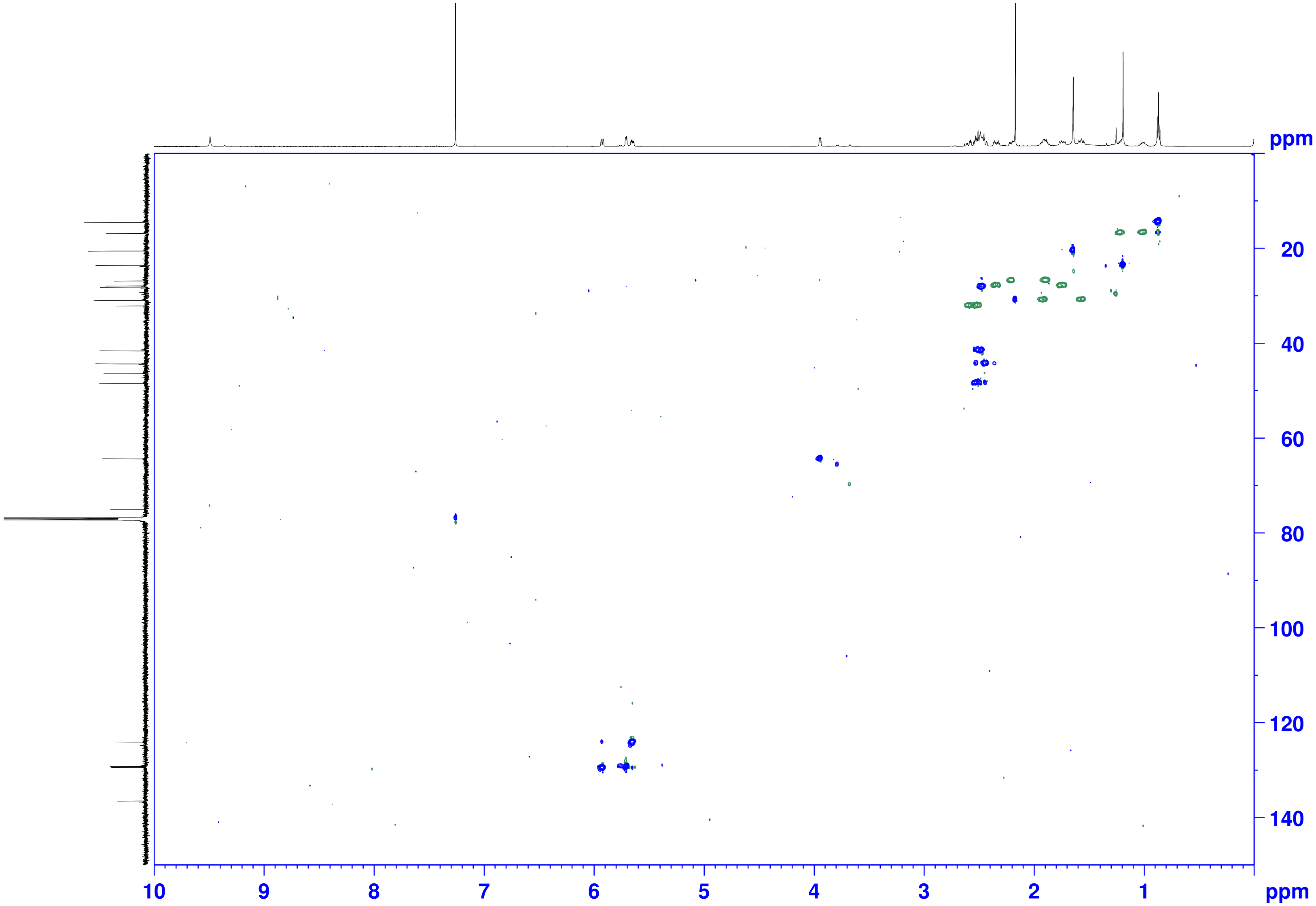

Figure S40. HSQC spectrum of **4** in $CDCl_3$.

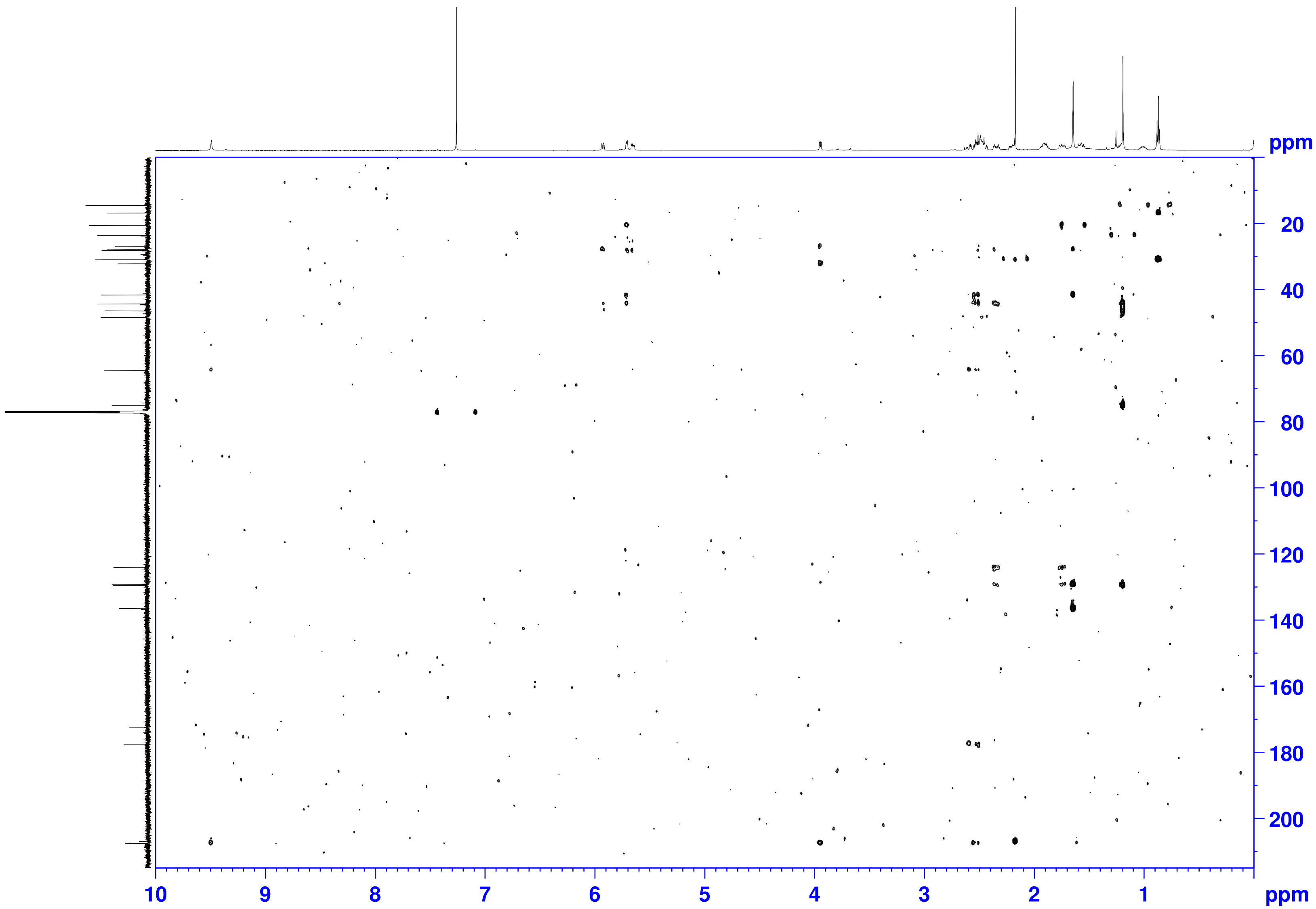

Figure S41. HMBC spectrum of **4** in $CDCl_3$.

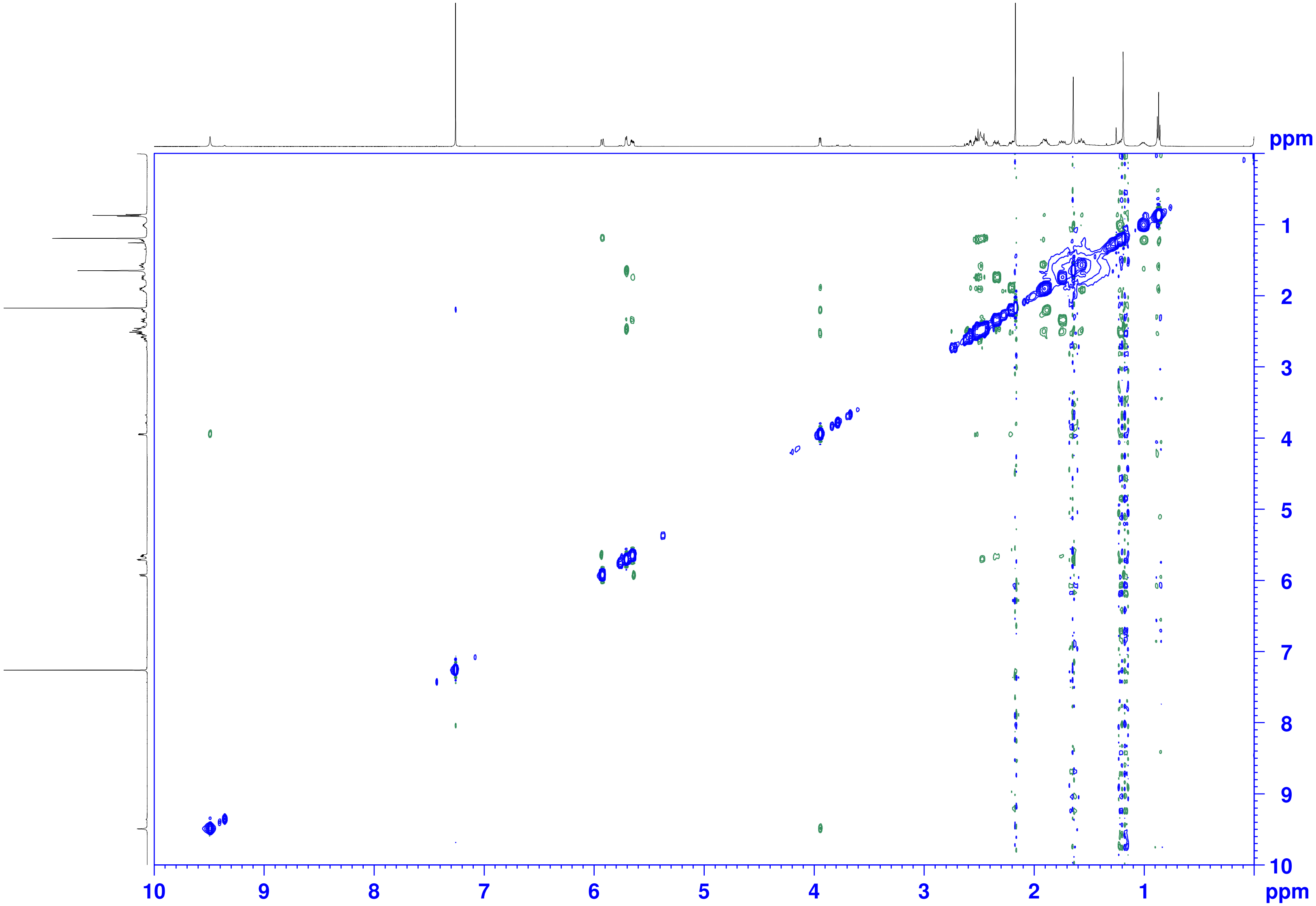

Figure S42. NOESY spectrum of **4** in $CDCl_3$.

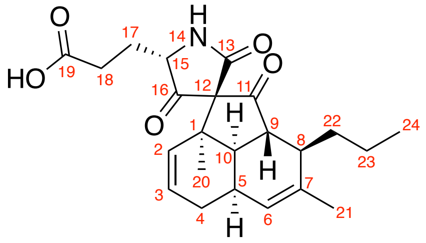

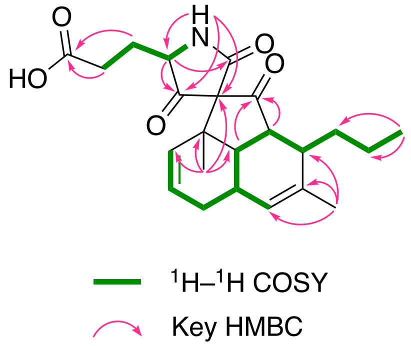

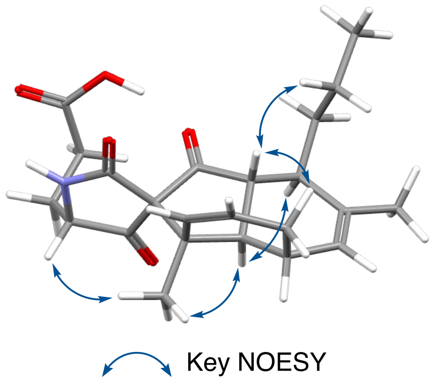

position	δC, type	δH, mult. (*J* in Hz)
1	47.2, C	
2	129.1, CH	5.74, brd (10.4)
3	124.8, CH	5.66, ddd (10.4, 5.0, 2.0)
4	28.0, CH_2	2.34 (α), dt (18.5, 5.0)
		1.74 (β), ddt (19.0,10.0, 2.4)
5	28.3, CH	2.47, m
6	128.9, CH	5.70, brd (6.1)
7	136.7, C	
8	41.2, CH	2.51, m
9	48.4, CH	2.48, t (11.7)
10	44.9, CH	2.34, dd (12.3, 4.1)
11	208.6, C	
12	74.1, C	
13	172.4, C	
14	–	9.36, brs
15	64.7, CH	3.89, dd (5.2, 3.7)
16	208.9, C	
17	25.4, CH_2	2.34, m
		2.04, m
18	30.7, CH_2	2.63, ddd (18.3, 8.2, 2.1)
		2.46, m
19	178.0, C	
20	23.8, CH_3	1.18, s
21	20.5, CH_3	1.64, s
22	30.9, CH_2	1.93, m
		1.56, m
23	16.8, CH_2	1.22, m
		1.00, m
24	14.5, CH_3	0.87, t (7.2)

^{1}H NMR: 600 MHz, ^{13}C NMR: 150 MHz (in $CDCl_3$)

Figure S43. NMR data of pterrespiramide D (**5**).

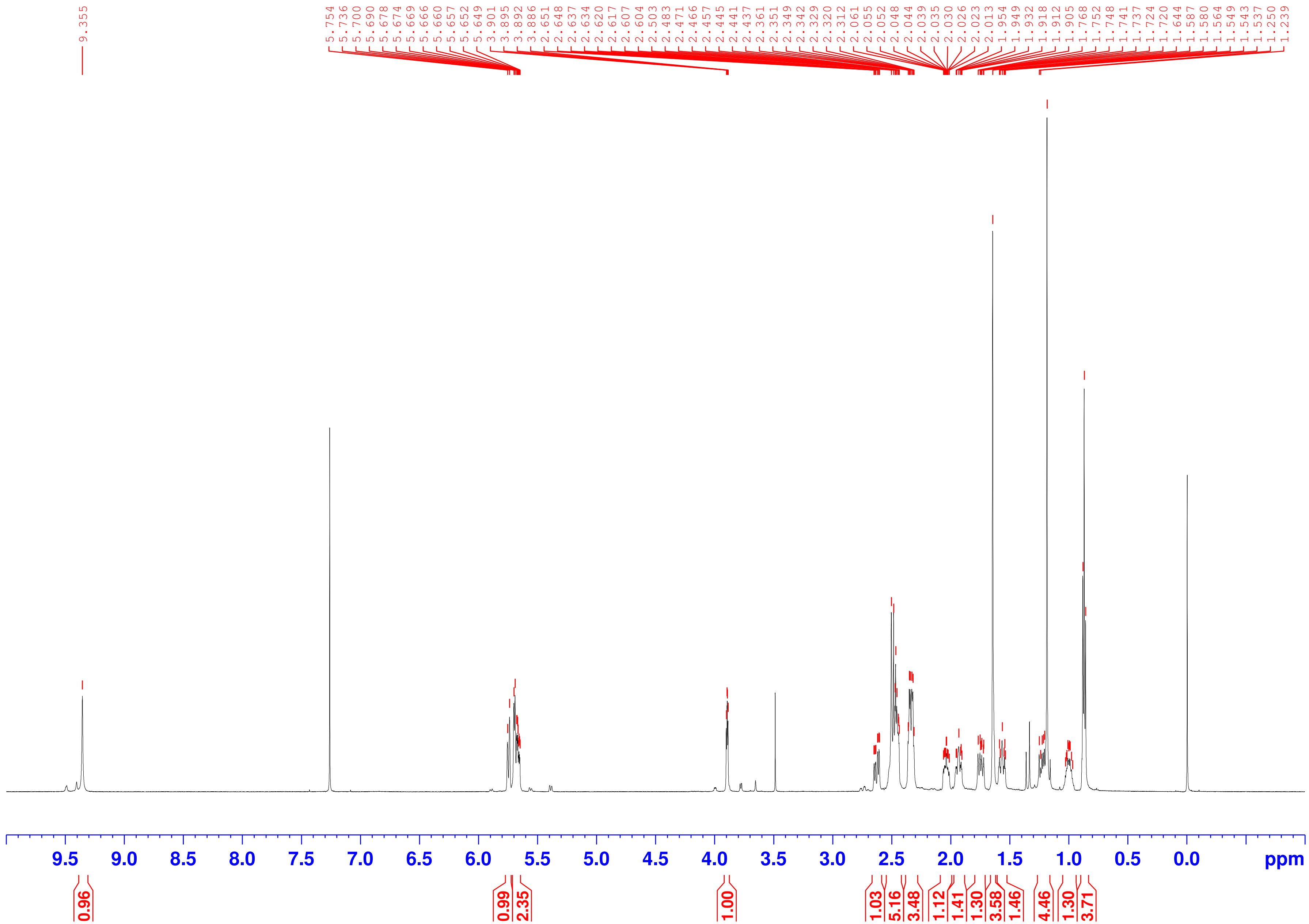

Figure S44. ^{1}H NMR spectrum of **5** in $CDCl_3$ at 600 MHz.

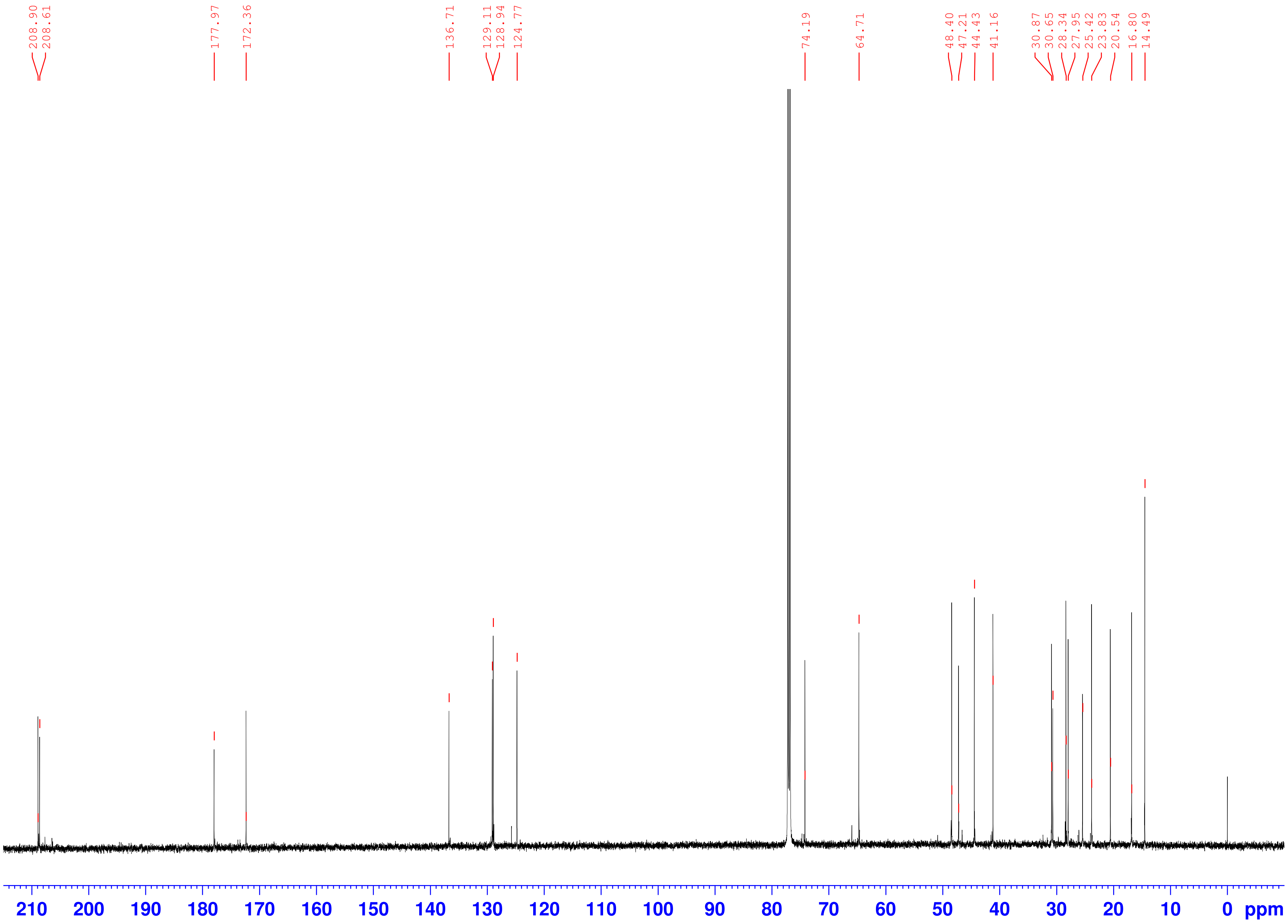

Figure S45. $^{13}C\{^{1}H\}$ NMR spectrum of **5** in $CDCl_3$ at 150 MHz.

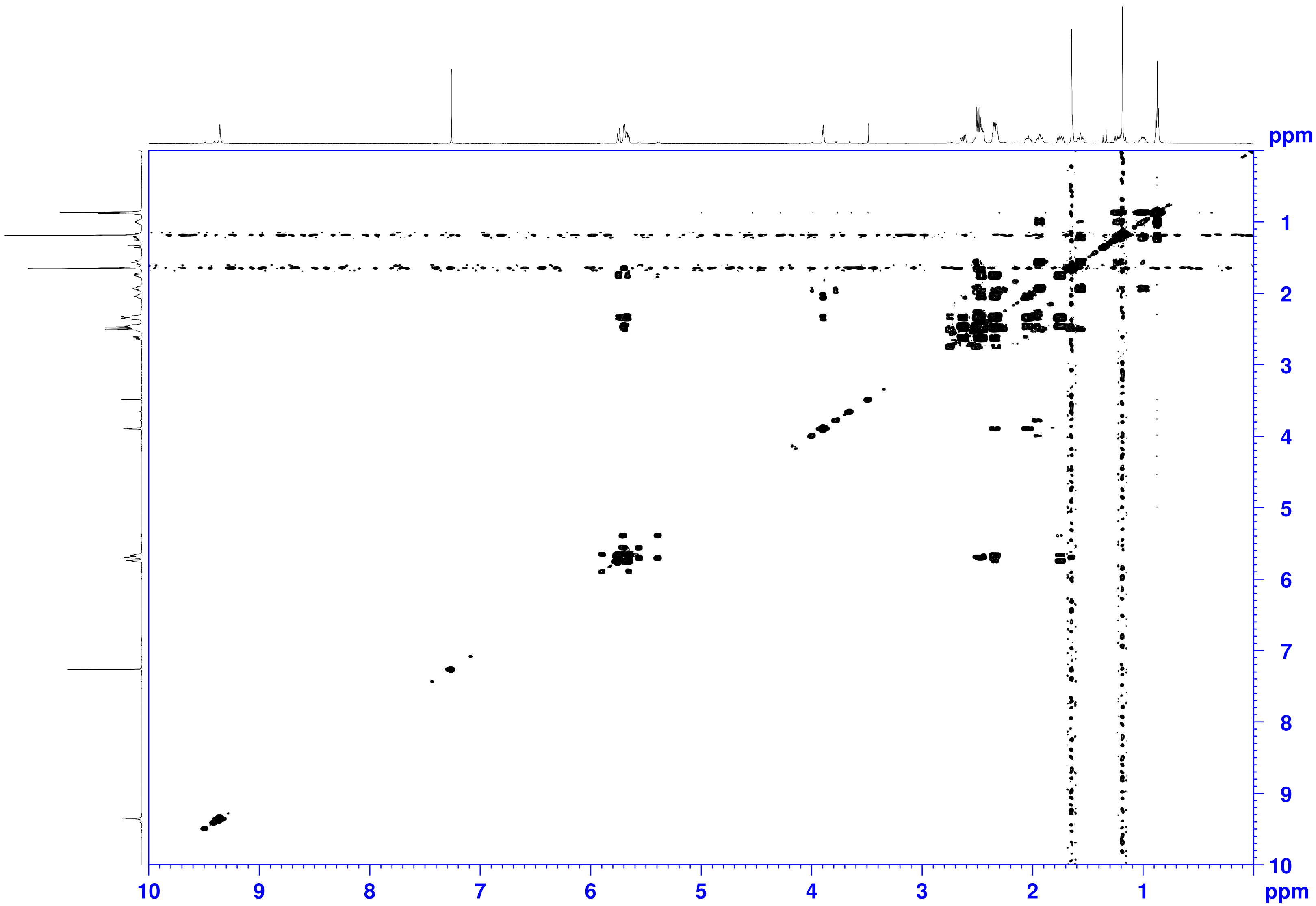

Figure S46. ^{1}H-^{1}H COSY spectrum of **5** in $CDCl_3$.

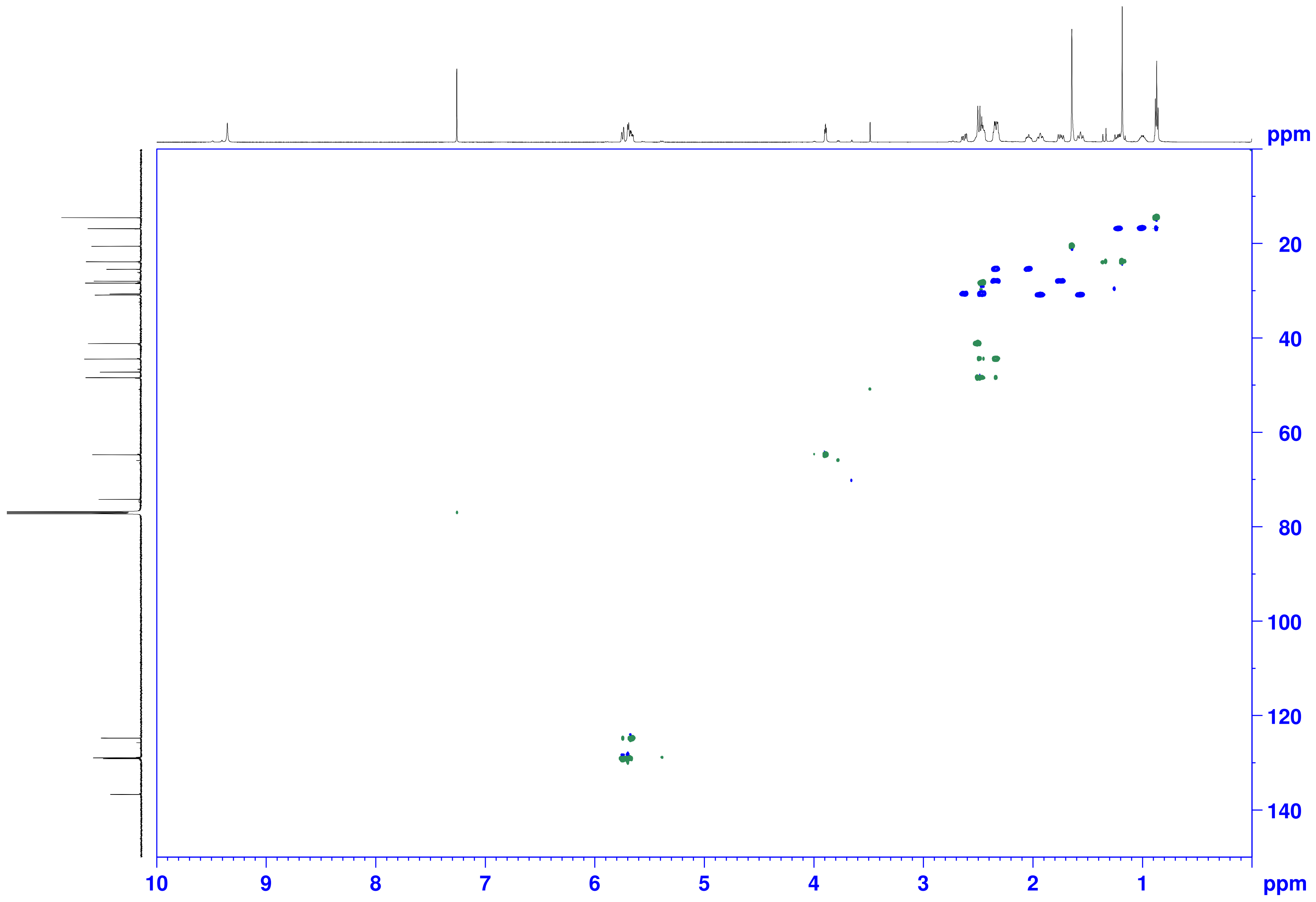

Figure S47. HSQC spectrum of **5** in $CDCl_3$.

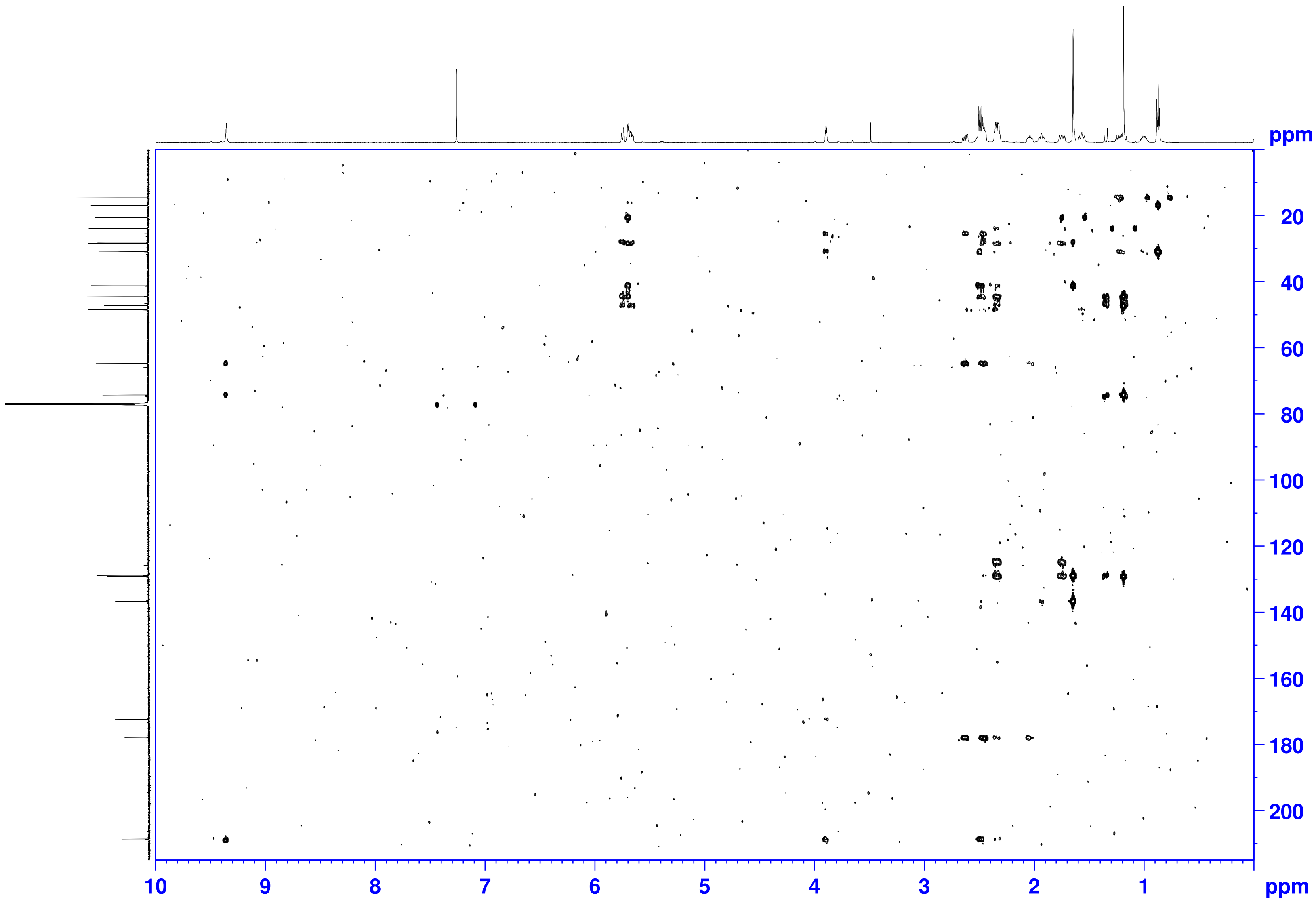

Figure S48. HMBC spectrum of **5** in $CDCl_3$.

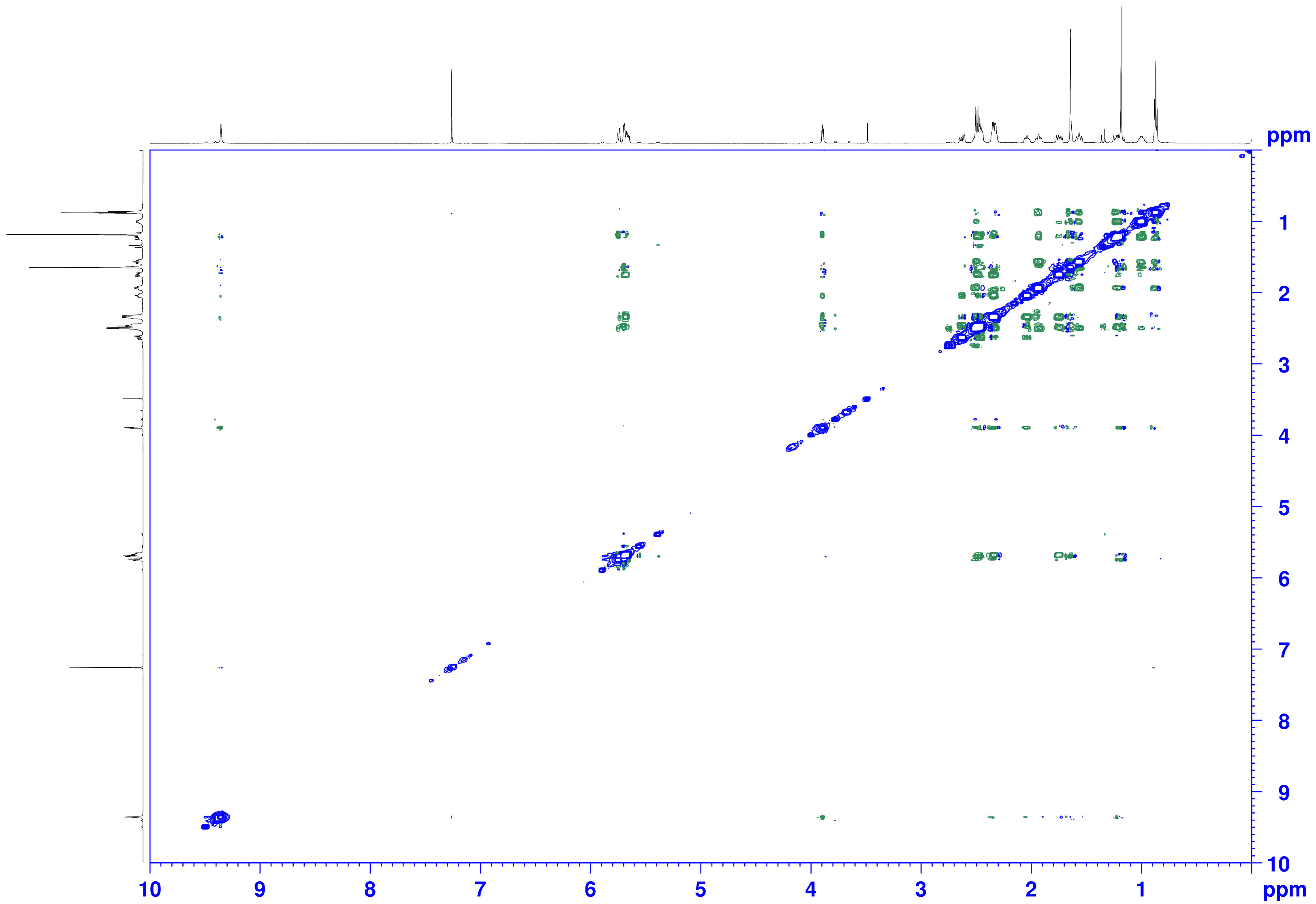

Figure S49. NOESY spectrum of **5** in $CDCl_3$.

Supplementary References

(1) Tang, J.; Matsuda, Y., Discovery of branching meroterpenoid biosynthetic pathways in *Aspergillus insuetus*: Involvement of two terpene cyclases with distinct cyclization modes. *Chem. Sci.* **2022**, *13*, 10361-10369.

(2) (a) Uhlén, M.; Fagerberg, L.; Hallström, B. M.; Lindskog, C.; Oksvold, P.; Mardinoglu, A.; Sivertsson, Å.; Kampf, C.; Sjöstedt, E.; Asplund, A.; Olsson, I.; Edlund, K.; Lundberg, E.; Navani, S.; Szigyarto, C. A.-K.; Odeberg, J.; Djureinovic, D.; Takanen, J. O.; Hober, S.; Alm, T.; Edqvist, P.-H.; Berling, H.; Tegel, H.; Mulder, J.; Rockberg, J.; Nilsson, P.; Schwenk, J. M.; Hamsten, M.; von Feilitzen, K.; Forsberg, M.; Persson, L.; Johansson, F.; Zwahlen, M.; von Heijne, G.; Nielsen, J.; Pontén, F., Tissue-based map of the human proteome. *Science* **2015**, *347*, 1260419; (b) *The Human Protein Atlas*; The Human Protein Atlas. https://www.proteinatlas.org/ (accessed 2025-09-07); (c) *The Human Protein Atlas*; The human proteome in druggable. https://www.proteinatlas.org/humanproteome/tissue/druggable/ (accessed 2025-09-07).

(3) Tang, J.; Matsuda, Y., Discovery of fungal onoceroid triterpenoids through domainless enzyme-targeted global genome mining. *Nat. Commun.* **2024**, *15*, 4312.

(4) Buchfink, B.; Reuter, K.; Drost, H.-G., Sensitive protein alignments at tree-of-life scale using DIAMOND. *Nat. Methods* **2021**, *18*, 366-368.

(5) Stanke, M.; Diekhans, M.; Baertsch, R.; Haussler, D., Using native and syntenically mapped cDNA alignments to improve *de novo* gene finding. *Bioinformatics* **2008**, *24*, 637-644.

(6) O'Leary, N. A.; Cox, E.; Holmes, J. B.; Anderson, W. R.; Falk, R.; Hem, V.; Tsuchiya, M. T. N.; Schuler, G. D.; Zhang, X.; Torcivia, J.; Ketter, A.; Breen, L.; Cothran, J.; Bajwa, H.; Tinne, J.; Meric, P. A.; Hlavina, W.; Schneider, V. A., Exploring and retrieving sequence and metadata for species across the tree of life with NCBI Datasets. *Scientific Data* **2024**, *11*, 732.

(7) (a) Navarro-Muñoz, J. C.; Selem-Mojica, N.; Mullowney, M. W.; Kautsar, S. A.; Tryon, J. H.; Parkinson, E. I.; De Los Santos, E. L. C.; Yeong, M.; Cruz-Morales, P.; Abubucker, S.; Roeters, A.; Lokhorst, W.; Fernandez-Guerra, A.; Cappelini, L. T. D.; Goering, A. W.; Thomson, R. J.; Metcalf, W. W.; Kelleher, N. L.; Barona-Gomez, F.; Medema, M. H., A computational framework to explore large-scale biosynthetic diversity. *Nat. Chem. Biol.* **2020**, *16*, 60-68; (b) Draisma, A.; Loureiro, C.; Louwen, N. L. L.; Kautsar, S. A.; Navarro-Muñoz, J. C.; Doering, D. T.; Mouncey, N. J.; Medema, M. H., BiG-SCAPE 2.0 and BiG-SLiCE 2.0: Scalable, accurate and interactive sequence clustering of metabolic gene clusters. *bioRxiv* **2025**, August 25, 2025. DOI: 10.1101/2025.08.20.671210.

(8) Wei, X.; Matsuyama, T.; Sato, H.; Yan, D.; Chan, P. M.; Miyamoto, K.; Uchiyama, M.; Matsuda, Y., Molecular and computational bases for spirofuranone formation in setosusin biosynthesis. *J. Am. Chem. Soc.* **2021**, *143*, 17708-17715.

(9) Chen, L.; Tang, J.-W.; Liu, Y. Y.; Matsuda, Y., Aspcandine: A pyrrolobenzazepine alkaloid synthesized by a fungal nonribosomal peptide synthetase-polyketide synthase hybrid. *Org. Lett.* **2022**, *24*, 4816-4819.

(10) Matsuda, Y.; Bai, T.; Phippen, C. B. W.; Nødvig, C. S.; Kjærbølling, I.; Vesth, T. C.; Andersen, M. R.; Mortensen, U. H.; Gotfredsen, C. H.; Abe, I.; Larsen, T. O., Novofumigatonin biosynthesis involves a non-heme iron-dependent endoperoxide isomerase for orthoester formation. *Nat. Commun.* **2018**, *9*, 2587.

(11) Ishii, J.; Kondo, T.; Makino, H.; Ogura, A.; Matsuda, F.; Kondo, A., Three gene expression vector sets for concurrently expressing multiple genes in *Saccharomyces cerevisiae*. *FEMS Yeast Res.* **2014**, *14*, 399-411.

(12) Lonhienne, T.; Low, Y. S.; Garcia, M. D.; Croll, T.; Gao, Y.; Wang, Q.; Brillault, L.; Williams, C. M.; Fraser, J. A.; McGeary, R. P.; West, N. P.; Landsberg, M. J.; Rao, Z.; Schenk, G.; Guddat, L. W., Structures of fungal and plant acetohydroxyacid synthases. *Nature* **2020**, *586*, 317-321.

(13) Wiederhold, N. P., Antifungal susceptibility of yeasts and filamentous fungi by CLSI broth microdilution testing. In *Antifungal Drug Resistance: Methods and Protocols*, Krysan, D. J.; Moye-Rowley, W. S., Eds. Springer US: New York, NY, 2023; pp 3-16.

(14) Liao, P.; Lung, S.-C.; Chan, W. L.; Bach, T. J.; Lo, C.; Chye, M.-L., Overexpression of HMG-CoA synthase promotes *Arabidopsis* root growth and adversely affects glucosinolate biosynthesis. *J. Exp. Bot.* **2019**, *71*, 272-289.

(15) Gilchrist, C. L. M.; Chooi, Y.-H., clinker & clustermap.js: Automatic generation of gene cluster comparison figures. *Bioinformatics* **2021**, *37*, 2473-2475.
